# ZEP1 orchestrates template choice and crossover pathways to ensure meiotic genome integrity in rice

**DOI:** 10.1038/s44318-026-00910-0

**Published:** 2026-09-17

**Authors:** Jie Zhang, Chong Wang, Yingzhu Xiao, Xiaofei Chen, James D Higgins, Wanqi Liang

**Affiliations:** 1 Joint International Research Laboratory of Metabolic & Developmental Sciences, School of Life Sciences and Biotechnology, Shanghai Jiao Tong University, Shanghai, 200240 China; 2 Department of Genetics and Genome Biology, University of Leicester, Leicester, LE1 7RH United Kingdom; 3 Yazhou Bay Institute of Deepsea Sci-Tech, Shanghai Jiao Tong University, Sanya, 572024 China

**Keywords:** Cell Cycle, DNA Replication, Recombination & Repair, Plant Biology

## Abstract

Meiotic crossovers (COs) are tightly regulated to ensure chromosome segregation while limiting aberrant recombination. Transverse filament (TF) proteins of the synaptonemal complex (SC) regulate class I crossoverts, yet how the SC coordinates homologous recombination (HR) to maintain faithful recombination remains unclear. Here, we show that loss of rice TF protein ZEP1 does not uniformly enhance HEI10-marked class I COs; instead, ZEP1 null mutants display asynapsis, multivalent formation, and chromosome fragmentation. These defects depend on double-strand breaks (DSBs) and genetically place ZEP1 function after strand invasion. ZEP1 interacts with anti-crossover factors MEICA1, FIGNL1, and RMI1, and is required for their enrichment at the synaptonemal complex; its loss causes persistent DMC1/RAD51 signals, indicating dysregulated strand invasion. Genetic interactions further support impaired recombination intermediate homeostasis, with ZMM removal partially alleviating chromosome abnormalities, while MUS81 becomes increasingly essential. We propose that the synaptonemal complex functions as a structural hub, where ZEP1 concentrates anti-crossover modules that restrain invasion and coordinate recombination intermediate processing to promote HR repair. Furthermore, ZEP1 dosage may provide a constrained lever to tune CO outcomes in a background-dependent manner.

## Synopsis

Transverse filament proteins are conserved synaptonemal complex scaffolds that influence meiotic crossover formation. This study shows that rice ZEP1 coordinates anti-crossover activities and crossover pathways to promote proper intermediate processing and faithful recombination.

- The rice synaptonemal complex transverse filament protein ZEP1 suppresses aberrant chromosome associations and fragmentation during male meiosis.
- Loss of ZEP1 affects HEI10-marked class I crossover designation and maturation in a genetic background-dependent manner.
- ZEP1 promotes the enrichment of anti-crossover factors at the central region of the synaptonemal complex, facilitating the proper processing of interhomolog recombination intermediates.
- ZEP1 coordinates anti-crossover activities with class I and class II crossover pathways to promote homolog-biased HR repair and ensure faithful chromosome segregation.

## Abstract (web summary)

A conserved transverse-filament protein of the synaptonemal complex coordinates anti-crossover activities and crossover pathways to promote proper intermediate processing and faithful recombination.

## Graphical abstract

**Figure:**
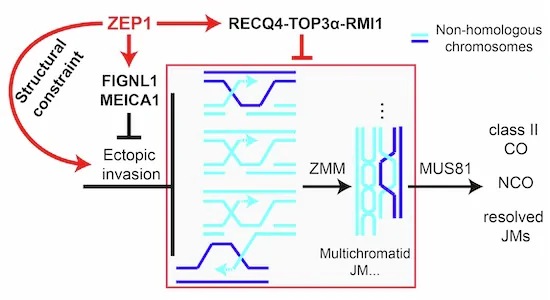

## Introduction

Homologous recombination (HR) preserves meiotic genome integrity while generating genetic diversity. It is triggered by programmed DNA double-strand breaks (DSBs) catalyzed by the topoisomerase-like SPO11/MTOPVIB complex together with accessory factors (Jing et al, 2019). DSB ends are resected to generate 3’ single-stranded DNA (ssDNA) overhangs that are first coated by RPA and subsequently replaced by the recombinases RAD51 and DMC1. These recombinases promote strand invasion into a homologous duplex to form recombination intermediates. Crossover (CO)–designated intermediates are stabilized and matured by ZMM proteins to yield class I, interference-sensitive COs, whereas a minority of intermediates are resolved by structure-selective nucleases such as MUS81 to form class II, interference-insensitive COs (Wang and Copenhaver, 2018). Although DSBs vastly outnumber final COs (~150–250 versus ~10 in *Arabidopsis* (Wang and Copenhaver, 2018)), each chromosome pair nevertheless acquires at least one obligate CO to ensure bivalent formation, highlighting stringent control over CO number and distribution by both pro- and anti-recombination (anti-CO) pathways. Mutations in ZMM genes eliminate class I COs and result in univalents (Lynn et al, 2007), whereas mutations in anti-CO factors increase MUS81-dependent class II COs and can restore bivalents in class I CO-defective backgrounds (Crismani et al, 2012; Fernandes et al, 2018; Girard et al, 2015).

Anti-CO activity is mediated by three genetically parallel pathways: (1) the FANCM helicase with MHF1/MHF2, (2) RECQ4 helicase (yeast Sgs1/human BLM)–Topoisomerase 3α–RMI1 complexes (STR in yeast, BTR in human, RTR in *Arabidopsis*), and (3) FIGL1–FLIP complex. FANCM and Sgs1/BLM/RECQ4, together with their partner proteins, favor non-crossover (NCO) outcomes by dismantling D-loops or dissolving double Holliday junctions, thereby limiting meiotic CO formation (Crismani et al, 2012; De Muyt et al, 2012; Fasching et al, 2015; Girard et al, 2014; Séguéla-Arnaud et al, 2015; Zakharyevich et al, 2012). In addition to restricting COs, STR/RTR complexes prevent the accumulation of aberrant recombination intermediates (joint molecules, JMs; including multichromatid JMs) and promote chromosome disentanglement, enabling faithful homolog segregation and genome stability (Jessop and Lichten, 2008; Kaur et al, 2015; Oh et al, 2007; Oh et al, 2008; Tang et al, 2015). Consistently, loss of RTR components in plants causes pronounced meiotic defects (Chelysheva et al, 2008; Hartung et al, 2008; Higgins et al, 2011; Séguéla-Arnaud et al, 2015). The conserved FIGL1–FLIP complex reduces strand-invasion capacity by modulating RAD51/DMC1 activity (Fernandes et al, 2018; Girard et al, 2015; Yuan and Chen, 2013). In rice, disruption of FIGNL1/MEICA1 leads to severe chromosomal abnormalities during meiosis, emphasizing their essential role in completing DSB repair (Hu et al, 2017; Yang et al, 2022; Zhang et al, 2017).

Meiotic prophase I is characterized by homolog pairing, synapsis, and recombination. Pairing is facilitated by recombination complexes that are physically and functionally linked to chromosome axes (Zickler and Kleckner, 2015). Synapsis is achieved through assembly of the synaptonemal complex (SC), a multi-layered protein scaffold composed of two lateral elements (one along each homolog) connected by transverse filament (TF) proteins across the central region (CR). TF proteins form parallel homodimers, with their N termini positioned near the SC center and their C termini anchored in lateral elements (Cahoon et al, 2017; Page and Hawley, 2004). Although TF proteins are required for synapsis, homologs can still co-align at larger distances when synapsis fails (Capilla-Pérez et al, 2021; France et al, 2021).

Across species, TF proteins promote class I CO formation and are therefore functionally aligned with ZMM activity. Loss of TF proteins disrupts SC assembly and reduces class I CO formation, generating achiasmate univalents in budding yeast (ZIP1), fruit fly (C(3)G), nematodes (SYP1–6), mouse (SYCP1) (Colaiácovo et al, 2003; de Vries et al, 2005; Hurlock et al, 2020; MacQueen et al, 2002; Page and Hawley, 2001; Schild-Prüfert et al, 2011; Smolikov et al, 2007; Smolikov et al, 2009; Storlazzi et al, 1996; Sym et al, 1993; Zhang et al, 2020). Notably, partial loss-of-function TF alleles can produce the opposite effect. A separation-of-function ZIP1 allele in yeast and partial SYP1 depletion in nematodes increase class I COs while weakening CO interference (Libuda et al, 2013; Voelkel-Meiman et al, 2016). In rice, a hypomorphic ZEP1 allele (*zep1-3*) causes asynapsis but shows elevated class I COs and reduced interference, yet male meiosis still produces normal tetrads (Wang et al, 2015; Wang et al, 2010). By contrast, partial ZYP1 suppression in *Arabidopsis* and barley reduces class I CO frequency, whereas the *Arabidopsis zyp1* null mutant increases class I COs, reduces interference, and abolishes heterochiasmy (Barakate et al, 2014; Capilla-Pérez et al, 2021; France et al, 2021; Higgins et al, 2005). Together, these findings suggest that TF proteins can impose species- and dosage-dependent control over class I CO number and patterning, and that differences between null and hypomorphic alleles may reflect partial functional retention and network sensitivity in distinct genetic contexts.

In plants, TF loss can also increase multivalent formation, as observed in rice ZEP1 knockouts and partially suppressed *Arabidopsis* ZYP1 lines (Barakate et al, 2014; Capilla-Pérez et al, 2021; France et al, 2021; Higgins et al, 2005; Liu et al, 2021; Wang et al, 2010), implying additional, underappreciated roles of TF proteins in DNA repair. How TF proteins collaborate with HR factors to ensure completion of DSB repair, and how the SC coordinates recombination intermediates to bias CO versus NCO outcomes, remain unresolved. Here, we analyze TF mutants in rice and find that disruption of ZEP1 does not increase HEI10-marked class I CO numbers in any cultivar tested. Instead, ZEP1 loss abolishes synapsis and causes multivalents, univalents, and chromosome fragmentation. We show that these defects arise from failure to complete HR-mediated DSB repair after strand invasion. Mechanistically, ZEP1 directly interacts with anti-CO factors and is required for their recruitment to the SC, where they regulate strand invasion and processing of recombination intermediates to promote homologous recombination. We propose that the SC protects CO fate and genome integrity by coordinating anti-CO activities through the transverse filament.

## Results

### ZEP1 disruption causes aberrant meiotic chromosome associations and segregation defects

Using map-based cloning and whole-genome resequencing of our rice mutant library (Chen et al, 2006), we identified a new *zep1*^9522^ allele in *O. sativa* ssp. *Japonica* cv. 9522. The mutant carries a single thymine insertion in the *ZEP1* gene (LOC_Os04g37960), causing a frameshift and premature stop codon (Appendix Fig. S1). In wild-type male meiocytes, ZEP1 appears as puncta at leptotene, elongates into short linear signals at zygotene, and forms continuous tracks between full-length chromosome axes at pachytene (Appendix Fig. S2A) (Wang et al, 2010). By contrast, immunostaining with anti-ZEP1 together with the axis marker REC8 (Shao et al, 2011) detected no ZEP1 signal in *zep1*^9522^ meiocytes (Appendix Fig. S2B), and immunoblotting confirmed loss of ZEP1 protein (Appendix Fig. S2C). These features differ from the hypomorphic *zep1-3* allele, which produces a truncated protein with aberrant localization (Wang et al, 2015; Wang et al, 2010) (Appendix Fig. S2). *zep1*^9522^ plants exhibited normal vegetative growth but complete male sterility, producing thin, pale anthers lacking viable pollen (Appendix Fig. S3A–D). Female gametogenesis was largely preserved, with only a mild reduction in embryo sac viability (~84% [58 out of 69 spikelets] in *zep1*^9522^ versus ~97% [38 out of 39 spikelets] in wild type; Appendix Fig. S3E,F), consistent with prior reports that ZEP1 loss primarily disrupts male fertility (Liu et al, 2021). We refer to this null allele hereafter as *zep1*^*null*^*-1*.

**Figure 1:**
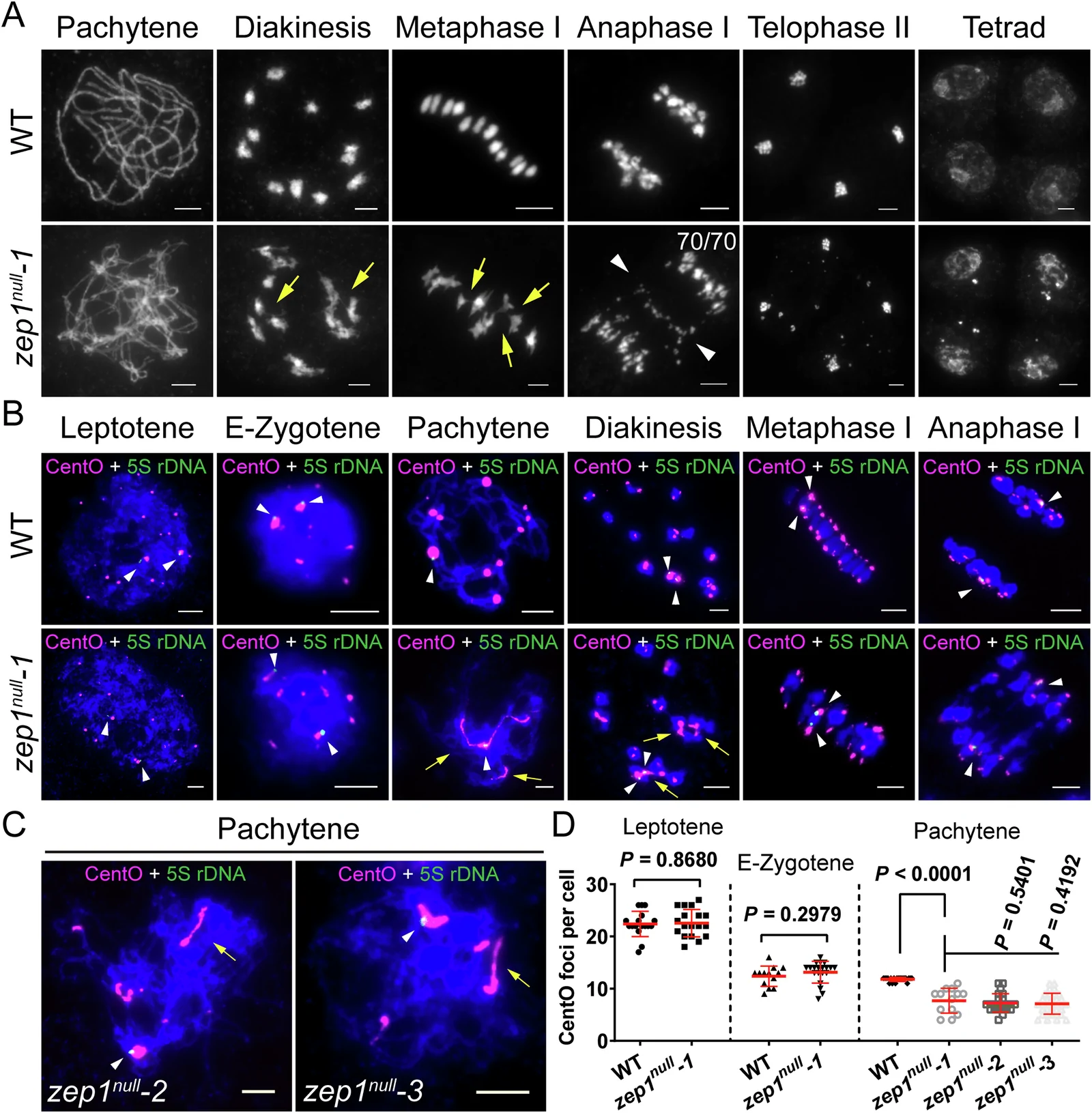
ZEP1 is dispensable for homologous bivalent formation but is required to repress aberrant male meiotic recombination in rice. (**A**) Defective chromosomal behaviors were observed at different meiosis stages in *zep1* male meiocytes compared with those in wild type (WT). Meiotic defects are indicated by yellow arrows (abnormal chromosome connections) and white arrowheads (chromosome fragments), respectively. Meiotic chromosomes are visualized by DAPI staining. Numbers in the panel indicate the number of meiocytes displaying the indicated phenotype over the total number of analyzed meiocytes. A total of *n* = 70 anaphase I meiocytes were scored from chromosome spreads prepared from pooled meiotic florets collected from independent plants of the same genotype. Multiple independent slide preparations were analyzed. Scale bars, 5 μm. (**B**, **C**) FISH analysis using CentO (magenta) and 5S rDNA (green, white arrowheads) probes in wild-type and *zep1*^*null*^ (*zep1*^*null*^*-1*, *zep1*^*null*^*-2*, *zep1*^*null*^*-3*) meiocytes at different meiotic stages. Meiotic chromosomes are visualized by DAPI staining (blue). Yellow arrows indicate linearly arranged centromeres. Scale bars, 5 μm. (**D**) CentO foci numbers in wild-type and *zep1*^*null*^ male meiocytes. A total of *n* = 17 WT leptotene meiocytes, *n* = 18 *zep1*^*null*^*-1* leptotene meiocytes, *n* = 13 WT early zygotene (E-zygotene) meiocytes, *n* = 20 *zep1*^*null*^*-1* early zygotene (E-zygotene) meiocytes, *n* = 20 WT pachytene meiocytes, *n* = 13 *zep1*^*null*^*-1* pachytene-like meiocytes, *n* = 23 *zep1*^*null*^*-2* pachytene-like meiocytes, and *n* = 32 *zep1*^*null*^*-3* pachytene-like meiocytes were scored from chromosome spreads generated using pooled anthers dissected from meiotic florets collected from independent plants of the same genotype. Multiple independent slide preparations were examined to confirm the reproducibility of the observed phenotypes, with all quantified cells incorporated into the plotted dataset. Each dot corresponds to a single meiocyte. Bars denote mean ± SD of the quantified cell population. Statistical significance was determined via a two-tailed Student’s *t*-test. Statistical *P* values derived from the corresponding quantitative analyses are indicated within the figure panels. Exact *P* values are as follows: WT versus *zep1*^*null*^*-1* at leptotene, *P* = 0.8680; WT versus *zep1*^*null*^*-1* at early zygotene, *P* = 0.2979; WT versus *zep1*^*null*^*-1* at pachytene, *P* < 0.0001; *zep1*^*null*^*-1* versus *zep1*^*null*^*-2* at pachytene, *P* = 0.5401; and *zep1*^*null*^*-1* versus *zep1*^*null*^*-3* at pachytene, *P* = 0.4192. For comparisons in which GraphPad Prism returned *P* values of <0.0001, values are reported as *P* < 0.0001. Source data underlying this figure are provided online.

**Figure EV1:**
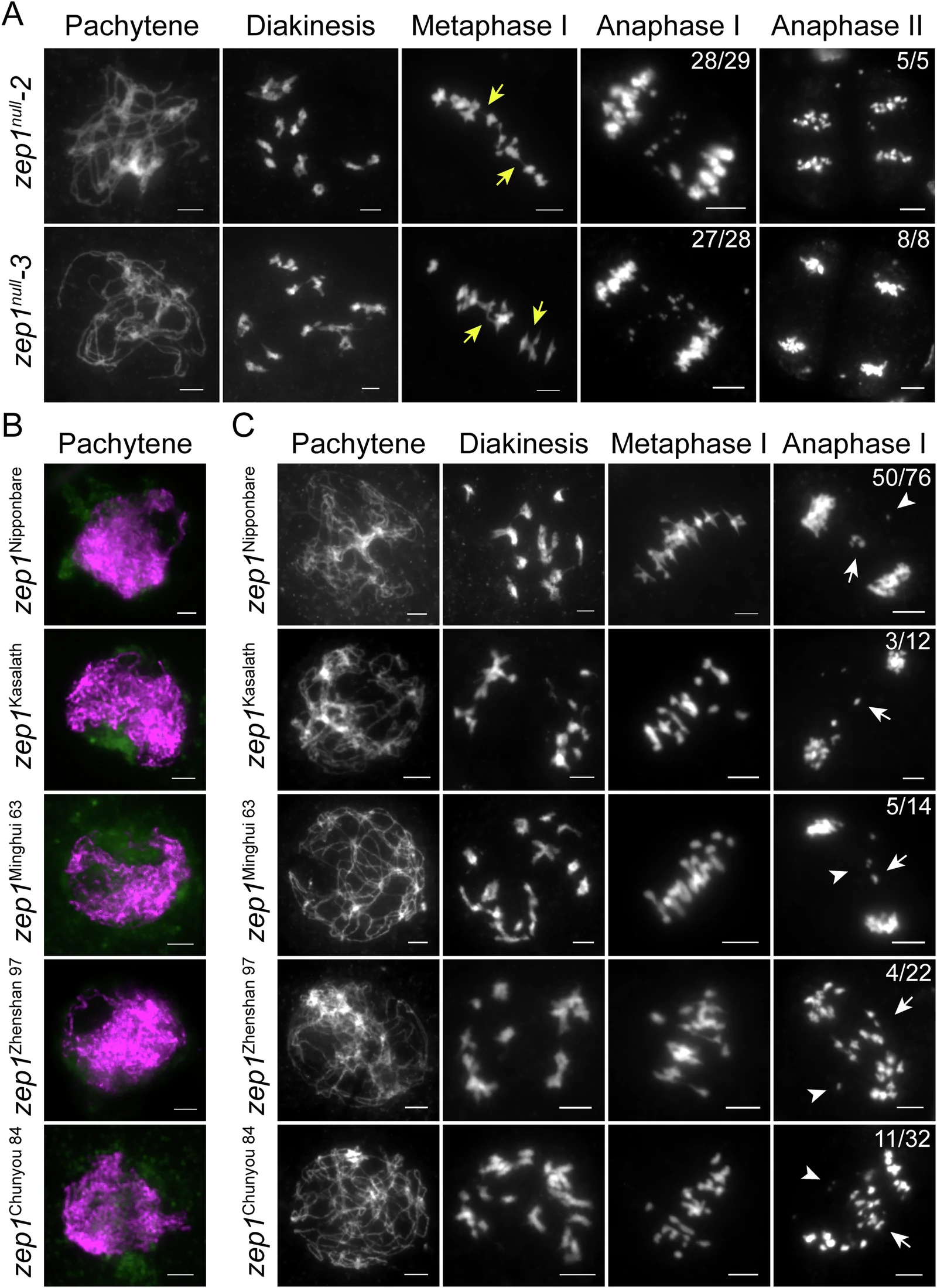
*zep1* null mutation leads to abnormal chromosome behaviors in all tested rice varieties. (**A**) Chromosome behavior during different stages of male meiosis in *zep1*^*null*^ CRISPR lines in *japonica* 9522 background (*zep1*^*null*^*-2* and *zep1*^*null*^*-3*). Abnormal chromosome connections are indicated by yellow arrows. Scale bar, 5 μm. Arabic numerals in the panels represent the number of representative images containing chromosome fragments relative to the total number of observations. A total of *n* = 29 *zep1*^*null*^*-2*, *n* = 28 *zep1*^*null*^*-3* anaphase I and *n* = 5 *zep1*^*null*^*-2*, *n* = 8 *zep1*^*null*^*-3* anaphase II meiocytes were scored from chromosome spreads of pooled anthers isolated from meiotic florets of independent isogenic plants. Multiple independent slide preparations were examined to confirm the reproducibility of the observed phenotypes. Meiotic chromosomes are visualized by DAPI staining. (**B**) No obvious ZEP1 protein foci (green) were detected on *zep1* male meiotic chromosomes. Meiotic chromosomes are visualized by axis protein REC8 (magenta). Scale bar, 2 μm. (**C**) Chromosome behavior during different stages of male meiosis in *zep1* mutants from different genetic backgrounds. Meiotic defects are indicated by white arrows (univalents) and white arrowheads (chromosome fragments), respectively. Meiotic chromosomes are visualized by DAPI staining. Arabic numerals in the panels represent the number of representative images containing chromosome fragments relative to the total number of observations. A total of *n* = 76 *zep1*^Nipponbare^, *n* = 12 *zep1*^Kasalath^, *n* = 14 *zep1*^Minghui 63^, *n* = 22 *zep1*^Zhenshan 97^, *n* = 32 *zep1*^Chunyou 84^ anaphase I meiocytes were scored from chromosome spreads of pooled anthers isolated from meiotic florets of independent isogenic plants. Multiple independent slide preparations were analyzed. Scale bar, 5 μm. Source data underlying this figure are provided online.

To define how ZEP1 loss impacts male meiotic progression, we examined chromosome behavior throughout meiosis by DAPI (4’,6-diamidino-2-phenylindole) staining. Wild-type and *zep1*^*null*^*-1* chromosome behaviors appeared similar from leptotene to early zygotene, but at the pachytene-like stage, *zep1*^*null*^*-1* showed separated thin threads rather than fully synapsed thick threads, consistent with asynapsis (Fig. 1A). From diakinesis to metaphase I, *zep1*^*null*^*-1* frequently displayed twisted bivalents and multivalents, whereas univalents were comparatively rare (Fig. 1A; Appendix Fig. S3G). Wild-type metaphase I meiocytes contained 12 bivalents. In contrast, *zep1*^*null*^*-1* (*n* = 146 cells) averaged ~3.62 bivalents, ~0.12 univalents, and ~8.32 chromosome pairs engaged in one or more multivalent structures (Figs. 1A and 3B; Appendix Fig. S3G). At anaphase I, lagging chromosome fragments were consistently observed near the equatorial plate (70/70), followed by unequal segregation and micronuclei at tetrad stage (Fig. 1A). We observed similar defects in two independent CRISPR-generated null mutants (*zep1*^*null*^*-2* and *zep1*^*null*^*-3*) carrying frameshift in the ZEP1 N- or C-terminal globular domains in the same *japonica* 9522 background (Fig. EV1A, Appendix Figs. S2, S4, and S5). At metaphase I, *zep1*^*null*^*-2* (*n* = 52) averaged ~3.21 bivalents, ~0.08 univalents, and ~8.75 multivalents; *zep1*^*null*^*-3* (*n* = 40) averaged ~3.10, ~0.10, and ~8.85, respectively. Chromosome fragments and lagging chromosomes were subsequently detected during later meiotic stages (Figs. 3B and EV1A). Together, these results indicate that male sterility in *zep1*^*null*^ mutants reflects unrepaired meiotic DNA lesions that promote chromosome entanglement and fragmentation.

### ZEP1 suppresses non-homologous chromosome recombination without preventing homolog pairing and recombination

To assess homolog association and segregation, we performed fluorescent in situ hybridization (FISH) with the CentO probe (centromeres of all chromosomes) and a 5S rDNA probe (short arm of chromosome 11) (Zhang et al, 2019). During leptotene to early zygotene, CentO signal numbers decreased similarly in wild type (22.4 [*n* = 17] to 12.4 [*n* = 13]) and *zep1*^*null*^*-1* (22.6 [*n* = 18] to 13.2 [*n* = 20]) (Fig. 1B,D), consistent with centromere coupling during early prophase I. Two separated 5S signals were frequently observed at this stage (9/13 wild type; 14/20 *zep1*^*null*^*-1*), suggesting that early centromere associations can involve non-homologous chromosomes (Fig. 1B). At the pachytene-like stage, two adjacent 5S signals were detected in both genotypes (*n* = 51), indicating that homolog pairing per se was not disrupted in *zep1*^*null*^*-1* (Fig. 1B). However, CentO behavior differed sharply at the pachytene-like stage. Wild-type nuclei showed ~12 dispersed CentO signals (11.8 ± 0.09, *n* = 20), whereas *zep1*^*null*^ meiocytes frequently exhibited stretched CentO signals and a significant reduction in total signal number (7.7 ± 0.66 [*n* = 13] in *zep1*^*null*^*-1*, 7.3 ± 0.37 [*n* = 23] in *zep1*^*null*^*-2* and 7.1 ± 0.35 [*n* = 32] in *zep1*^*null*^*-3*) (Fig. 1B–D). At diakinesis (*n* = 41) and metaphase I (*n* = 48), the two 5S signals consistently resided within a single DAPI-stained entity, whereas stretched CentO signals were associated with multivalents (Fig. 1B). At anaphase I, two individual 5 s rDNA signals migrated to the opposite poles of the cell (*n* = 17) (Fig. 1B). Importantly, lagging fragments lacked both 5S (*n* = 17) and CentO (*n* = 14) signals, indicating that they are acentric (Fig. 1B). Thus, ZEP1 is dispensable for establishing homolog pairing but is required to prevent inappropriate non-homologous recombination that generates multivalents.

### ZEP1 loss alters HEI10 dynamics and class I CO designation in a background-dependent manner

**Figure 2:**
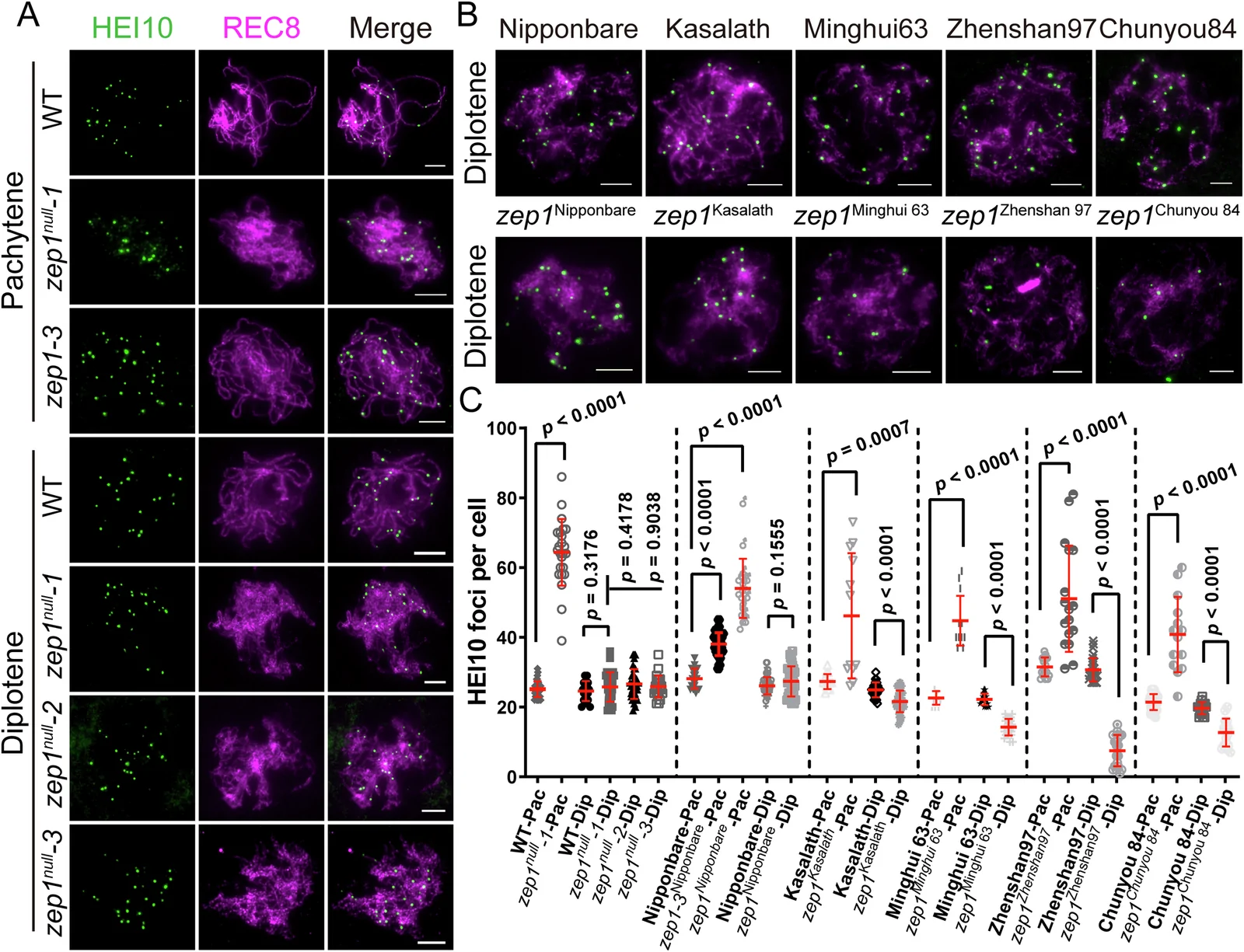
Dynamics and number of HEI10 protein foci in wild-type and *zep1* null male meiocytes from different rice varieties. (**A**) Immunodetection of HEI10 protein foci (green) at prophase I (from pachytene [or pachytene-like] to diplotene stage) in wild-type and three *zep1*^null^ mutants (*O. sativa* ssp. *Japonica* cv. 9522), and *ZEP1* Tos17 mutant (*zep1-3*, *O. sativa* ssp. *Japonica* cv. Nipponbare). Scale bars, 5 μm. (**B**) Immunodetection of HEI10 protein foci (green) in wild-type and other *zep1* null mutants (*O. sativa* ssp. *Japonica* cv. Nipponbare, *O. sativa ssp. Indica* cv. Kasalath/Minghui 63/Zhenshan 97 and inter-subspecific hybrid rice Chunyou 84) at the diplotene stage. Meiotic chromosomes are visualized by axis protein REC8 (magenta). Scale bars, 5 μm. (**C**) The number of HEI10 protein foci per meiocyte at pachytene or pachytene-like (Pac) and diplotene (Dip) stages was quantified in three *zep1*^null^ mutants (*O. sativa* ssp. *Japonica* cv. 9522), the *ZEP1* Tos17 insertion mutant (*zep1-3*, *O. sativa* ssp. *Japonica* cv. Nipponbare), and additional *zep1* null mutants (*O. sativa* ssp. *Japonica* cv. Nipponbare, *O. sativa ssp. Indica* cv. Kasalath/Minghui 63/Zhenshan 97 and inter-subspecific hybrid rice Chunyou 84), together with their respective wild-type plants. A total of *n* = 35 WT pachytene meiocytes, n = 24 *zep1*^*null*^*-1* pachytene-like meiocytes, *n* = 17 WT diplotene meiocytes, *n* = 32 *zep1*^*null*^*-1* diplotene meiocytes, *n* = 35 *zep1*^*null*^*-2* diplotene meiocytes, *n* = 24 *zep1*^*null*^*-3* diplotene meiocytes in the 9522 background; *n* = 19 WT pachytene meiocytes, n = 27 *zep1-3* pachytene-like meiocytes, *n* = 19 *zep1* pachytene-like meiocytes, *n* = 31 WT diplotene meiocytes, *n* = 36 *zep1* diplotene meiocytes in the Nipponbare background; *n* = 14 WT pachytene meiocytes, *n* = 12 *zep1* pachytene-like meiocytes, *n* = 25 WT diplotene meiocytes, *n* = 33 *zep1* diplotene meiocytes in the Kasalath background; *n* = 13 WT pachytene meiocytes, *n* = 15 *zep1* pachytene-like meiocytes, *n* = 15 WT diplotene meiocytes, *n* = 26 *zep1* diplotene meiocytes in the Minghui 63 background; *n *= 17 WT pachytene meiocytes, *n* = 17 *zep1* pachytene-like meiocytes, *n* = 29 WT diplotene meiocytes, *n* = 19 *zep1* diplotene meiocytes in the Zhenshan 97 background; *n* = 24 WT pachytene meiocytes, *n* = 14 *zep1* pachytene-like meiocytes, *n* = 19 WT diplotene meiocytes, *n *= 15 *zep1* diplotene meiocytes in the Chunyou 84 background were scored from chromosome spreads of pooled anthers isolated from meiotic florets of independent isogenic plants. Multiple independent slide preparations were examined to confirm the reproducibility of the observed phenotypes, with all quantified cells included in the plotted dataset. Each dot corresponds to a single meiocyte. Bars denote mean ± SD of the quantified cell population. Statistical significance was determined via a two-tailed Student’s *t*-test. Statistical *P* values derived from the corresponding quantitative analyses are indicated within the figure panels. Exact *P* values are as follows: for the 9522 background, WT versus *zep1*^*null*^*-1* at pachytene, *P* < 0.0001; WT versus *zep1*^*null*^*-1* at diplotene, *P* = 0.3176; *zep1*^*null*^*-1* versus *zep1*^*null*^*-2* at diplotene, *P* = 0.4178; and *zep1*^*null*^*-1* versus *zep1*^*null*^*-3* at diplotene, *P* = 0.9038. For the Nipponbare background, WT versus *zep1-3* at pachytene, *P* < 0.0001; WT versus *zep1* at pachytene, *P* < 0.0001; and WT versus *zep1* at diplotene, *P* = 0.1555. For the Kasalath background, WT versus *zep1* at pachytene, *P* = 0.0007, and at diplotene, *P* < 0.0001. For the Minghui 63 background, WT versus *zep1* at pachytene, *P* < 0.0001, and at diplotene, *P* < 0.0001; For the Zhenshan 97 background. WT versus *zep1* at pachytene, *P* < 0.0001, and at diplotene, *P* < 0.0001. For the Chunyou 84 background, WT versus *zep1* at pachytene, *P* < 0.0001, and at diplotene, *P* < 0.0001. For comparisons in which GraphPad Prism returned *P* values of <0.0001, values are reported as *P* < 0.0001. Source data underlying this figure are provided online.

Because increased HEI10‑marked class I COs were reported for the rice hypomorphic *zep1* allele, we monitored HEI10 dynamics to test whether ZEP1 loss similarly elevates class I COs (Wang et al, 2015; Wang et al, 2012). In paraformaldehyde-fixed meiocytes, *zep1*^*null*^*-1* mutants showed zygotene HEI10 accumulation comparable to wild type. Consistent with asynapsis, HEI10 failed to organize into the linear tracks typical of pachytene wild type, and bright HEI10 foci appeared later, during diplotene-diakinesis. At diakinesis, bright HEI10 foci numbers did not differ between wild type (22.2 ± 0.49 [*n* = 29]) and *zep1*^*null*^*-1* (22.3 ± 0.52 [*n* = 22]; *p* = 0.8417 [Student’s *t-* test]) (Appendix Fig. S6). To improve staging in asynaptic meiocytes, we performed parallel HEI10 analysis in Carnoy’s-fixed samples (Chelysheva et al, 2010). At the late pachytene-like stage, *zep1*^*null*^*-1* exhibited a mixture of wild-type-like and dim HEI10 foci, resulting in a higher total focus count (64.3 ± 1.9, *n* = 24) than bright foci in wild type (25.1 ± 0.38 [*n* = 35]) at the equivalent stage (Fig. 2A,C). By diplotene, however, HEI10 foci were similar in number and intensity across genotypes (24.6 ± 0.69 [*n* = 17] in wild type versus 25.8 ± 0.75 [*n* = 32] in *zep1*^*null*^*-1*, 26.6 ± 0.72 [*n* = 35] in *zep1*^*null*^*-2*, and 25.9 ± 0.64 [*n* = 24] in *zep1*^*null*^*-3*) (Fig. 2A,C). Thus, in the *japonica* 9522 background, ZEP1 loss does not change the final number of HEI10 foci but delays the progression of HEI10-marked intermediates toward maturation.

**Figure 3:**
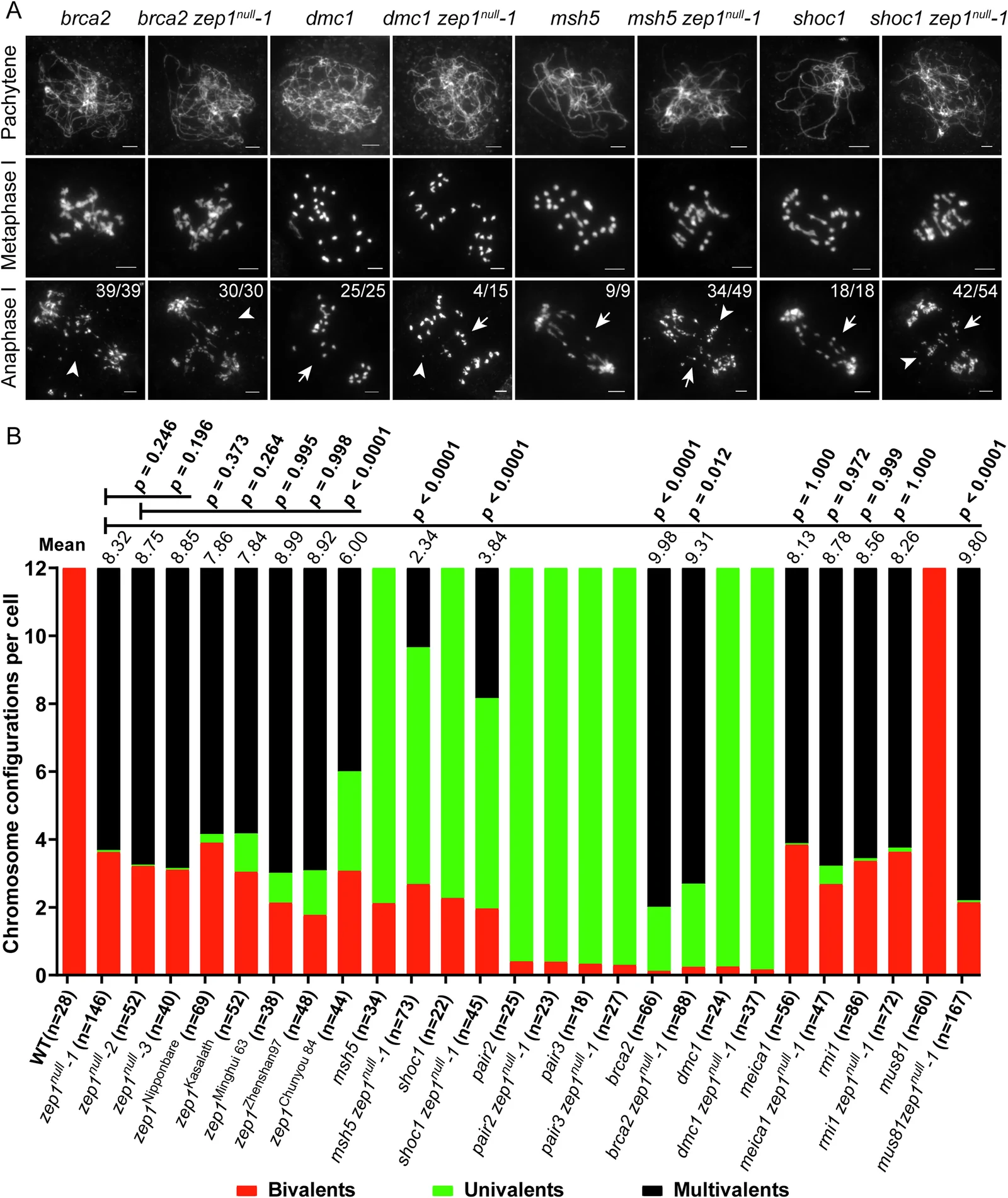
Abnormal meiosis progression in *zep1*^*null*^ depends on strand-invasion during male meiotic DSB repair in rice. (**A**) Representative chromosome behavior from pachytene (or pachytene-like) to anaphase I in key meiosis-related single mutants and ZEP1-associated double mutants. Meiotic defects are indicated by white arrows (univalents) and white arrowheads (chromosome fragments), respectively. Arabic numerals in the panels represent the number of representative images containing univalents/chromosome fragments relative to the total number of observations. A total of *n* = 39 *brca2*, *n* = 30 *brca2 zep1*^*null*^*-1*, *n* = 25 *dmc1*, *n* = 15 *dmc1 zep1*^*null*^*-1*, *n* = 9 *msh5*, *n* = 49 *msh5 zep1*^*null*^*-1*, *n* = 18 *shoc1*, and *n* = 54 *shoc1 zep1*^*null*^*-1* anaphase I meiocytes were scored from chromosome spreads generated via multiple independent slide preparations using pooled anthers isolated from meiotic florets collected from independent plants of the same genotype. Meiotic chromosomes are visualized by DAPI staining. Scale bars, 5 μm. (**B**) Chromosome configurations at meiotic metaphase I in selected single and double mutants. Chromosome configurations are presented relative to the twelve chromosome pairs of *O. sativa*. A total of *n* = 28 WT, *n* = 146 *zep1*^*null*^*-1*, *n* = 52 *zep1*^*null*^*-2*, *n* = 40 *zep1*^*null*^*-3*, *n* = 69 *zep1*^Nipponbare^, *n* = 52 *zep1*^Kasalath^, *n* = 38 *zep1*^Minghui 63^, *n* = 48 *zep1*^Zhenshan 97^, *n* = 44 *zep1*^Chunyou 84^, *n* = 34 *msh5*, *n* = 73 *msh5 zep1*^*null*^*-1*, *n* = 22 *shoc1*, *n* = 45 *shoc1 zep1*^*null*^*-1*, *n* = 25 *pair2*, *n* = 23 *pair2 zep1*^*null*^*-1*, *n* = 18 *pair3*, *n* = 27 *pair3 zep1*^*null*^*-1*, *n* = 66 *brca2*, *n* = 88 *brca2 zep1*^*null*^*-1*, *n* = 24 *dmc1*, *n* = 37 *dmc1 zep1*^*null*^*-1*, *n* = 56 *meica1*, *n* = 47 *meica1 zep1*^*null*^*-1*, *n* = 86 *rmi1*, *n* = 72 *rmi1 zep1*^*null*^*-1*, *n* = 60 *mus81*, *n* = 167 *mus81 zep1*^*null*^*-1* metaphase I meiocytes were scored from chromosome spreads generated using pooled anthers dissected from meiotic florets collected from independent plants of the same genotype. Multiple independent slide preparations were analyzed, with all quantified cells incorporated into the plotted dataset. The number of cells sampled for each line is shown in brackets. Mean values were analyzed by one-way ANOVA. Post hoc Fisher’s LSD tests were used for comparisons with *zep1*^*null*^*-1* when equal variances were assumed, whereas Games–Howell tests were used for comparisons with *zep1*^*null*^*-1* and *zep1*^*null*^*-2* when variances were unequal. *P* < 0.0001, statistical tests yielded *P* values below 0.0001 for this comparison. Statistical *P* values derived from the corresponding quantitative analyses are indicated within the figure panels. Source data underlying this figure are provided online.

To test whether this behavior generalizes across rice germplasm, we generated *ZEP1* knockouts (targeting the same N-terminal region) in five additional cultivars: one *japonica* (Nipponbare), three *indica* (Kasalath, Minghui 63 and Zhenshan 97), and one inter-subspecific hybrid Chunyou 84 (Appendix Fig. S7; Fig. EV1B). In every background, ZEP1 loss caused defective male meiosis with variable mixtures of univalents, bivalents, multivalents, and chromosome fragments, culminating in male sterility (Fig. EV1C; Appendix Fig. S7). At metaphase I, mutants in single-subspecies backgrounds resembled *zep1*^null^*-2*, with averages of ~7.86 (*n* = 69; *zep1*^Nipponbare^), ~7.84 (*n* = 52; *zep1*^Kasalath^), ~8.99 (*n* = 38; *zep1*^Minghui 63^), and ~8.92 (*n* = 48; *zep1*^Zhenshan 97^) chromosome pairs forming multivalents; the hybrid background *zep1*^Chunyou 84^ showed fewer multivalents (~6.00, *n* = 44) (Figs. 3B and EV1C). Notably, univalents were much more frequent in *indica* and hybrid mutants (~2.26 in *zep1*^Kasalath^, ~1.76 in *zep1*^Minghui 63^, ~2.62 in *zep1*^Zhenshan 97^, and ~5.86 in *zep1*^Chunyou 84^) than in *japonica* mutants (~0.50 in *zep1*^Nipponbare^ and ~0.08 in *zep1*^*null*^*-2*) (Fig. 3B), suggesting cultivar-dependent impairment of CO assurance.

Consistent with prior work (Wang et al, 2015), the hypomorphic *zep1-3*^Nipponbare^ allele showed increased bright HEI10 foci at the late pachytene-like stage (38.0 ± 0.63 [*n* = 27] versus 28.2 ± 0.66 [*n* = 19] in Nipponbare) (Fig. 2A–C). Across all ZEP1 mutants, late pachytene-like meiocytes exhibited significantly increased total HEI10 foci (54.0 ± 1.95 [*n* = 19] in *zep1*^Nipponbare^, 46.2 ± 5.18 [*n* = 12] in *zep1*^Kasalath^, 44.7 ± 1.85 [*n* = 15] in *zep1*^Minghui 63^, 51.1 ± 3.70 [*n* = 17] in *zep1*^Zhenshan 97^, and 40.8 ± 2.89 [*n* = 14] in *zep1*^Chunyou 84^) relative to matched wild types (28.2 ± 0.66 [*n* = 19], 27.3 ± 0.58 [*n* = 14], 22.6 ± 0.54 [*n* = 13], 31.5 ± 0.65 [*n* = 17], and 21.4 ± 0.47 [*n* = 24], respectively) (Fig. 2C; Appendix Fig. S8). However, at diplotene, prominent HEI10 focus numbers diverged by genetic background: *zep1*^Nipponbare^ remained similar to wild type (27.3 ± 0.72 [*n* = 36] versus 26.1 ± 0.45 [*n* = 31]), whereas *indica* and hybrid mutants showed significant reductions (21.6 ± 0.55 [*n* = 33] in *zep1*^Kasalath^, 14.2 ± 0.47 [*n* = 26] in *zep1*^Minghui 63^, 7.5 ± 1.04 [*n* = 19] in *zep1*^Zhenshan 97^, and 12.7 ± 1.04 [*n* = 15] in *zep1*^Chunyou 84^) compared with their wild types (24.9 ± 0.42 [*n* = 25], 22.2 ± 0.43 [*n* = 15], 30.7 ± 0.62 [*n* = 29], and 19.6 ± 0.40 [*n* = 19]) (Fig. 2B,C). Therefore, ZEP1 loss does not universally increase the number of designated class I COs marked by HEI10 foci; instead, their numbers are either unchanged or reduced depending on the cultivar background.

### ZEP1 acts after strand invasion to enable SEI repair with or without ZMM factors

**Figure EV2:**
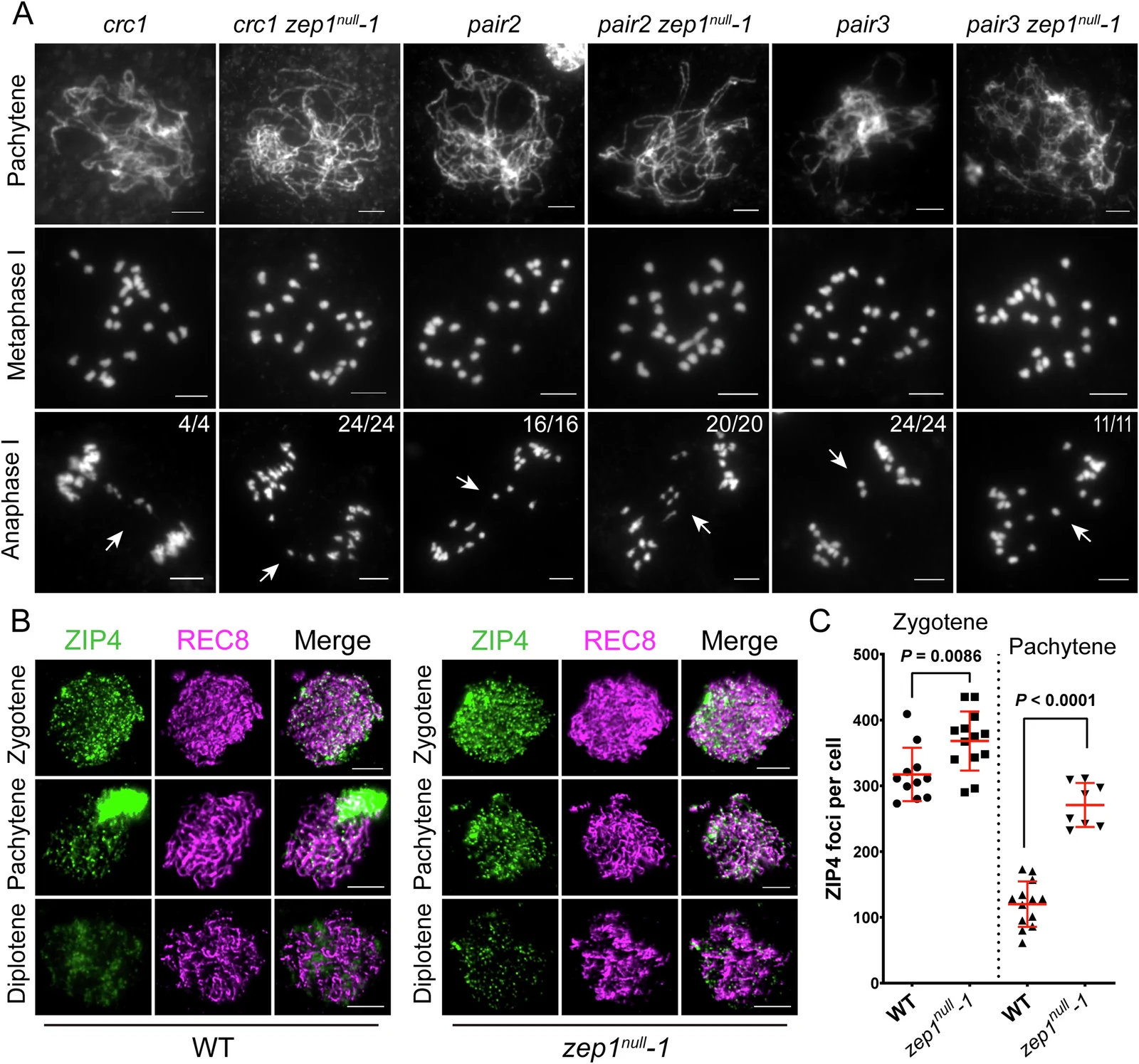
ZEP1 acts downstream of homologous pairing and modulates the chromosomal localization of ZIP4 protein during male meiosis. (**A**) Representative chromosome behavior from pachytene (or pachytene-like) to anaphase I in key meiosis-related single mutants and ZEP1-associated double mutants. Meiotic defects are indicated by white arrows (univalents) and white arrowheads (chromosome fragments), respectively. Arabic numerals in the panels represent the number of representative images containing univalents/chromosome fragments relative to the total number of observations. A total of *n* = 4 *crc1*, *n* = 24 *crc1 zep1*^*null*^*-1*, *n* = 16 *pair2*, *n* = 20 *pair2 zep1*^*null*^*-1*, *n* = 24 *pai3* and *n* = 11 *pair3 zep1*^*null*^*-1* anaphase I meiocytes were scored from chromosome spreads generated via multiple independent slide preparations using pooled anthers isolated from meiotic florets collected from independent plants of the same genotype. Meiotic chromosomes are visualized by DAPI staining. Scale bars, 5 μm. (**B**) Immunofluorescent signals of the ZMM protein ZIP4 (green) were detected in wild-type and *zep1*^*null*^*-1* male meiocytes at different substages of meiotic prophase I. Meiotic chromosomes are visualized by axis protein REC8 (magenta). Scale bars, 2 μm. (**C**) ZIP4 foci numbers in wild-type and *zep1*^*null*^ meiocytes at zygotene and pachytene (or pachytene-like) stages. A total of *n* = 11 WT zygotene meiocytes, *n* = 13 *zep1*^*null*^*-1* zygotene meiocytes, *n* = 13 WT pachytene meiocytes, *n* = 8 *zep1*^*null*^*-1* pachytene-like meiocytes were scored from chromosome spreads generated using pooled anthers dissected from meiotic florets collected from independent plants of the same genotype. Multiple independent slide preparations were examined to confirm the reproducibility of the observed phenotypes, with all quantified cells incorporated into the plotted dataset. Each dot corresponds to a single meiocyte. Bars denote mean ± SD of the quantified cell population. Statistical significance was determined via a two-tailed Student’s *t*-test. Statistical *P* values derived from the corresponding quantitative analyses are indicated within the figure panels. Exact *P* values are as follows: WT versus *zep1*^*null*^*-1* at zygotene, *P* = 0.0086; and WT versus *zep1*^*null*^*-1* at pachytene, *P* < 0.0001. For comparisons in which GraphPad Prism returned *P* values of <0.0001, values are reported as *P* < 0.0001. Source data underlying this figure are provided online.

The atypical meiotic defects observed in *zep1*^*null*^*-1*, including chromosome multivalents and chromosome fragments (Fig. 1), suggest abnormalities in meiotic DNA repair. To determine whether these defects depend on programmed DSB formation, we genetically disrupted DSB formation in the *zep1*^*null*^*-1* background. In rice, meiotic DSB formation requires SDS and CRC1, and mutations in either gene produce 24 univalents (Miao et al, 2013; Wu et al, 2015). Double mutants of *zep1*^*null*^*-1* with *sds* and *crc1* also produced 24 univalents and completely lacked chromosome interactions, demonstrating that multivalents and fragmentation in *zep1*^*null*^*-1* depend on meiotic DSB formation (Fig. EV2A; Appendix Fig. S9A). These results indicate that the defective repair phenotype in z*ep1*^*null*^*-1* arises during the recombinational repair of programmed meiotic DSBs.

To position ZEP1 within the HR pathway, we examined genetic interactions with pairing and strand-invasion factors. In rice, PAIR2 and PAIR3 are required for homolog pairing and synapsis, which provide the chromosomal context for efficient homologous recombination (Nonomura et al, 2006; Nonomura et al, 2004; Wang et al, 2011; Yuan et al, 2009). At metaphase I, the chromosome configurations of *pair2 zep1*^*null*^*-1* (~23.22 univalents and ~0.39 bivalents; *n* = 23) and *pair3 zep1*^*null*^*-1* (~23.40 univalents and ~0.30 bivalents; *n* = 27) closely resembled those of the corresponding single mutants (*pair2*: ~23.20 univalents and ~0.40 bivalents, n = 25; *pair3*: ~23.34 univalents and ~0.33 bivalents, *n* = 18). Moreover, no chromosome fragments were detected at anaphase I in either *pair2 zep1*^*null*^*-1* (*n* = 20) or *pair3 zep1*^*null*^*-1* (*n* = 11), as was also observed in the corresponding single mutants (*pair2*, *n* = 16; *pair3*, *n* = 24) (Figs. 3B and EV2A), indicating that aberrant chromosome associations require PAIR2/PAIR3-dependent homolog pairing or pairing-associated recombination progression. These data suggest that ZEP1 acts after the establishment of the pairing-competent chromosomal context, rather than at the earliest step of homolog pairing itself.

We next examined genetic interactions with factors directly involved in strand invasion. Rice BRCA2 promotes RAD51/DMC1-mediated strand invasion, and *brca2* mutants show asynapsis, severe chromosome entanglements, and fragmentation or bridges (Fu et al, 2020). At metaphase I, *brca2* mutants exhibited an average of ~0.12 bivalents, ~3.78 univalents, and ~9.98 chromosome pairs engaged in one or more multivalent structures (*n* = 66). Introduction of the *brca2* mutation into *zep1* significantly reduced bivalent formation, showing ~0.24 in *brca2 zep1*^*null*^*-1* (*n* = 88), compared with ~3.62 in *zep1*^*null*^*-1* (*n* = 146). This reduction was accompanied by an increase in univalents (~4.90 in *brca2 zep1*^*null*^*-1* versus ~0.12 in *zep1*^*null*^*-1*) and chromosome pairs engaged in multivalent structures (~9.31 in *brca2 zep1*^*null*^*-1* versus ~8.32 in *zep1*^*null*^*-1*). Chromosome fragments were also frequently observed in anaphase I cells of both the *brca2* single mutant (*n* = 39) and the *brca2 zep1*^*null*^*-1* double mutant (*n* = 30) (Fig. 3A,B). We further assessed the relationship between ZEP1 and the recombinases that catalyze invasion of intact homologous templates. RAD51C and DMC1 are required for efficient strand invasion during meiotic HR in rice (Tang et al, 2014; Wang et al, 2016). RAD51C loading was unchanged in *zep1*^*null*^*-1* (213.6 ± 4.71 [*n* = 17]) relative to wild type (202.9 ± 5.37 [*n* = 15]) (Appendix Fig. S9C,D), suggesting that ZEP1 is not required for initial RAD51C recruitment to meiotic chromosomes. In *dmc1 zep1*^*null*^*-1* double mutants, metaphase I abnormalities shifted toward the *dmc1* phenotype, with an average of ~0.16 bivalents and ~23.68 univalents in *dmc1 zep1*^*null*^*-1* (*n* = 37), similar to *dmc1* single mutants (~0.25 bivalents and ~23.5 univalents, *n* = 24). Nevertheless, univalents often showed irregular morphology and only a minority of anaphase I cells contained fragments (4/15) (Fig. 3A,B). Together, these results suggest that ZEP1 acts after strand invasion during HR-mediated DSB repair and may additionally limit aberrant repair involving sister chromatids.

ZMM proteins bind and stabilize nascent post-invasion intermediates and promote class I COs (Pyatnitskaya et al, 2019). In rice, *zmm* mutants complete synapsis but form many univalents at metaphase I. The chiasmata number in *msh5* (2.07 ± 0.16, *n* = 105) and *shoc1* (3.21 ± 0.43, *n* = 29) single mutants were similar to previous reports (2.10 ± 0.91 in *msh5* and 3.40 ± 1.89 in *shoc1*) (Luo et al, 2013; Zhang et al, 2019). In *msh5 zep1*^*null*^*-1* and *shoc1 zep1*^*null*^*-1* double mutants, the asynapsis, multivalents, and fragmentation resembled *zep1*^*null*^*-1* (Fig. 3A; Appendix Fig. S9B). Strikingly, univalent numbers were reduced relative to the *zmm* single mutants ( ~ 13.96 univalents in *msh5 zep1*^*null*^*-1* [*n* = 73] versus 19.76 in *msh5* [*n* = 34]; ~12.40 in *shoc1 zep1*^*null*^*-1* [*n* = 45] versus 19.46 in *shoc1* [*n* = 22]). Multivalents were also reduced compared with *zep1*^*null*^*-1* (~2.34 multivalent pairs in *msh5 zep1*^*null*^*-1*; ~3.84 in *shoc1 zep1*^*null*^*-1*; versus ~8.32 in *zep1*^*null*^*-1*) (Fig. 3A,B; Appendix Fig. S9B). Moreover, a subset of anaphase I cells in these double mutants lacked fragments (15/49 in *msh5 zep1*^*null*^*-1*; 12/54 in *shoc1 zep1*^*null*^*-1*), unlike *zep1*^*null*^*-1* (Fig. 3A). Thus, removing ZMM components partially suppresses abnormal chromosome connections and fragmentation in *zep1*^*null*^, consistent with ZEP1 coordinating downstream processing of strand-invasion intermediates, including those handled by the ZMM pathway. The ZMM protein ZIP4, a key regulator of class I CO formation in rice (Shen et al, 2012), showed slightly higher accumulation but significantly delayed removal in *zep1*^*null*^*-1* from zygotene to pachytene. Specifically, ZIP4 focus numbers were 317.2 ± 12.2 in wild type (*n* = 11), 368.0 ± 12.3 in zep*1*^*null*^*-1* (*n* = 13) at zygotene, but declined to 119.9 ± 9.6 in wild type at pachytene (*n* = 13), while remaining high at 270.8 ± 11.9 in *zep1*^*null*^*-1* (*n* = 8) (Fig. EV2B,C). The delayed disappearance of ZIP4 foci further supports the idea that ZMM-associated recombination intermediates persist abnormally in the absence of ZEP1, consistent with a role for ZEP1 in promoting the timely maturation or resolution of post-invasion recombination structures.

### ZEP1 couples SC architecture to anti-CO pathways through direct protein interactions

**Figure 4:**
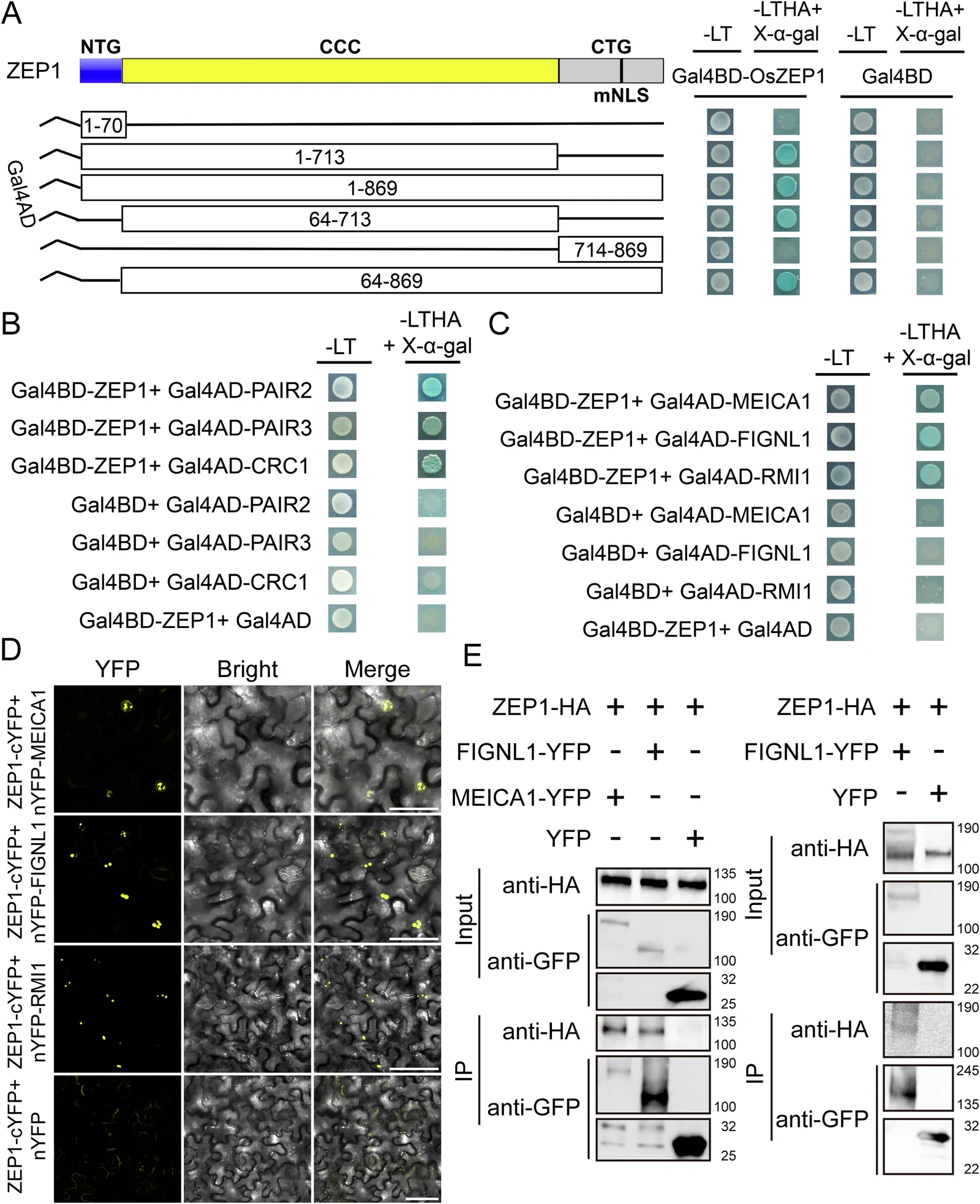
Rice ZEP1 interacts with SC components and anti-recombination proteins. (**A**) ZEP1 interacts with itself through its central coiled-coil region (CCC) in the Y2H (Yeast Two-Hybrid) assay. Positive interactions were assessed on selective media (SD–Leu/–Trp, −LT; SD–Leu/–Trp/–His/–Ade, −LTHA) supplemented with X-α-gal (blue colonies indicate interaction). The ZEP1 protein contains three different domains, including N- and C-terminal globular regions (NTG, aa 1–63; CTG, 714–869) and a central coiled-coil region (CCC, aa 64–713). (**B**) ZEP1 can interact with PAIR2/PAIR3 (axial/ lateral element protein of SC) and CRC1 (central region element protein of SC) in the Y2H assay. Blue colonies grown on quadruple dropout plates confirm protein–protein interactions. (**C**) Constructs co-expressing ZEP1 alongside MEICA1, FIGNL1 or RMI1 yielded blue reporter colonies, indicative of positive protein–protein interactions in the Y2H assay. (**D**) Bimolecular fluorescence complementation (BiFC) was employed to detect protein–protein interactions. ZEP1 was fused to the C-terminal YFP fragment (cYFP), while the proteins MEICA1, FIGNL1 and RMI1 were each separately fused to the N-terminal YFP fragment (nYFP). Pairs exhibiting positive interaction all produce punctate nuclear signals in tobacco cells. Scale bars, 50 μm. (**E**) An in vivo Co-IP assay in *N. benthamiana* leaf epidermal cells. “+” or “–” represent the presence or absence of the corresponding proteins. The numbers displayed on the right side of each panel indicate the molecular mass (kDa) of protein marker bands for the respective Western blot analyses of Co-IP experiments. Source data underlying this figure are provided online.

TF proteins typically dimerize via a coiled-coil region and span the SC width in an antiparallel arrangement (Page and Hawley, 2004). Their N-terminal globular domains localize to the SC central region, whereas their C-terminal globular domains anchor to lateral elements. Consistent with this architecture, ZEP1 formed homodimers via its coiled-coil region and interacted with lateral element proteins PAIR2 and PAIR3, as well as the central region component CRC1, in yeast two-hybrid (Y2H) assays (Fig. 4A,B), supporting a conserved structural role of TF protein in SC assembly during rice meiosis.

To identify factors through which ZEP1 may promote accurate DSB repair, we screened for interactions between ZEP1 and known meiotic proteins by Y2H assays, and these interactions were validated by selective growth and X-α-gal-dependent blue coloration as well as BiFC (Bimolecular fluorescence complementation) assays (Fig. 4C,D; Appendix Fig. S10). ZEP1 exhibited reproducible interactions in both assays with proteins involved in DNA damage response and recombination intermediate processing, including the DNA damage checkpoint factors RAD17 and RAD9, anti-CO factors MEICA1, FIGNL1 and RMI1, and the class II pro-CO factor MUS81. Because our genetic analyses placed ZEP1 downstream of strand invasion, and MEICA1, FIGNL1, and RMI1 are known regulators of post-strand-invasion recombination in plants (Chelysheva et al, 2008; Hartung et al, 2008; Hu et al, 2017; Séguéla-Arnaud et al, 2017; Yang et al, 2022), we selected these anti-CO factors for further validation by Co-IP (Co-Immunoprecipitation) assays (Fig. 4E). Together, these findings suggest that ZEP1 supports meiotic DSB repair not only by enabling synapsis but also by serving as a synaptonemal complex-associated interaction hub that links the transverse filament structure to recombination-regulatory pathways. In particular, its interaction with MEICA1, FIGNL1, and RMI1 suggests a mechanism by which ZEP1 may help coordinate anti-CO activities and thereby promote faithful processing of post-strand-invasion recombination intermediates.

### ZEP1 recruits MEICA1–FIGNL1 and RMI1 to restrain invasion and resolve atypical intermediates

**Figure EV3:**
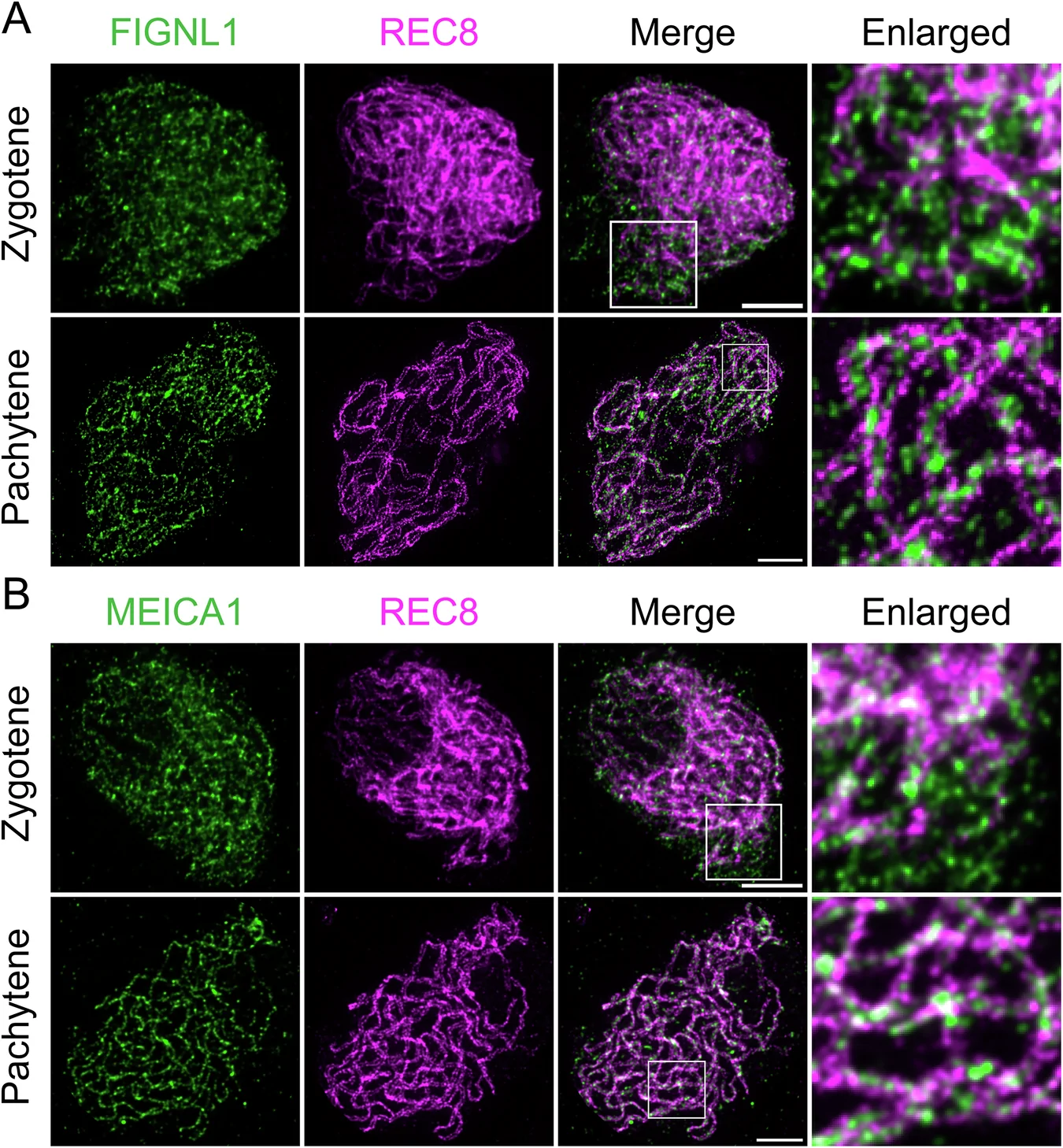
Dynamic co-localization of MEICA1 and FIGNL1 with the meiotic chromosome axis marker REC8 during prophase I. Stimulated emission depletion (STED) super-resolution microscopy of wild-type male prophase I meiocytes co-stained for the axis marker REC8 (magenta) together with FIGNL1 (**A**) or MEICA1 (**B**) (green) at zygotene and pachytene stages. Magnified insets of bracketed regions are shown on the right. Scale bars, 2 μm. Source data underlying this figure are provided online.

**Figure 5:**
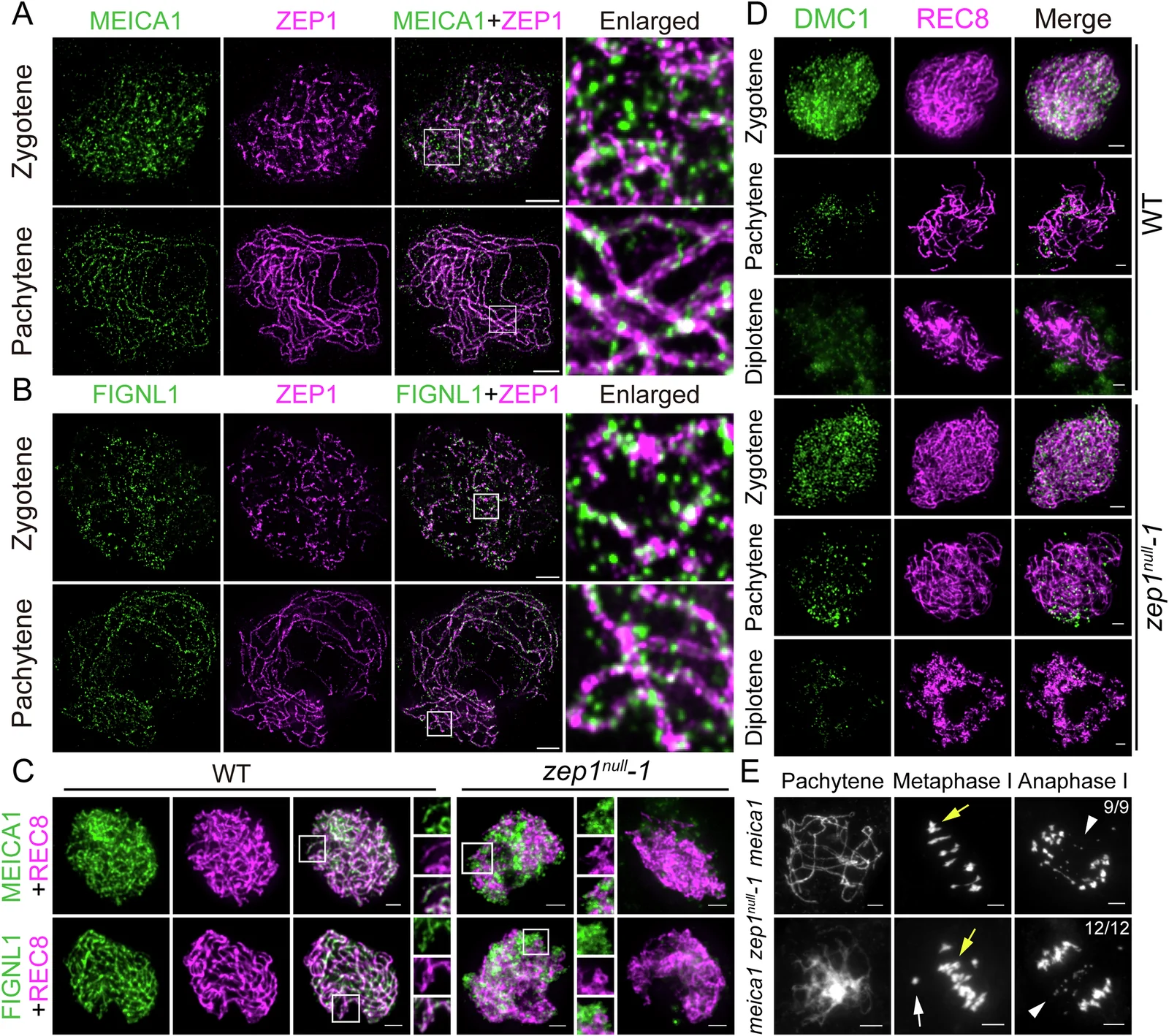
ZEP1 mediates the recruitment of the MEICA1 and FIGNL1 proteins to synaptonemal complexes in rice male meiocytes. (**A**, **B**) STED (Stimulated emission depletion) super-resolution imaging of ZEP1 (magenta) and MEICA1 (**A**) or FIGNL1 (**B**) (green) in wild-type male meiocytes at prophase I. Magnified insets of bracketed regions are displayed on the right. Scale bars, 2 μm. (**C**) The failed chromosomal or extrachromosomal enrichment of MEICA1 and FIGNL1 protein foci (green) was evidenced by immunodetection analysis in *zep1*^*null*^*-1* pachytene-like meiocytes. Images on the right showed local chromosomal enlargement marked by brackets in panel (**C**). Meiotic chromosomes are visualized by axis protein REC8 (magenta). Scale bars, 2 μm. Representative images shown here were obtained from the same experiment as the images presented in Appendix Fig. S11, which provides a more comprehensive comparison with additional representative examples. (**D**) Immunodetection of strand-invasion protein DMC1 (green) and meiotic chromosome axis marker REC8 (magenta) in wild-type and *zep1*^*null*^*-1* male meiocytes. Scale bars, 2 μm. (**E**) Chromosome behavior during different meiosis stages in *meica1* and *meica1 zep1*^*null*^*-1* male meiocytes. Meiotic chromosomes are visualized by DAPI staining. Meiotic defects are indicated by yellow arrows (abnormal chromosome connections), white arrows (univalents) and white arrowheads (chromosome fragments), respectively. Arabic numerals in the panels represent the number of representative images containing chromosome fragments or bridges relative to the total number of observations. A total of *n* = 9 *meica1*, and *n* = 12 *meica1 zep1*^*null*^*-1* anaphase I meiocytes were scored from chromosome spreads generated via multiple independent slide preparations using pooled anthers isolated from meiotic florets of independent isogenic plants. Scale bars, 5 μm. Source data underlying this figure are provided online.

**Figure EV4:**
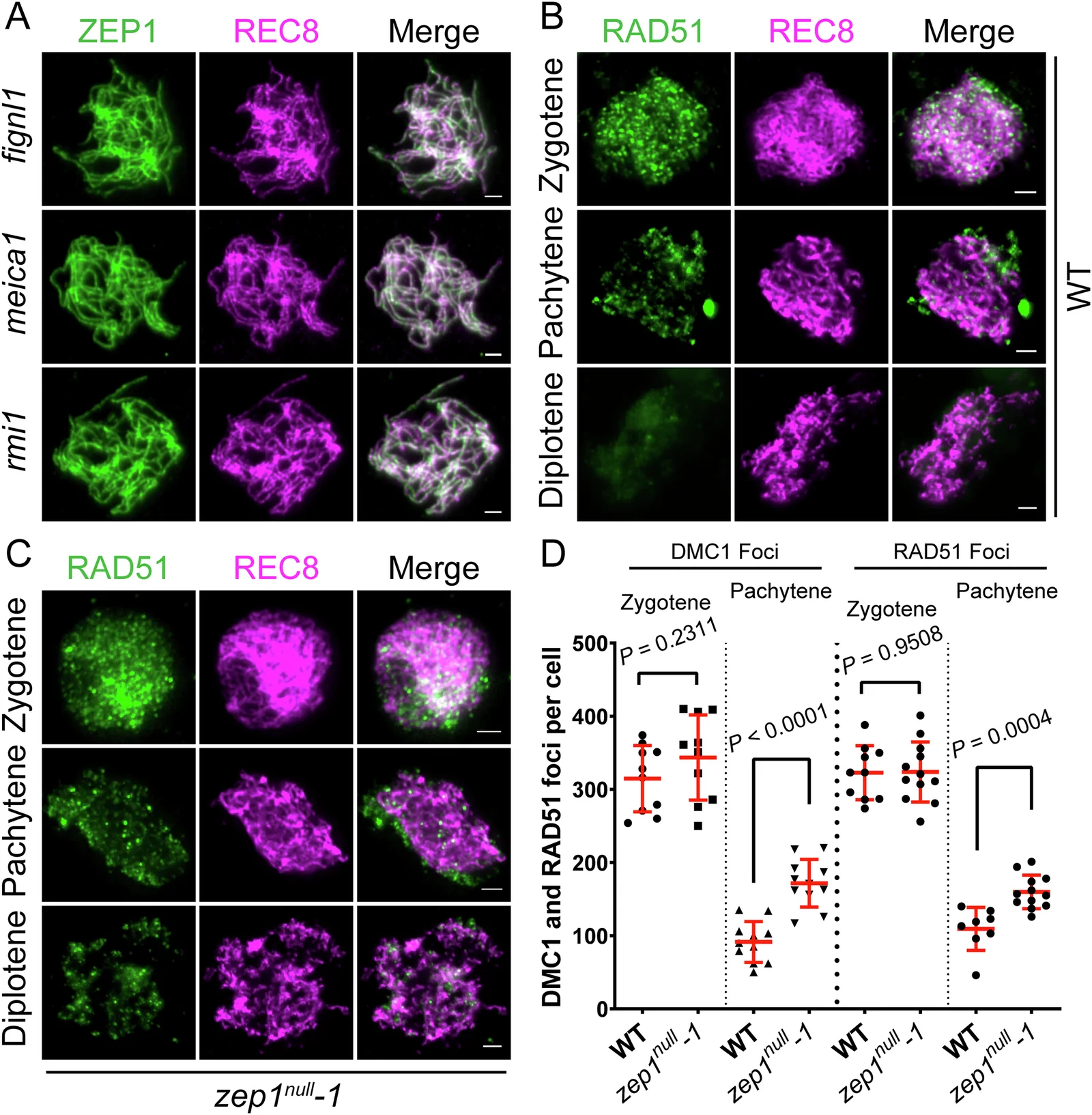
ZEP1 depletion delays dissociation of strand-invasion proteins from male meiotic chromosomes. (**A**) Mutations in MEICA1, FIGNL1 or RMI1 do not alter chromosomal loading of ZEP1 (green). Meiotic chromosomes are visualized by axis protein REC8 (magenta). Scale bars, 2 μm. (**B**,** C**) Immunofluorescent detection of the strand-invasion protein RAD51 (green) and meiotic chromosome axes (anti-OsREC8, magenta) in wild-type (**B**) and mutant (**C**) at different substages of meiotic prophase I. Scale bars, 2 μm. (**D**) DMC1 and RAD51 foci numbers in wild-type and *zep1*^*null*^*-1* meiocytes at zygotene and pachytene (or pachytene-like) stages. A total of *n* = 10 WT zygotene meiocytes, *n* = 10 *zep1*^*null*^*-1* zygotene meiocytes, *n* = 11 WT pachytene meiocytes, and *n* = 11 *zep1*^*null*^*-1* pachytene-like meiocytes were scored for DMC1 foci. For RAD51 foci, *n* = 10 WT zygotene meiocytes, *n* = 12 *zep1*^*null*^*-1* zygotene meiocytes, *n* = 8 WT pachytene meiocytes, and *n* = 12 *zep1*^*null*^*-1* pachytene-like meiocytes were scored. Meiocytes were scored from chromosome spreads generated using pooled anthers dissected from meiotic florets collected from independent plants of the same genotype. Multiple independent slide preparations were examined to confirm the reproducibility of the observed phenotypes, with all quantified cells incorporated into the plotted dataset. Each dot corresponds to a single meiocyte. Bars denote mean ± SD of the quantified cell population. Statistical significance was determined via a two-tailed Student’s *t*-test. Statistical *P* values derived from the corresponding quantitative analyses are indicated within the figure panels. Exact *P* values are as follows: for DMC1 foci, WT versus *zep1*^*null*^*-1* at zygotene, *P* = 0.2311, and at pachytene, *P* < 0.0001; for RAD51 foci, WT versus *zep1*^*null*^*-1* at zygotene, *P* = 0.9508, and at pachytene, *P* = 0.0004. For comparisons in which GraphPad Prism returned *P* values of <0.0001, values are reported as *P* < 0.0001. Source data underlying this figure are provided online.

To determine whether ZEP1 influences the chromosomal recruitment of these interacting post-strand-invasion regulators, we first generated polyclonal antibodies against MEICA1 and FIGNL1. In rice, MEICA1 and FIGNL1 interact and are interdependently loaded onto chromosomes, and loss of either protein causes similar meiotic defects (Hu et al, 2017; Yang et al, 2022). In early prophase I, MEICA1 and FIGNL1 showed a primary localization pattern adjacent to the REC8-marked chromosome axis. By pachytene, these signals became concentrated between homologous chromosome axes (Fig. EV3). Co-immunostaining with ZEP1 further revealed that the overlap between extended ZEP1 signals and MEICA1/FIGNL1 foci increased progressively from zygotene to pachytene (Fig. 5A,B), indicating a close spatial and functional association between ZEP1 and the MEICA1–FIGNL1 complex during synaptonemal complex assembly. In *zep1*^*null*^*-1*, MEICA1 and FIGNL1 signals were strongly reduced in ~54% (*n* = 114) and ~52% (*n* = 165) of meiocytes, respectively; in the remaining cells, both proteins predominantly appeared as chromosome-disassociated signals. Comparable defects in MEICA1 and FIGNL1 localization were detected in independent CRISPR-generated ZEP1 null alleles (Fig. 5C; Appendix Fig. S11). In contrast, ZEP1 localization was not detectably disrupted in the absence of MEICA1 or FIGNL1 (Fig. EV4A), indicating that ZEP1 is required for efficient recruitment and/or stabilization of the MEICA1–FIGNL1 complex on the SC, whereas MEICA1, FIGNL1 is not required for ZEP1 assembly.

Consistent with impaired regulation of strand invasion, DMC1 and RAD51 accumulated excessively and persisted longer in *zep1*^*null*^*-1* meiocytes. At zygotene, initial loading of DMC1 was comparable between wild type and *zep1*^*null*^*-1*. DMC1 foci numbered 314.6 ± 14.3 in wild type (n = 10) and 343.5 ± 18.4 in *zep1*^*null*^*-1* (*n* = 10), while RAD51 foci numbered 322.7 ± 11.7 in wild type (*n* = 10) and 323.8 ± 11.9 in *zep1*^*null*^*-1* (*n* = 12). However, at the pachytene-like stage, both DMC1 and RAD51 showed delayed dissociation from chromosomes in *zep1*^*null*^*-1* with 171.7 ± 9.8 DMC1 foci (*n* = 11) and 159.9 ± 6.6 RAD51 foci (*n* = 12), compared with 91.5 ± 8.4 DMC1 foci (*n* = 11) and 109.3 ± 10.4 RAD51 (*n* = 8) in wild type. This persistence resembles prior observations in *meica1* and *fignl1* mutants (Figs. 5D; EV4B–D) (Yang et al, 2022). Given that *meica1* mutants display aberrant chromosome connections and segregation (Hu et al, 2017), we further analyzed *meica1 zep1*^*null*^*-1* double mutants. The asynapsis phenotype resembled that of *zep1*^*null*^*-1*, and the extent of multivalent formation at metaphase I was comparable among the double mutant and both single mutants: ∼8.78 chromosome pairs engaged in multivalent structures in *meica1 zep1*^*null*^*-1* (*n* = 47), ~8.13 in *meica1* (*n* = 56), and ~8.32 in *zep1*^*null*^*-1* (*n* = 146). However, the number of univalents was increased in the double mutant (~1.08 per cell, *n* = 47) relative to *meica1* (~0.08, *n* = 56) and *zep1*^*null*^*-1* (~0.12, *n* = 146), suggesting that complete loss of MEICA1 in the *zep1* background further reduces crossover formation or the maintenance of stable chromosome connections (Figs. 3B and 5E). Together, these results support a model in which ZEP1 promotes accurate meiotic DSB repair by facilitating the recruitment and stable association of the MEICA1–FIGNL1 complex with chromosomes undergoing SC assembly.

**Figure 6:**
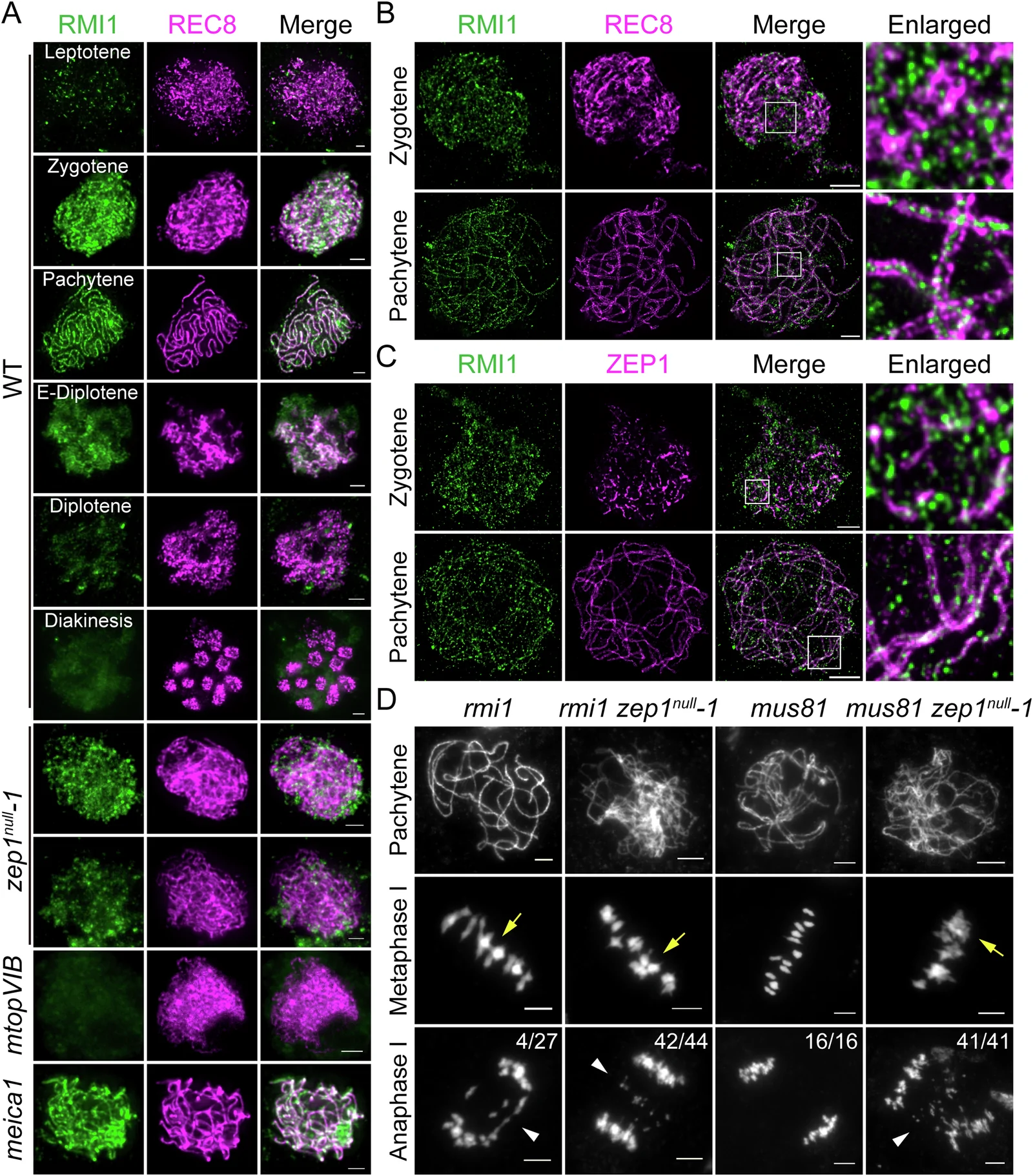
ZEP1 facilitates RMI1 recruitment to synaptonemal complexes during rice male meiosis. (**A**) Immunodetection of RMI1 (green) in wild-type and mutant male meiocytes. Meiotic chromosomes are visualized by axis protein REC8 (magenta). Scale bars, 2 μm. Representative images of RMI1 localization in *zep1*^*null*^*-1* meiocytes were obtained from the same experiment as the images presented in Fig. EV5A, which provides a more comprehensive comparison with additional representative examples. (**B**,** C**) STED (Stimulated emission depletion) super-resolution imaging of either REC8 (B) or ZEP1 (C) (magenta) together with RMI1 (green) in wild-type male meiocytes at zygotene and pachytene stages. Magnified insets of bracketed regions are displayed on the right. Scale bars, 2 μm. (**D**) Chromosome behavior during different meiosis stages in single and double mutant male meiocytes. Meiotic chromosomes are visualized by DAPI staining. Meiotic defects are indicated by yellow arrows (abnormal chromosome connections) and white arrowheads (chromosome fragments or bridges), respectively. Arabic numerals in the panels represent the number of representative images containing chromosome fragments or bridges relative to the total number of observations. A total of *n* = 27 *rmi1*, *n* = 44 *rmi1 zep1*^*null*^*-1*, *n* = 16 *mus81*, and *n* = 41 *mus81 zep1*^*null*^*-1* anaphase I meiocytes were scored from chromosome spreads generated via multiple independent slide preparations using pooled anthers isolated from meiotic florets of independent isogenic plants. Representative images of chromosome behavior in *rmi1* and *rmi1 zep1*^*null*^*-1* meiocytes were obtained from the same experiment as the images presented in Fig. EV5B, which provides a more comprehensive comparison with additional representative examples from different meiotic stages. Scale bars, 5 μm. Source data underlying this figure are provided online.

**Figure EV5:**
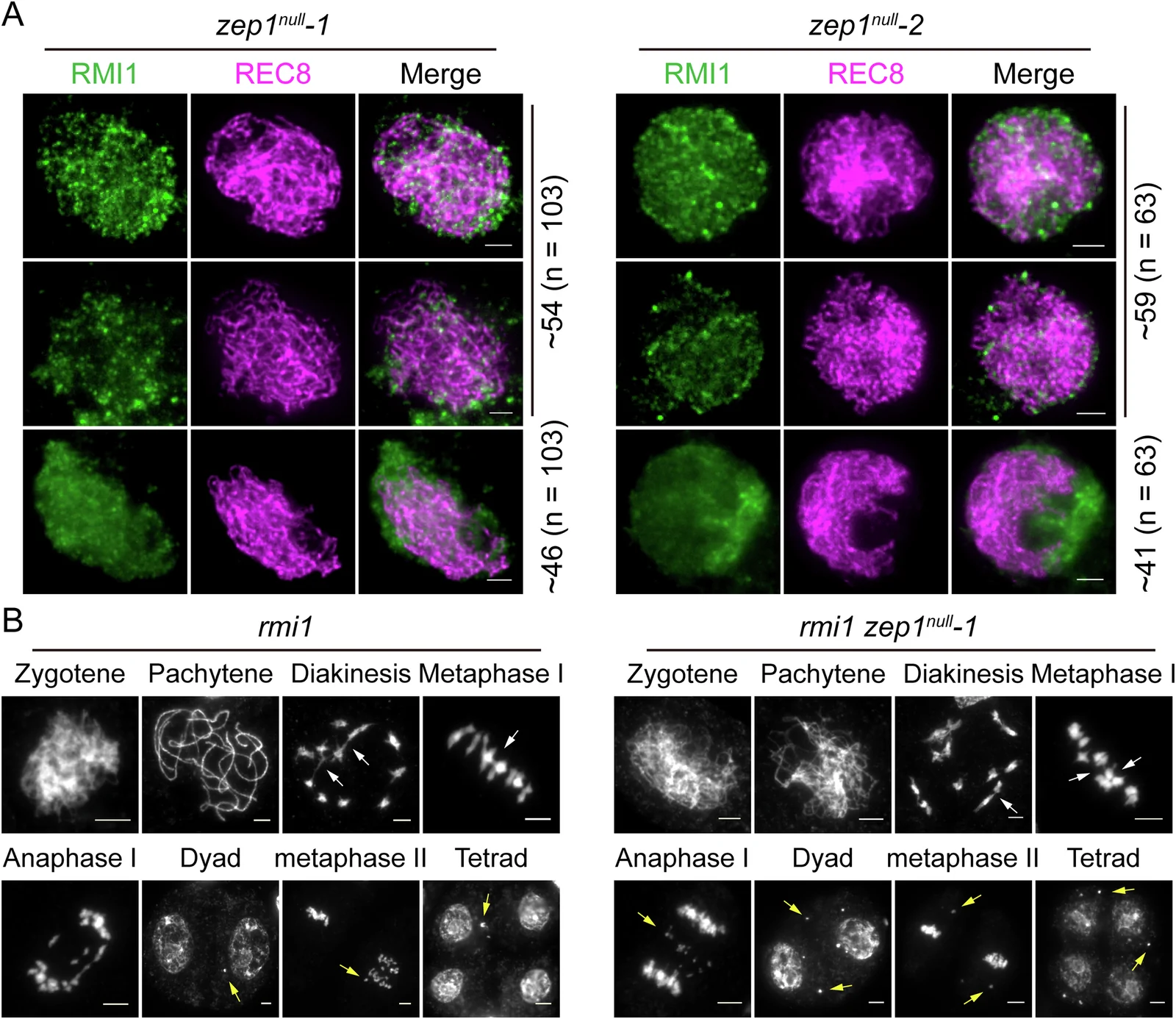
ZEP1 is required for proper chromosomal enrichment of RMI1 during male meiosis. (**A**) Representative images illustrating reduced chromosomal association and ectopic localization of RMI1 protein foci (green) in *zep1* null meiocytes. Meiotic chromosomes are visualized by axis protein REC8 (magenta). Representative images of RMI1 localization in *zep1*^*null*^*-1* meiocytes were obtained from the same experiment as the images shown in Fig. 6A. Additional images are provided here for a more comprehensive comparison. “n” in the panels represents the number of meiocytes displaying the indicated phenotype over the total number of analyzed meiocytes in each group. A total of *n* = 103 *zep1*^*null*^*-1* and *n* = 63 *zep1*^*null*^*-2* prophase I meiocytes were generated via multiple independent slide preparations using pooled anthers isolated from meiotic florets collected from independent plants of the same genotype. Scale bar, 2 μm. (**B**) Chromosome behavior during different stages of *rmi1* and *rmi1 zep1*^*null*^*-1* mutants. Meiocytes were scored from chromosome spreads generated via multiple independent slide preparations using pooled anthers isolated from meiotic florets collected from independent plants of the same genotype. Meiotic chromosomes are visualized by DAPI staining. Abnormal chromosome connections and chromosome bridge, lagging or missing chromosomes are indicated by white and yellow arrows, respectively. Scale bar, 5 μm. Representative images of chromosome behavior in *rmi1* and *rmi1 zep1*^*null*^*-1* meiocytes were obtained from the same experiment as the images shown in Fig. 6D. Additional representative examples from different meiotic stages are provided here for a more comprehensive comparison. Source data underlying this figure are provided online.

In *Arabidopsis*, RMI1 collaborates with TOP3α to process recombination intermediates and maintain meiotic genome stability (Chelysheva et al, 2008; Hartung et al, 2008; Séguéla-Arnaud et al, 2017). We generated a rice RMI1 antibody and validated its specificity by western blot (Appendix Fig. S12A). In wild-type meiocytes, RMI1 first appeared as puncta at leptotene, rapidly increased to a peak at zygotene, and then mainly adopted linear-like signals along meiotic chromosomes, which gradually diminished and disappeared by diakinesis, consistent with sustained activity in intermediate processing. (Fig. 6A). STED (stimulated emission depletion) microscopy revealed that RMI1 was enriched around REC8-labeled chromosome axes and discontinuous ZEP1 stretches, presumably on the chromatin loops at zygotene. By pachytene, RMI1 foci accumulated progressively between paired homologous axes and extensively colocalized with ZEP1 (Fig. 6B,C). In *zep1*^*null*^*-1*, RMI1 signals were either strongly reduced (~54%, *n* = 103) or mislocalized to the nucleoplasm (~46%, *n* = 103). Similar defects were observed in independent CRISPR ZEP1 alleles, whereas ZEP1 localization was not affected in the weak *rmi1* mutant described below (Figs. 6A and EV4A and EV5A), suggesting that ZEP1 is essential for SC-associated enrichment of RMI1. RMI1 loading also required MTOPVIB-dependent DSB formation but was independent of MEICA1 (Fig. 6A). Because *rmi1* null mutations are lethal (Liu et al, 2024), we analyzed a hypomorphic *rmi1* allele (Appendix Figs. S12B,C). *rmi1* meiocytes exhibited severe chromosome entanglements, with ~8.56 multivalents, ~0.16 univalents, and ~3.36 bivalents at metaphase I (*n* = 86), followed by lagging chromosomes or bridges in a subset of anaphase I cells (4/27) and later chromosome missegregation in ~20.2% of cells (17/84) (Figs. 3B and 6D and EV5B), indicating an essential role of rice RMI1 in resolving entanglements. Introducing *rmi1* into the *zep1*^*null*^*-1* did not substantially change metaphase I configurations (~8.26 multivalents, ~0.24 univalents, and ~3.63 bivalents; *n* = 72), while prophase I phenotypes resembled *zep1*^*null*^*-1* (Figs. 3B and 6D and EV5B), consistent with ZEP1 acting upstream to enable RMI1 recruitment, thereby promoting RMI1-dependent resolution of aberrant recombination intermediates and preventing chromosome entanglements. Finally, because MUS81 resolves atypical recombination intermediates (Mu et al, 2023), we tested whether MUS81 becomes particularly important in *zep1*. In *mus81 zep1*^*null*^*-1*, entanglements increased further, with a higher average number of multivalent chromosome pairs at metaphase I (~9.80 in *mus81 zep1*^*null*^*-1* [*n* = 167] versus ~8.32 in *zep1*^*null*^*-1*) without affecting univalent numbers (~0.12 in both genotypes) (Fig. 3B and 6D; Appendix Fig. S12D). These observations reinforce that ZEP1 prevents the accumulation of unregulated recombination intermediates during male meiosis by promoting anti-CO factors recruitment and downstream intermediate processing.

## Discussion

### Rice TF protein directs recombination template choice

Transverse filament (TF) proteins are traditionally viewed as a structural scaffold essential for SC assembly, providing a diffusion medium for CO formation. Nevertheless, existing evidence suggests that TF proteins primarily regulate CO outcomes by modulating the assembly and stability of ZMM foci and controlling HEI10 dynamics along the SC, thereby influencing CO number and distribution. Consistently, TF loss in many species predominantly affects class I COs while leaving DSB repair largely intact. Reduced COs are thought to arise from decreased stability or abundance of CO-designated recombination intermediates, which are subsequently captured by anti-CO factors competing with the ZMM pathway (Börner et al, 2004; de Vries et al, 2005; Pyatnitskaya et al, 2019). Conversely, increased COs may result from enhanced HEI10 diffusion, promoting the formation of additional CO designation sites, as described by the HEI10 coarsening model in *Arabidopsis* (Fozard et al, 2023; Morgan et al, 2021).

In rice, loss of ZEP1 reveals SC functions beyond synapsis and CO formation. ZEP1 exhibits hallmark TF properties—including self-association and interactions with SC components (Fig. 4A,B)—yet *zep1* null mutants exhibit a striking repair-failure signature: persistent non-homologous associations, frequent multivalents, and pervasive acentric chromosome fragments (Figs. 1 and 3). These defects are difficult to reconcile solely with the “more or fewer COs” models and instead point to failures in controlling partner choice and recombination intermediate topology.

Early centromere coupling, which promotes homolog recognition prior to arm pairing (Da Ines and White, 2015; Wang et al, 2024), remains intact in *zep1* mutants. However, ZEP1 is required for the transition to stable homolog engagement, restricting interactions with non-homologous templates and limiting ectopic recombination. Genetic analyses position ZEP1 downstream of strand invasion and reveal that *zep1* defects are strictly DSB-dependent (Figs. 3 and EV2A; Appendix Fig. S9A). Epistasis analysis further shows BRCA2 is epistatic to ZEP1, and blocking DMC1-mediated invasion largely suppresses abnormal associations and fragmentation while allowing DSB repair—albeit inaccurately—via sister chromatids (Fig. 3).

**Figure 7:**
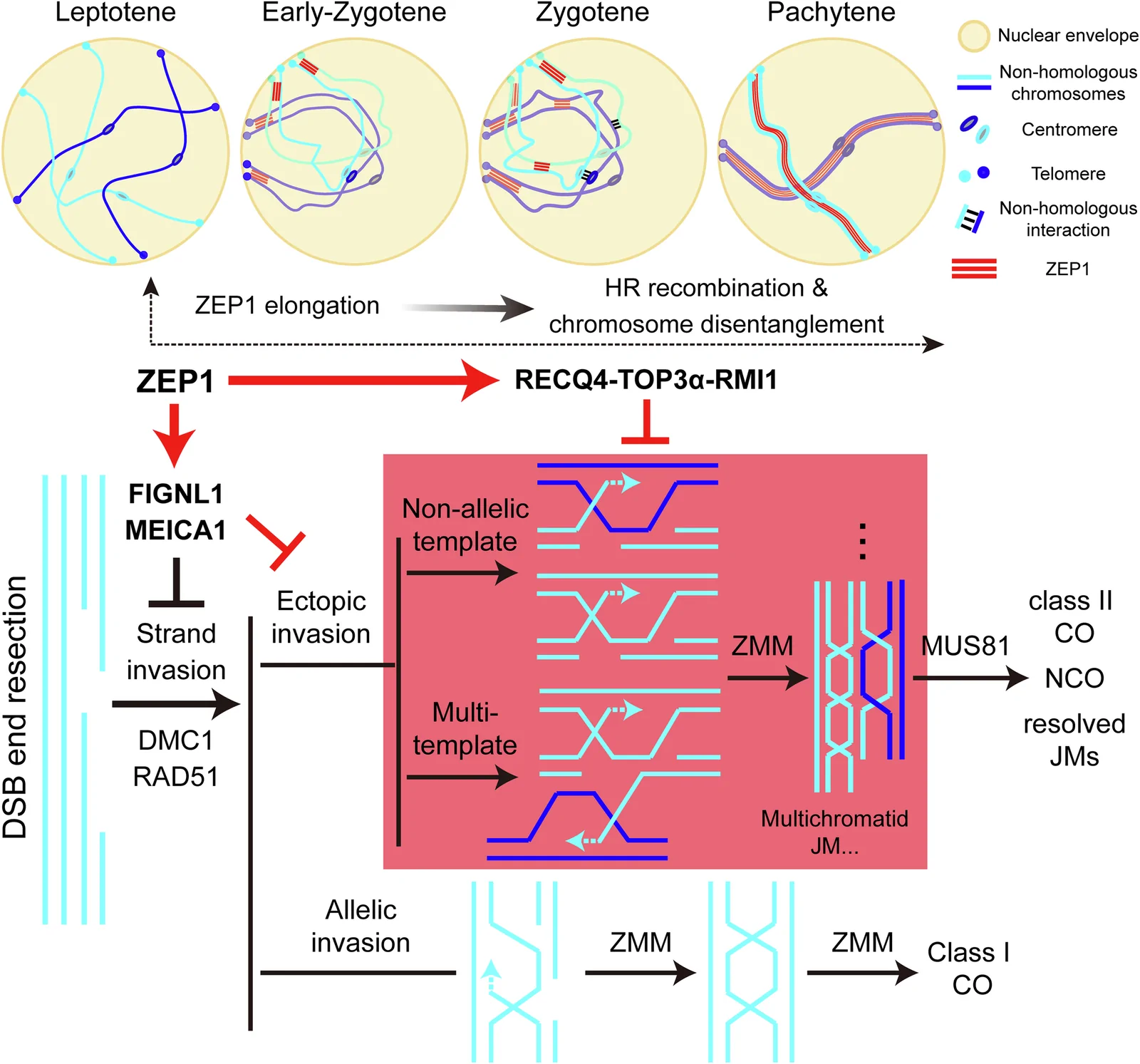
The proposed model of HR-mediated meiotic DSB repair highlights the specific role of ZEP1 in directing the fate of strand-invasion-derived intermediates to enforce homolog bias and coordinate recombination pathway choice during meiosis in rice. Interactions between chromosomal regions sharing sequence similarity among homologs and nonhomologs, particularly in centromeric regions, increase the risk of imperfect template choice during recombination-mediated repair of meiotic DSBs. ZEP1 polymerization drives SC assembly, establishing a physical scaffold that stabilizes homologous chromosome associations while restricting non-homologous interactions. Concurrently, the enrichment of anti-CO proteins by ZEP1 promotes DNA repair within the homologous recombination framework. ZEP1 constrains the progression of MEICA1–FIGNL1—mediated strand-invasion intermediates to suppress ectopic/excessive template invasion concomitantly with SC assembly. ZMM-stabilized intermediates (joint molecules, JMs) are designated for class I COs, whereas the remaining intermediates are processed by the RTR complex and/or MUS81 to generate NCOs and class II COs. In the *zep1* mutant, dysregulated MEICA1–FIGNL1 activity prolongs strand invasion, leading to the formation of aberrant recombination intermediates. These intermediates are competitively engaged by ZMM proteins and can form atypical intermediate structures (e.g., multichromatid joint molecules). Impaired RMI1-dependent RTR complex activity limits their proper resolution, biasing repair toward the MUS81-dependent class II CO pathway and allowing a subset to persist as unresolved intermediates.

Collectively, these findings indicate that *zep1* defects stem from inaccurate template invasion involving both homologous and non-homologous chromosomes, leading to aberrant recombination intermediates that are inefficiently processed. We propose that ZEP1 functions as a structural—molecular coordinator that acts after strand exchange to channel recombination intermediates into productive outcomes, preventing their diversion into persistent, entangling connections. In this context, the SC imposes spatial constraints that limit ectopic interactions and favor homolog-specific recombination (Fig. 7).

### Rice TF protein coordinates spatial constraints and recombination pathways

Building on its role in directing template choice, ZEP1 further integrates spatial constraints with recombination pathway coordination to shape the fate of recombination intermediates. Faithful chromosome segregation requires timely resolution or dissolution of recombination intermediates generated after strand exchange (San-Segundo and Clemente-Blanco, 2020). Anti-CO proteins act at multiple steps to restrain aberrant recombination intermediates and facilitate their processing, often in coordination with MUS81 (Chelysheva et al, 2008; Crismani et al, 2012; Fernandes et al, 2018; Girard et al, 2015; Hartung et al, 2008; Higgins et al, 2011; Jessop and Lichten, 2008; Kaur et al, 2015; Oh et al, 2007; Oh et al, 2008; Tang et al, 2015; Yuan and Chen, 2013); their loss can result in severe chromosome entanglement and segregation defects in rice (Hu et al, 2017; Li et al, 2025; Liu et al, 2024; Yang et al, 2022). Following ZEP1 polymerization along the interaxial region between homologous chromosome axes, MEICA1, FIGNL1, and RMI1 predominantly accumulate on the synaptonemal complex (Figs. 5A,B and 6B,C and EV3), overlapping the ZEP1 domain. Mechanistically, ZEP1 physically interacts with these factors (Fig. 4C,E) and is required for their proper meiotic chromosomal association (Figs. 5C and 6A and EV5A and EV4A; Appendix Fig. S11), supporting a critical role for the rice TF protein ZEP1 in coordinating anti-CO activities within the SC-associated recombination environment after the initiation of homologous synapsis.

Notably, MEICA1, FIGNL1, and RMI1 also display early chromosomal association before ZEP1 polymerization is detectable (Figs. 5A,B and 6C), indicating that at least part of their meiotic function may begin prior to SC assembly. These early association patterns are likely related to the processing of earlier strand-invasion-derived recombination intermediates during or shortly after strand invasion. Pre-synaptic strand invasion events are widely recognized as essential for early homology evaluation, thereby promoting homologous chromosome pairing and triggering the onset of synapsis in recombination-dependent meiotic programs (Gold and Kim, 2026; Zickler and Kleckner, 2015). In this context, our observations suggest that ZEP1 is unlikely to act at the very earliest step of strand invasion itself. Instead, ZEP1-dependent regulation through MEICA1–FIGNL1 and RMI1 most likely occurs concomitantly with SC assembly, when polymerized ZEP1 becomes established between homolog axes and provides a structural and regulatory context for the subsequent processing of strand-invasion-derived recombination intermediates. We therefore propose that, in rice, ZEP1 supports the SC not only as a structural scaffold but also as a post-strand-invasion regulatory platform that helps monitor the fate and maturation of recombination intermediates.

Consistent with impaired control of invasion intermediates, *zep1* mutants show over-accumulation and delayed clearance of DMC1 and RAD51 (Figs. 5D and EV4B–D), indicating that ZEP1 limits aberrant recombination intermediates by promoting MEICA1–FIGNL1-mediated regulation of invasion activity. In parallel, our data reveal a preferential functional association between ZEP1 and the RTR component RMI1. Although the meiotic role of the RTR component TOP3α remains unresolved in rice, RMI1 plays a critical role in rice meiotic DSB repair, as hypomorphic *rmi1* mutants display meiotic defects, whereas null mutants of the RTR component RECQ4 undergo largely normal meiosis despite elevated CO frequency (this study; Li et al, 2025; Liu et al, 2024). These observations support a role for RMI1-dependent RTR function in recombination intermediate processing, with ZEP1 facilitating this process through RMI1 recruitment. Future genetic analyses of *zep1* in combination with *recq4* or *fancm* mutants will help define the distinct contributions of anti-CO regulatory pathways to ZEP1-dependent control of meiotic CO pathway choice.

ZEP1 also influences the dynamics of pro-CO intermediates. Although ZMM recruitment is largely ZEP1-independent, timely removal of ZMM proteins requires ZEP1 (Figs. EV2B,C), suggesting that ZEP1 promotes the turnover and resolution of ZMM-marked pro-CO intermediates. Persistent strand invasion activity and delayed progression of HEI10-marked class I CO intermediates toward maturation in *zep1* mutants further indicate that an expanded pool of recombination intermediates may remain in a ZMM-stabilized pro-CO state (Figs. 2 and 5D and EV4B–D). Genetic interactions further reveal that unregulated intermediates accumulate when stabilization and resolution are imbalanced: ZMM loss partially alleviates *zep1* defects, whereas MUS81 loss exacerbates multivalent formation (Figs. 3A,B and 6D). Together, these findings suggest that the rice TF protein temporally and spatially coordinates anti-CO regulatory pathways with CO-designation and resolution pathways within the SC environment to limit aberrant intermediate formation and promote efficient intermediate resolution.

Overall, our findings support a model in which ZEP1 functions as an active regulatory hub at the transition from strand invasion to recombination intermediate maturation, coupling SC assembly with the regulation of strand-invasion-derived intermediates to promote homolog-biased repair outcomes. Concomitant with SC assembly, polymerized ZEP1 establishes a spatial architecture that limits aberrant interactions with ectopic templates and promotes a recombination-permissive interhomolog configuration. In parallel, ZEP1 facilitates proper SC localization of anti-CO factors and regulates the chromosome-associated dynamics of ZMM proteins, thereby ensuring appropriate processing of recombination intermediates by maintaining a balance between ZMM-mediated pro-CO activities and anti-CO regulation. In this way, ZEP1 links early template choice with downstream pathway selection, integrating chromosome spatial organization with recombination control to safeguard meiotic genome stability (Fig. 7), extending its role beyond simply providing a physical framework for homolog alignment and synapsis.

### Genome context and dosage define SC-dependent recombination outcomes

Multivalents and fragmentation are prevalent in rice TF null mutants but rare in *Arabidopsis thaliana* and largely absent in many non-plant systems (Capilla-Pérez et al, 2021; France et al, 2021). This contrast likely reflects the influence of genome architecture on recombination repair networks. Plant genome architecture likely amplifies the consequences of losing this SC-based control. Large genomes, abundant repetitive DNA, and frequent segmental or whole-genome duplications (Goff et al, 2002; Moore and Purugganan, 2005; Paterson et al, 2003; Tian et al, 2004; Yu et al, 2002) increase the probability that strand invasion will engage near-identical non-allelic templates, elevating the risk of ectopic recombination and complex multichromatid entanglements. In such a genomic landscape, an SC-embedded organizer that biases template choice and constrains aberrant intermediate accumulation becomes critical for maintaining genome integrity—providing a plausible explanation for the pronounced DSB repair-failure phenotype observed in rice TF null mutants and the heightened sensitivity reported in more complex (e.g., polyploid (Zhang et al, 2025)) contexts.

Importantly, aberrant recombination intermediates require MUS81 for resolution, and HEI10-marked intermediates are unchanged or even reduced in *zep1* null mutants, implying ZEP1 normally restricts MUS81-dependent intermediates. This prevents a shift toward noncanonical repair pathways and suppresses class II CO formation. Combined with the established behavior of hypomorphic *zep1* alleles that can elevate class I COs (Wang et al, 2015), these results suggest a dosage-sensitive “window”: partial reduction of ZEP1 activity/abundance relaxes constraints on class I CO formation or redistribution without collapsing recombination intermediate control, whereas complete loss removes an organizing scaffold needed to prevent ectopic interactions and unregulated intermediates, thereby biasing repair toward the class II CO pathway.

Cultivar comparisons further suggest that ZEP1 may be embedded in a tunable recombination network shaped by genetic background and dosage, potentially influencing CO formation. However, the molecular basis of these background-dependent effects remains unresolved. In *japonica* backgrounds, *zep1* null mutants do not increase the final number of bright HEI10 foci but delay their maturation (Fig. 2; Appendix Fig. S8), whereas *indica* and hybrid backgrounds show reduced HEI10 foci and elevated univalents (Figs. 2 and 3B and EV1C). Allelic variation and divergence among meiotic genes (Kong et al, 2008; Lawrence et al, 2019; Liu et al, 2022; Wang and Copenhaver, 2018; Ziolkowski et al, 2017) suggest that ZEP1 loss unmasks cryptic variation, modulating CO designation and progression toward maturation across subspecies. Future studies dissecting how TF proteins integrate with evolving recombination networks during rice divergence remain an important future direction.

In summary, our work redefines the rice TF protein as an active genome-protection factor in meiotic HR. ZEP1 organizes anti-crossover factors within the SC architecture, positioning the SC as a key decision-making platform that ensures template fidelity and balances antagonistic pro- and anti-recombination pathways to govern recombination intermediate fate and safeguard genome integrity. Given the conservation of TF and anti-CO proteins, this TF–anti-CO regulatory module likely represents a broadly conserved mechanism linking SC architecture to recombination intermediate control across sexually reproducing species, particularly those with complex or polyploid genomes.

## Methods

**Table:** Reagents and tools table

Reagent/resource	Reference or source	Identifier or catalog number
**Experimental models**		
*Escherichia coli* DH5α	Lab store	N/A
*Agrobacterium tumefaciens* AH105	weidibio	AC1010S
*Agrobacterium tumefaciens* GV3101	weidibio	AC1001S
*Saccharomyces cerevisiae* AH109	Lab store	N/A
*Nicotiana benthamiana*	Lab store	N/A
*Oryza Sativa*: *japonica*, 9522 wild type	Lab store	N/A
*Oryza Sativa*: *japonica*, *zep1*^*null*^*-1*/*zep1*^*null*^*-2*/*zep1*^*null*^*-3*/*crc1* /*pair2*/ *pair3*/*dmc1*/*msh5*/*meica1*/*fignl1*/*rmi1* single mutants (cv. 9522 background)	This study	N/A
*Oryza Sativa*: *japonica*, *mus81* single mutant (cv. 9522 background)	Lab store	N/A
*Oryza Sativa*: *japonica*, *sds*/*brca2* single mutants (cv. 9522 background)	Fu et al, (2020)	https://doi.org/10.3389/fpls.2020.600820
*Oryza Sativa*: *japonica*, *shoc1* single mutant (cv. 9522 background)	Zhang et al, (2019)	https://doi.org/10.1104/pp.19.00082
*Oryza Sativa*: *japonica*, *sds zep1*^*null*^*-1*/*crc1 zep1*^*null*^*-1*/*pair2 zep1*^*null*^*-1*/*pair3 zep1*^*null*^*-1*/*brca2 zep1*^*null*^*-1*/*dmc1 zep1*^*null*^*-1*/*msh5 zep1*^*null*^*-1*/*shoc1 zep1*^*null*^*-1*/*meica1 zep1*^*null*^*-1*/*rmi1 zep1*^*null*^*-1*/*mus81 zep1*^*null*^*-1* double mutants (cv. 9522 background)	This study	N/A
*Oryza Sativa*: *japonica*, Nipponbare wild type	Lab store	N/A
*Oryza Sativa*: *japonica*, *zep1*^Nipponbare^ single mutant (cv. Nipponbare background)	This study	N/A
*Oryza Sativa*: *japonica*, *zep1-3* single mutant (cv. Nipponbare background)	Wang et al, (2015)	https://doi.org/10.1016/j.molp.2015.04.011
*Oryza Sativa*: *Indica*, Kasalath wild type	This study	N/A
*Oryza Sativa*: *Indica*, *zep1*^Kasalath^ single mutant (cv. Kasalath background)	This study	N/A
*Oryza Sativa*: *Indica*, Minghui 63 wild type	This study	N/A
*Oryza Sativa*: *Indica*, *zep1*^Minghui 63^ single mutant (cv. Minghui 63 background)	This study	N/A
*Oryza Sativa*: *Indica*, Zhenshan97 wild type	This study	N/A
*Oryza Sativa*: *Indica*, *zep1*^Zhenshan97^ single mutant (cv. Zhenshan97 background)	This study	N/A
Oryza Sativa: indica-japonica hybrid, Chunyou 84 wild type	Liu et al, (2021)	https://doi.org/10.3389/fpls.2021.757152
*Oryza Sativa*: indica-japonica hybrid, *zep1*^Chunyou 84^ single mutant (cv. Chunyou 84 background)	This study	N/A
**Recombinant DNA**		
Plasmid: pGEX-4T-1-MEICA1/-FIGNL1/-RMI1	This study	N/A
Plasmid: pGBKT7-ZEP1	This study	N/A
Plasmid: pGADT7-ZEP1/-RAD17/-RAD9/-RAD1/-HUS1/-GEN1/-MUS81/-PAIR2/-PAIR3/-CRC1/-MEICA1/-FIGNL1/-RMI1/-TOP3α/-RECQ4/-FANCM	This study	N/A
Plasmid: pGADT7-MSH4/-MSH5/-SHOC1/-PTD/-HEI10/-ZIP4/-MER3	Zhang et al, (2019)	https://doi.org/10.1104/pp.19.00082
Plasmid: pGADT7-DMC1A/-DMC1B/-RAD51A1/-RAD51A2	Fu et al, (2020)	https://doi.org/10.3389/fpls.2020.600820
Plasmid: pGADT7-BRCA2/-RAD51C/-RAD51D/-XRCC3	Lab store	N/A
Plasmid: pRGEB32-CRISPR-ZEP1-2/-ZEP1-3 /-CRC1/-DMC1A1B/-PAIR2/-PAIR3/-MEICA1/-FIGNL1/-RMI1-1/-RMI1-2	This study	N/A
Plasmid: pBin-sgR-Cas9-CRISPR-MSH5	This study	N/A
Plasmid: pBin-sgR-Cas9-CRISPR-MUS81	Lab store	N/A
Plasmid: pXY104-ZEP1-cYFP	This study	N/A
Plasmid: pXY106-nYFP-MEICA1/-FIGNL1/-RMI1/-RECQ4/-TOP3α-/MSH4/-MSH5/-MER3/-ZIP4/-RAD17/-RAD9/-RAD1/-HUS1/-GEN1/-MUS81/-DMC1A/-DMC1B/-RAD51A1/-RAD51A2/-RAD51C/-RAD51D/-BRCA2/-XRCC3	This study	N/A
Plasmid: pXY106-nYFP-PTD/-SHOC1/-HEI10	Zhang et al, (2019)	https://doi.org/10.1104/pp.19.00082
Plasmid: pREUR-ZEP1-HA	This study	N/A
Plasmid: pREUR-MEICA1-YFP	This study	N/A
Plasmid: pREUR-FIGNL1-YFP	This study	N/A
Plasmid: pREUR-RMI1-YFP	This study	N/A
**Antibodies**		
Mouse polyclonal anti-REC8	He et al, (2016)	https://doi.org/10.1105/tpc.16.00108
Rabbit polyclonal anti-REC8	Wang et al, (2017)	https://doi.org/10.1104/pp.16.01726
Mouse polyclonal anti-ZEP1	Wang et al, (2017)	https://doi.org/10.1104/pp.16.01726
Rabbit polyclonal anti-ZEP1	He et al, (2016)	https://doi.org/10.1105/tpc.16.00108
Rabbit polyclonal anti-DMC1	Wang et al, (2017)	https://doi.org/10.1104/pp.16.01726
Rabbit polyclonal anti-RAD51	Fu et al, (2020)	https://doi.org/10.3389/fpls.2020.600820
Rabbit polyclonal anti-RAD51C	He et al, (2016)	https://doi.org/10.1105/tpc.16.00108
Rabbit polyclonal anti-HEI10	Wang et al, (2017)	https://doi.org/10.1104/pp.16.01726
Rabbit polyclonal anti-ZIP4	Zhang et al, (2019)	https://doi.org/10.1104/pp.19.00082
Rabbit polyclonal anti-MEICA1	This study	N/A
Rabbit polyclonal anti-FIGNL1	This study	N/A
Rabbit polyclonal anti-RMI1	This study	N/A
Goat anti-rabbit IgG (Alexa Fluor 555)	Thermo	Cat# A27039; RRID: AB_2536100
Goat anti-mouse IgG (DyLight 488)	Abbkine	Cat# A23210
Goat anti-rabbit IgG (DyLight 488)	Abbkine	Cat# A23220
Goat anti-mouse IgG (Alexa Fluor 555)	Thermo	Cat# A28180; RRID: AB_2536164
Goat anti-rabbit IgG, STAR ORANGE	abberior	Cat# STORANGE-1002; RRID: AB_3068622
Goat anti-Mouse IgG, STAR RED	abberior	Cat# STRED-1001; RRID: AB_3068620
Monoclonal anti-Tubulin	Beyotime	Cat# AT819
Monoclonal anti-GFP	Proteintech	Catalog Number: 66002-1-Ig
Monoclonal anti-HA	Abmart	Catalog Number: M20003
Goat anti-mouse IgG-horseradish peroxidase (HRP) conjugate	Abmart	Catalog Number: M21001
Goat anti-rabbit IgG- horseradish peroxidase (HRP) conjugate	Abmart	Catalog Number: M21002
**Oligonucleotides and other sequence-based reagents**		
PCR primers	This study	Appendix Table S1
FISH Probes	Zhang et al, (2019)	Appendix Table S1
**Chemicals, enzymes and other reagents**		
TRNzol Universal	TIANGEN	Cat# DP424
Blocking reagent	Roche	Cat# 11096176001
Sodium citrate buffer	Sigma	Cat# C9999
Goat serum	Boster	Cat# AR0009
4’,6-diamidino-2-phenylindole	Vector Laboratories	Cat# 0215757401
Protease Inhibitor Cocktail, EDTA-free	MedChemExpress	Cat# HY-K0010
DO Supplement -Leu/-Trp	Clontech	Cat# 630417
DO Supplement -His/-Leu/-Trp/-Ade	Clontech	Cat# 630428
FastKing RT kit (with gDNase)	TIANGEN	Cat# KR116-02
In-Fusion Snap Assembly Master Mix	TaKaRa	Cat# 638947
ClonExpress II One Step Cloning Kit	Vazyme	Cat# C112
Plant Nuclear Protein Extraction Kit	Beijing Baiao Leibo	Cat# HR8053
Anti-GFP Nanobody Magarose Beads	AlpaLifeBio	Cat# KTSM1334
**Software**		
GraphPad prism 8	GraphPad	https://www.graphpad.com/scientific-software/prism/
IBM SPSS Statistics	IBM Corporation	https://www.ibm.com/products/spss-statistics
NIS-Elements Advanced Research	Nikon	https://www.microscope.healthcare.nikon.com
Huygens software	Scientific Volume Imaging	https://svi.nl/Huygens-Software
Adobe Illustrator 2024	Adobe	https://www.adobe.com/products/illustrator.html
Photoshop CS6	Adobe	https://www.adobe.com/products/photoshop.html
PowerPoint 2021	Microsoft	https://www.microsoft.com/
NomisProX-C	Nexcope	https://www.yxopt.com
ImageJ and Fiji	NIH	https://imagej.net/software/fiji/
**Other**		
ChemiDoc™ MP Imaging System	Bio-Rad	
Eclipse Ni-E microscope	Nikon	
Abberior STEDYCON	Abberior Instruments GmbH	
TCS SP5 confocal laser scanning microscope	Leica	
NCF100 confocal laser scanning microscopes	NOVEL	

### Plant materials and growth conditions

The *zep1*^*null*^*-1* mutant was identified from our ^60^Co γ-irradiated mutant library generated in *O. sativa* ssp. *Japonica* cv. 9522 rice. To identify the mutated gene, map-based cloning was performed with an F2 population derived from a cross between the mutant (*O. sativa* ssp. *Japonica* cv. 9522) and Guang Lu Ai4 (*indica*) wild-type plants. The locus was mapped between two Insertion-Deletion (InDel) molecular markers, SNP4 and HY002, on the long arm of chromosome 4, with a genetic distance of 66.4 and 70.1 centimorgans, respectively. We initially identified candidate mutation sites via bioinformatic analysis of high-throughput sequencing data, followed by PCR-based validation using primers ZEP1-JY-F (forward) and ZEP1-JY-R (reverse) (listed in Appendix Table S1). The hypomorphic allele *zep1-3* was previously described (Wang et al, 2015; Wang et al, 2010). Single mutants (*zep1*, *crc1*, *pair2*, *pair3*, *dmc1*, *zep1*^*null*^*-2*, *zep1*^*null*^*-3*, *meica1*, *fignl1*, *rmi1*) and their corresponding double mutants with *zep1*^*null*^*-1* were generated using CRISPR-Cas9-mediated gene knockout as previously described (Xie et al, 2015). Additional CRISPR-generated knockout mutants (*msh5* and *mus81*) were produced following the previously reported method (Zhang et al, 2014). The *msh5 zep1*^*null*^*-1* and *mus81 zep1*^*null*^*-1* double mutants were obtained by crossing heterozygous *msh5* plants or homozygous *mus81* plants, respectively, with heterozygous *zep1*^*null*^*-1* plants. The primers for constructing the sgRNA vectors and genotyping the transgenic plants are listed in Appendix Table S1. The sequencing results of the knock-out mutant are shown in Appendix Fig. S13. Other mutants (*sds*, *brca2*, *shoc1*) have been described in our previous studies (Fu et al, 2020; Zhang et al, 2019), and related double mutants with *zep1*^*null*^*-1* were generated by crossing between heterozygous plants. Rice plants were grown in the paddy field (in summer) or growth chambers (in winter) of Shanghai Jiao Tong University. For tobacco (*N. benthamiana*) planting, *Nicotiana benthamiana* plants were grown in a greenhouse under 16 h/8 h (light/dark) photoperiod at 23/19 °C (day/night). Leaves of 4–5-week-old tobacco plants were used for agroinfiltration.

### Cytological analysis

Rice pollen grains were stained with 1% (w/v) I_2_-KI solution to detect the viability. For the observation of mature embryo sacs, flowering rice spikelets were collected and fixed in FAA solution (50:5:10:35 (v/v) ethanol: glacial acetic acid: formaldehyde: water), and processed as previously described (Wang et al, 2016).

Fresh rice panicles at the meiosis stage were harvested from independent plants of the same genotype and fixed in Carnoy’s solution (3:1 (v/v) ethanol: glacial acetic), then stored at 4 °C for short-term preservation and at −20 °C for long-term storage. For DAPI and FISH assays, post-fixation procedures were carried out as previously described (He et al, 2016). The FISH probes (listed in Appendix Table S1) used in this study have been described in our previous study (Zhang et al, 2019).

For immunostaining analysis, meiotic florets were collected from multiple plants, pooled, and fixed in 4% (w/v) paraformaldehyde at room temperature for 30–40 min. Following three washes with 1× phosphate-buffered saline (PBS), fixed anthers were dissected and squashed on positively charged Superfrost™ Plus slides (Epredia, REF 4951PLUS-001E) for meiocyte spreading. Prepared chromosome spreads were stored in liquid nitrogen for long-term storage before antibody incubation. For immunolocalization assay, different primary antibody combinations were diluted (1:50–1:300, depending on the antibody titer) in TNB buffer (0.1 M Tris-HCl, pH 7.5, 0.15 M NaCl, and 0.5% [w/v] blocking reagent) or goat serum (Boster Biological Technology, Wuhan, China.), and incubated 3–6 h at 37 °C in a moisture chamber. After three rounds of washing in 1×PBS buffer with 0.1% (v/v) Tween-20, goat anti-mouse/rabbit fluorescent secondary antibodies (diluted by 1:800 in TNB buffer or goat serum) were added and incubated at 37 °C for 50 min in the dark. The other processing steps were conducted as previously described (Zhang et al, 2019). Immunodetection of HEI10 was performed on Carnoy’s-fixed chromosome spreads as described with minor modifications (Chelysheva et al, 2010). Fixed anthers were dissected and squashed on positively charged Superfrost™ Plus slides in 45% (v/v) acetic acid solution. After being frozen in liquid nitrogen, the slides were microwaved in 10 mM sodium citrate buffer (pH 6.0) with temperature control at 97 °C for 2–3 min at 850 W and immediately transferred to 1× PBS buffer with 1% (v/v) Triton X-100 at 4 °C for 30 min. The samples were incubated with goat serum (Boster Biological Technology, Wuhan, China) for 40 min at 37 °C to block non-specific binding. Subsequently, the primary antibodies against HEI10 (rabbit, diluted by 1:300 in goat serum) and REC8 (mouse, diluted by 1:200 in goat serum) were added and incubated overnight (~20 h) at 4 °C in a moisture chamber. After three rounds of washing in 1×PBS buffer with 0.1% (v/v) Tween-20, the samples were blocked twice with goat serum for 40 min at 37 °C, then goat anti-Rabbit IgG (heavy and light chain) Superclonal™ secondary antibody (Alexa Fluor 555; Thermo; 1:800) and DyLight 488-conjugated goat anti-mouse secondary antibody (Abbkine; 1:800) diluted in goat serum were added, and incubated at 37 °C for 50 min in the dark. After counterstaining with DAPI (Vector Laboratories), images were captured with an Eclipse Ni-E microscope (Nikon), and analyzed using NIS-Elements Advanced Research software. Counting of fluorescent foci was performed using image deconvolution with the function “Mexican Hat” as previously described (Zhang et al, 2019).

For super-resolution images capturing, paraformaldehyde-fixed meiocytes were first blocked with goat serum (Boster Biological Technology, Wuhan, China) for 40 min at 37 °C, followed by subsequent processing steps as described above for the immunolocalization assay. Primary polyclonal antibodies against RMI1 (rabbit, diluted by 1:50 in goat serum), MEICA1 (rabbit, diluted by 1:150 in goat serum), FIGNL1 (rabbit, diluted by 1:150 in goat serum), ZEP1 (mouse, diluted by 1:150 in goat serum) and REC8 (mouse, diluted by 1:150 in goat serum) were incubated with the samples at 37 °C for ~5 h in a moisture chamber. STED microscopy was performed using 561-nm (for STAR Orange [diluted by 1:100 in goat serum]) and 640-nm (for STAR Red [diluted by 1:100 in goat serum]) excitation lasers coupled with a 775-nm STED depletion laser. Super-resolution images were acquired using Abberior STEDYCON (Abberior Instruments GmbH, Göttingen, Germany) mounted on a motorized inverted microscope IX83 (Olympus UPlanXAPO 100x, NA1.45, Tokyo, Japan). Super-resolution Image processing, primarily deconvolution, was conducted with LIGHTBOX or Huygens software.

### Antibody production and Western blot assay

Coding sequences of 177 (residues 22–198) and 199 (residues 206–404) amino acid peptides of OsFIGNL1 and OsRMI1 were amplified, and cloned into pGEX-4T-1 expression vectors. The coding sequence corresponding to aa 895–1045 of OsMEICA1 was also amplified and cloned, as previously described (Hu et al, 2017). Rabbit polyclonal antibodies against OsMEICA1, OsFIGNL1, and OsRMI1 were prepared by ABclonal (Wuhan, China), including recombinant protein expression, immunization, and antibody production. Rabbit polyclonal antibodies against the rice meiotic proteins ZEP1, RAD51C, DMC1, RAD51, ZIP4, and HEI10, as well as a mouse polyclonal antibody against rice REC8 and ZEP1, were generated in our previous reports (Fu et al, 2016; Fu et al, 2020; He et al, 2016; Wang et al, 2017; Zhang et al, 2019). An anti-ZEP1 polyclonal antibody (amino acids 426–612) raised in mouse was also used for immunoblotting following a previous report (Wang et al, 2010). All primers used are listed in Appendix Table S1.

Rice meiotic flowers were ground to a fine powder in a pre-chilled mortar with liquid nitrogen, and total nuclear proteins were extracted using a Plant Nuclear Protein Extraction Kit (Beijing Baiao Leibo Technology Co., Ltd) following the manufacturer’s instructions. Proteins were separated on 4–12% Super-PAGE™ Precast Gel (Bis-Tris, Stain-Free; Epizyme) and transferred to nitrocellulose membrane by wet transfer. Membranes were blocked in TBST (0.05% Tween-20) containing 5% dried skimmed milk at room temperature on a horizontal shaker (70 rpm) for 40 min, and incubated overnight at 4 °C with primary antibodies: mouse polyclonal anti-ZEP1, rabbit polyclonal anti-RMI1 (diluted 1:1000) and monoclonal anti-tubulin (diluted 1:4000). After three times washing with 1× Tris-buffered saline (TBS) with 0.05% (v/v) Tween-20, membranes were incubated with HRP-conjugated goat anti-mouse or goat anti-rabbit secondary antibodies for 1–2 h at room temperature. Protein signals were detected using a ChemiDoc™ MP Imaging System (Bio-Rad).

### Y2H, BiFC, and Co-IP assays

The Y2H assays were performed according to the manufacturer’s user manual (Clontech). The full-length ZEP1 coding sequence was cloned into pGBKT7, encoding the Gal4 binding domain (Gal4BD). Full-length coding sequences of potential interacting proteins were cloned into pGADT7 encoding the Gal4 activation domain (Gal4AD), and co-transformed with Gal4BD-ZEP1 expression construct into competent *Saccharomyces cerevisiae* AH109 cells. Transformants were selected on synthetic dropout (SD/-Leu/-Trp) nutrient media, and positive clones were transferred and grown on a synthetic dropout (SD/-Leu/-Trp/-His/-Ade) nutrient medium with X-α-gal to test for interaction. Well-grown transformants exhibiting blue coloration on quadruple dropout plates containing X-α-gal served as two criteria to confirm interactions between paired proteins in yeast two-hybrid experiments.

For the BiFC assay, the full-length ZEP1 coding sequence was cloned into the PXY104 vector carrying the C-terminal YFP fragment (cYFP), while coding sequences for potential interacting proteins were cloned into PXY106 vector containing the N-terminal YFP fragment (nYFP). Vectors were individually transformed into competent *Agrobacterium tumefaciens* GV3101 cells, grown to OD600 0.8–1.0, and resuspended in induction medium (10 mM MES, 10 mM MgCl_2_, 200-μm acetosyringone) to OD600 0.4–0.6. Cell suspensions expressing ZEP1 and potential interactor were mixed at a 1:1 ratio, incubated in darkness for 2–4 h and then infiltrated into young *N. benthamiana* leaves. After 24 h incubation in the dark, infected leaves were examined using either a TCS SP5 confocal laser scanning microscope (Leica) or a NEXCOPE NCF1000 laser scanning confocal microscope (Novel Optics, China) for spaced observation. The presence of specific nuclear fluorescent signals verifies interaction between the two proteins under investigation.

For the Co-IP assay, *35S::ZEP1-HA* and *35S::MEICA1-YFP*, *35S:: FIGNL1-YFP*, *35S:: RMI1-YFP*, or *35S::YFP* (negative control) were co-expressed in tobacco leaves as described above. *N. benthamiana* leaf tissues were harvested at ~40 h post agroinfiltration for Co-IP analysis. Protein extraction, beads incubation was performed mainly as previously described (Zhang et al, 2023). Immunoblot detection of Co-IP products was conducted following the Western blot procedure outlined above. For sequential immunoblot reprobing, each nitrocellulose membrane was first immunoblotted with anti-HA antibody and imaged. Membranes were subsequently stripped in 0.2 M NaOH stripping solution for 20–30 min with shaking at 70 rpm at room temperature to elute bound primary and secondary antibodies. After brief rinsing with ultrapure water to neutralize residual alkali, membranes were washed three times for 5 min each in TBST containing 0.05% Tween-20, re-blocked, and reprobed with anti-GFP antibody for secondary detection. All primers used are listed in Appendix Table S1.

### Figure preparation

Immunofluorescence images were acquired with a 100× oil immersion objective. Multicolor fluorescent images were generally acquired sequentially along the Z-axis from the upper to the lower layers of the sample, with a step size of 0.2–0.5 µm. Images were preprocessed and cropped mainly using NIS-Elements Advanced Research or Fiji software. All images in this study were processed with contrast adjustment, and/or deconvolution, and/or maximum-intensity projection (for z-stacks with multichannel signals), unless indicated otherwise. The gene structure diagrams were drawn using IBS software (version 6.0; http://ibs.biocuckoo.org/). Figure alignments and illustrations were done in Adobe Illustrator 2024, Photoshop CS6 or PowerPoint 2021.

### Quantification and statistical analysis

GraphPad Prism 8 (version 8.0.2) was used to generate scatter plots showing individual data points and to perform an unpaired two-tailed Student’s *t*-test for comparisons between two groups. One-way ANOVA was performed using IBM SPSS Statistics (version 20.0) to assess differences among multiple groups. Detailed descriptions of quantification and statistical analyses are provided in the figures, figure legends, or Methods section. Sample sizes and significance levels are indicated in the corresponding figure legends.

## Supplementary information

Appendix

Peer Review File

Expanded View Figures

## Data Availability

Raw data supporting this study have been deposited in the BioImage Archive under accession number S-BIAD3828. https://www.ebi.ac.uk/biostudies/bioimages/studies/S-BIAD3828. The source data of this paper are collected in the following database record: biostudies:S-SCDT-10_1038-S44318-026-00910-0.
