# Supplementary material for: ZEP1 orchestrates template choice and crossover pathways to ensure meiotic genome integrity in rice
Source: EMBO J. 2026 Sep 17;45(20):7163–90. doi: 10.1038/s44318-026-00910-0 (PMC13672304; doi:10.1038/s44318-026-00910-0)
Supplement: Supplementary file 1 [file 44318_2026_910_MOESM1_ESM.pdf]

Appendix for

ZEP1 orchestrates template choice and crossover pathways to ensure meiotic genome integrity in rice

Table of Contents

Appendix Figure S1. Map-based cloning and schematic diagram of the rice *ZEP1* gene......Page 2
Appendix Figure S2. The expression of ZEP1 protein in meiocytes of wild type, *zep1*^{null} mutants and *ZEP1* Tos17 insertion mutant (*zep1-3*)..Page 3
Appendix Figure S3. Morphological and cytological analysis of wild-type and *zep1*^{null}*-1* plants...Page 4
Appendix Figure S4. Generation of *zep1*^{null} CRISPR lines (*zep1*^{null}*-2*, *zep1*^{null}*3*)...............Page 5
Appendix Figure S5. *zep1*^{null} CRISPR lines exhibit defects in male fertility..........................Page 6
Appendix Figure S6. The mature HEI10 protein foci number does not change in *zep1*^{null}*-1* male meiocytes at later prophase I...Page 7
Appendix Figure S7. *zep1* null lines of different rice varieties are all male sterile.................Page 8
Appendix Figure S8. The HEI10 protein foci dynamics in wild-type and *zep1* null male meiocytes of different rice varieties...Page 9
Appendix Figure S9. Chromosome behavior in meiosis-related mutants and RAD51C protein localization in *zep1* male meiosis...Page 10
Appendix Figure S10. Interaction of ZEP1 protein with rice proteins known to be involved in meiosis process...Page 11
Appendix Figure S11. Immunodetection of MEICA1 and FIGNL1 proteins in wild-type and mutant male meiocytes...Page 12
Appendix Figure S12. The phenotype of *rmi1* hypomorphic allele and meiotic chromosome behavior of *mus81 zep1*^{null}*-1* mutants...Page 13
Appendix Figure S13. WT target sequences used for genome-editing in meiosis-related genes...Page 14
Appendix Table S1. Primers used in this study...Page 15

Appendix Figures

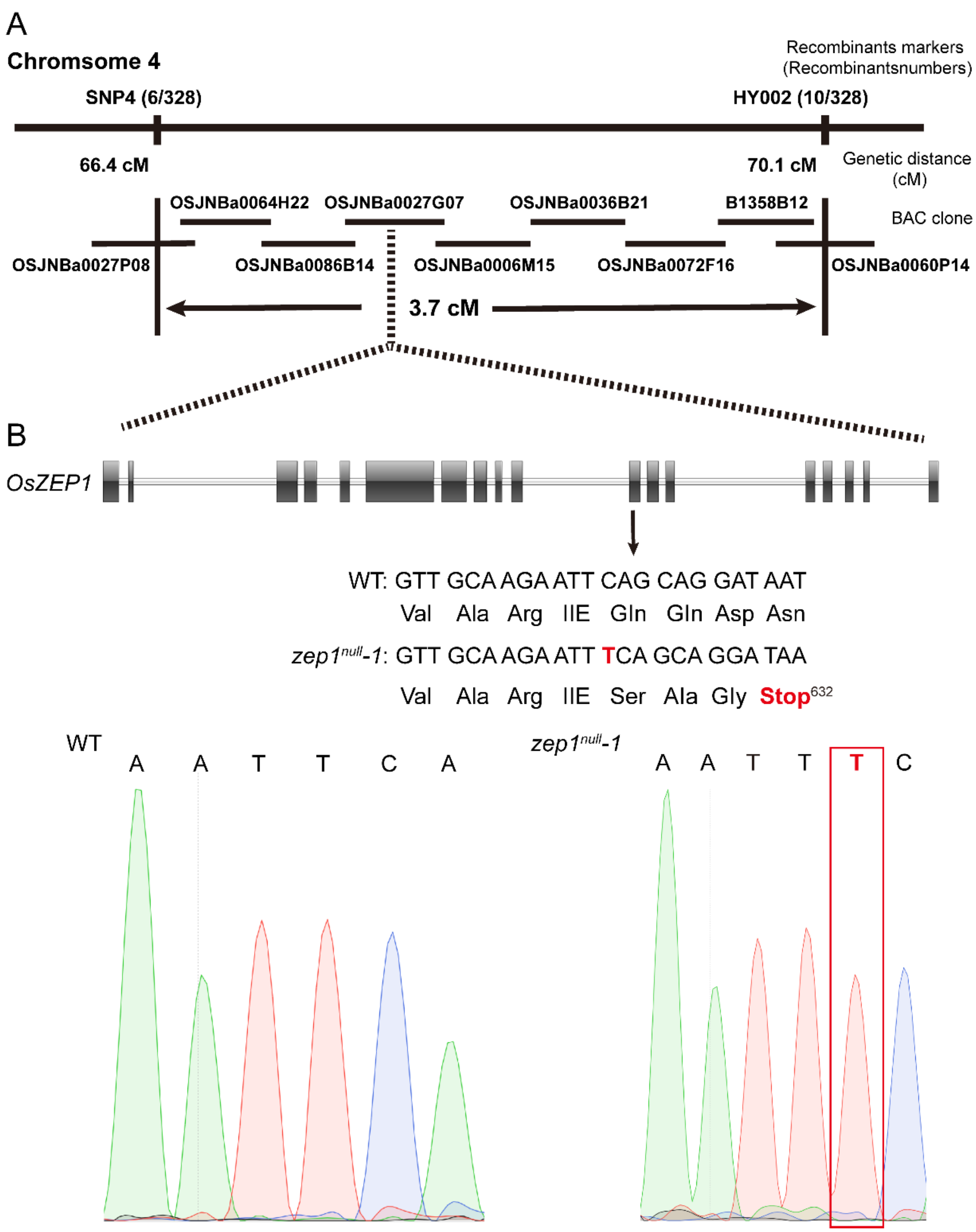

Appendix Figure S1. Map-based cloning and schematic diagram of the rice *ZEP1* gene. **(A)** Map-based cloning of the *OsZEP1* gene. The names and positions of the molecular markers are indicated. OsZEP1 locates within the OSJNBa0027G07 BAC (bacterial artificial chromosome) clone between markers SNP4 and HY002. **(B)** *OsZEP1* comprises comprising 18 exons (black blocks) and 17 introns (white lines). A 1 bp insertion (red; boxed in sequencing results) in the 11th exon causes a frameshift and premature translational termination at amino acid 632 in *zep1*^{9522} mutant.

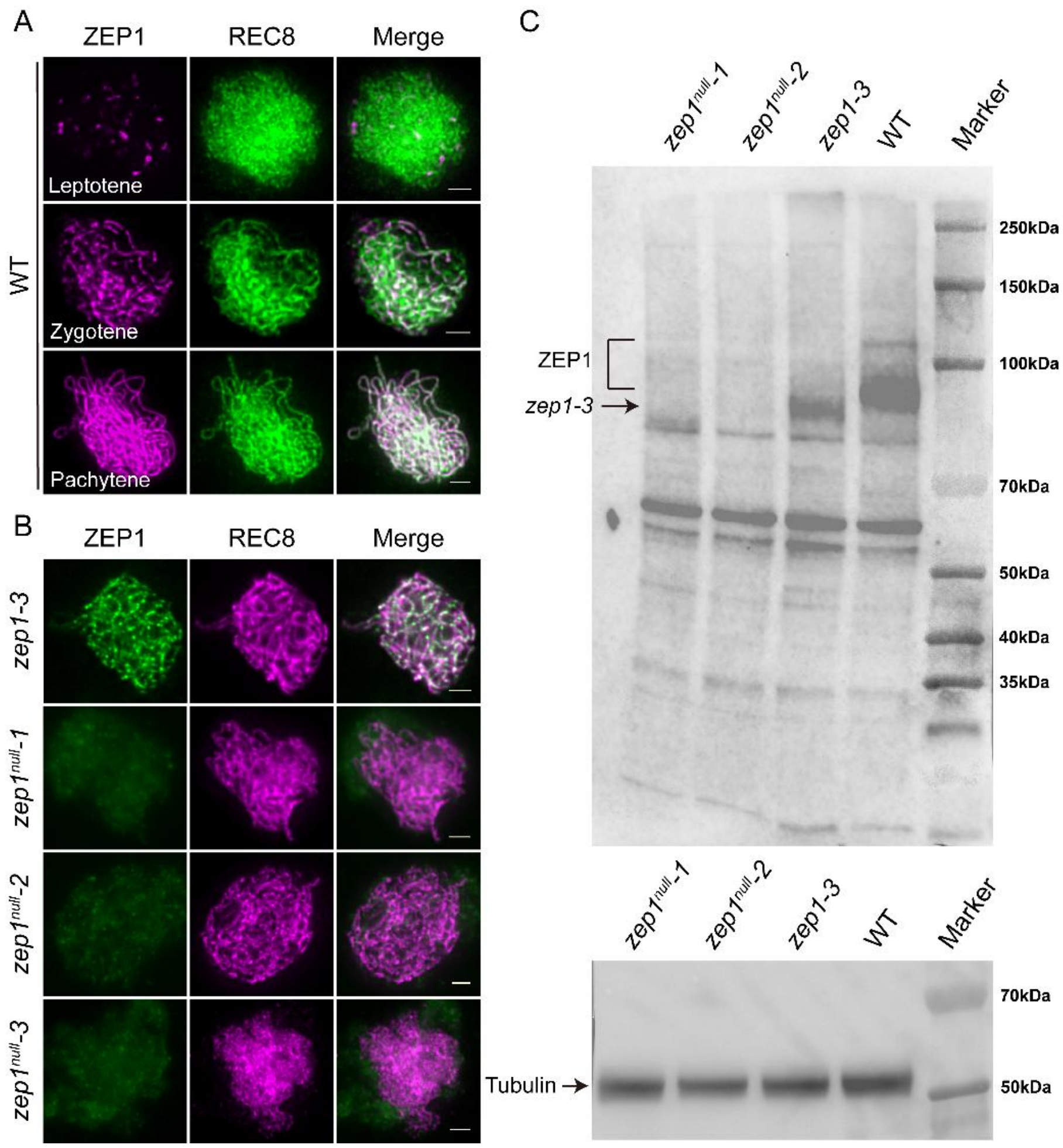

Appendix Figure S2. The expression of ZEP1 protein in meiocytes of wild type, *zep1*^{null} mutants and *ZEP1* Tos17 insertion mutant (*zep1-3*).

(A–B) During normal pachytene progression, wild-type male meiocytes gradually form continuous chromosome-spanning linear ZEP1 (magenta) signals along meiotic chromosomes (A). In contrast, *zep1-3* male meiocytes exhibit discrete ZEP1 protein foci (green) on meiotic chromosomes, while ZEP1 signals are undetectable in *zep1* null male meiocytes at the pachytene-like stage (B). Meiotic chromosomes are visualized by axis protein REC8. Meiocytes were scored from chromosome spreads generated using pooled anthers dissected from meiotic florets collected from independent plants of the same genotype. Scale bar, 2 μm. **(C)** Immunoblot detection of ZEP1 protein (Top) and Tubulin (bottom) in rice meiotic flowers of wild-type plants, *zep1*^{null} mutants and *ZEP1* Tos17 insertion mutant (*zep1-3*). Meiotic proteins were extracted from pooled meiotic florets collected from independent plants of the same genotype. Multiple independent Western blot assays confirmed consistent phenotypic observations. Tubulin was used as an internal control.

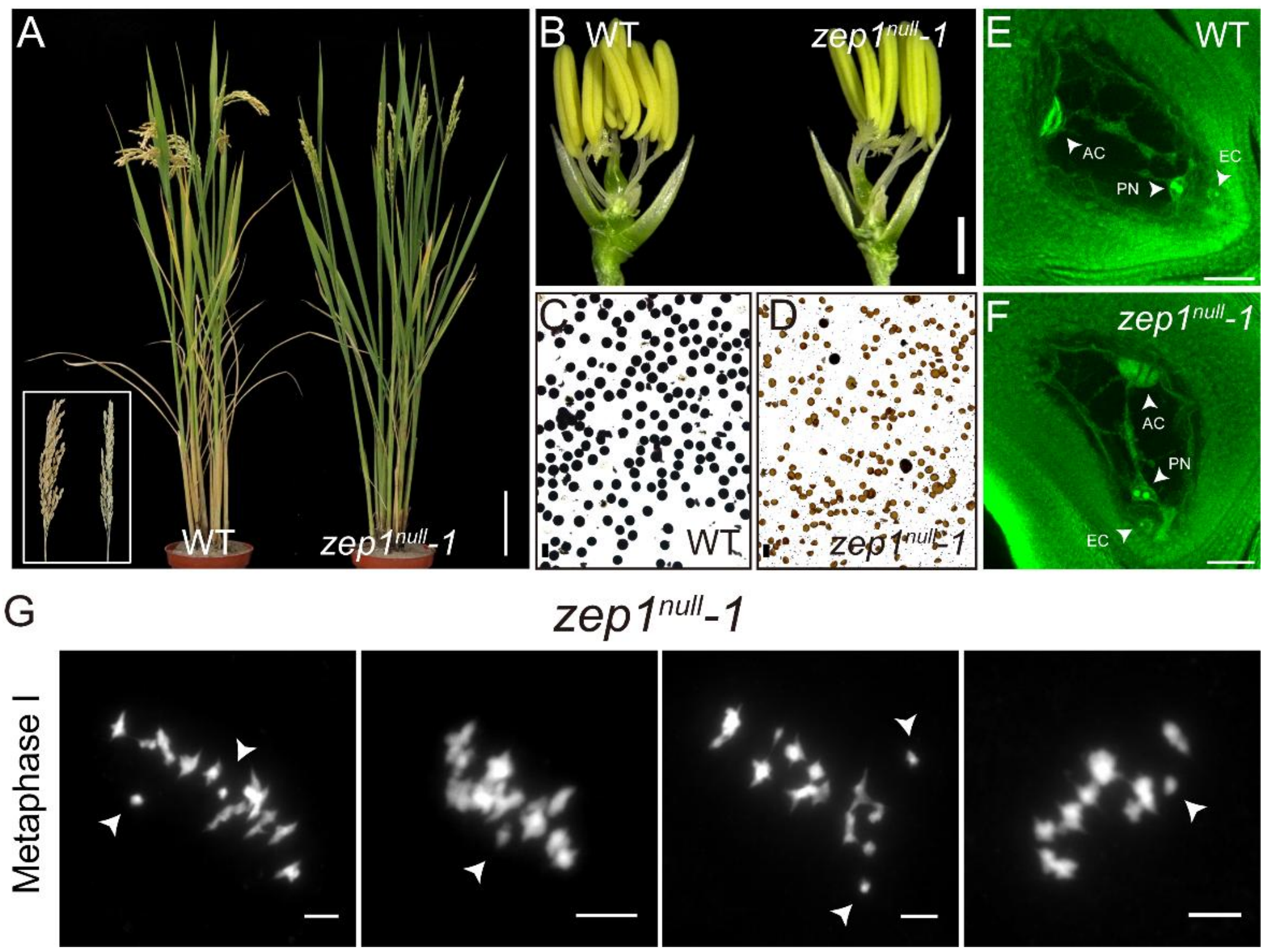

Appendix Figure S3. Morphological and cytological analysis of wild-type and *zep1^{null}-1* plants.

(A) Plants and panicles (inset) at maturity. Scale bar, 10 cm. **(B)** Wild-type and *zep1^{null}-1* spikelets at heading stage after removal of the lemma and palea. Scale bar, 1 mm. **(C)-(D)** I_2-KI staining of wild-type (C) and *zep1^{null}-1* (D) mature pollen grains, where dark staining indicates viable pollen grains. Scale bar, 100 μm. **(E)-(F)** Mature embryo sac in wild type (E) and *zep1^{null}-1* (F), showing the antipodal cell (AC), polar nucleus (PN), and egg cell (EC) (white arrowheads). Scale bar, 40 μm. **(G)** The appearance of univalents (white arrowheads) in *zep1^{null}-1* at meiosis metaphase I. Meiocytes were scored from chromosome spreads generated using pooled anthers dissected from meiotic florets collected from independent plants of the same genotype. Meiotic chromosomes are indicated by DAPI staining. Scale bar, 5 μm.

A

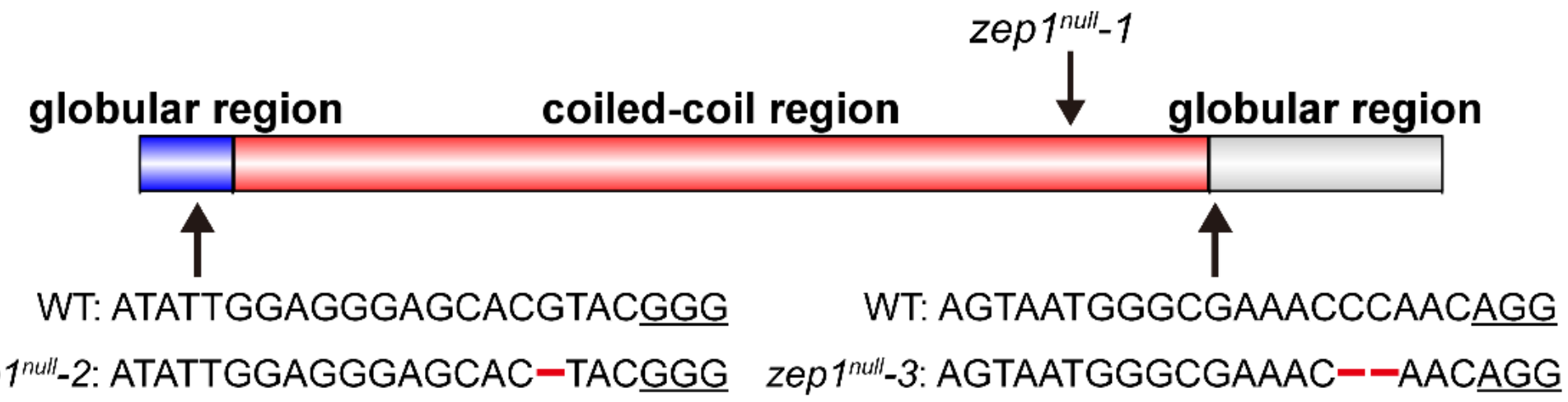

B

N-globular region

```
WT         MQKLGLSGLRGLEGFRSLAGSTSTAAKAPNPKPSSDIGGSTYGSFANLKITAEKLVKEQASVKTDLEMTHTKLRRATEQI 80
zep1null-3 MQKLGLSGLRGLEGFRSLAGSTSTAAKAPNPKPSSDIGGSTYGSFANLKITAEKLVKEQASVKTDLEMTHTKLRRATEQI 80
zep1null-1 MQKLGLSGLRGLEGFRSLAGSTSTAAKAPNPKPSSDIGGSTYGSFANLKITAEKLVKEQASVKTDLEMTHTKLRRATEQI 80
zep1null-2 MQKLGLSGLRGLEGFRSLAGSTSTAAKAPNPKPSSDIGGSTTGASPILRSQQKSWLRSRLQ------------------- 61

WT         NLLEAKLQQAVNENAKLKVKQTEDSKLWQGLDSKVSSTKTLCNQLTETLQQLASQTERAEEDKKFFEEILGKNSKAFEEF 160
zep1null-3 NLLEAKLQQAVNENAKLKVKQTEDSKLWQGLDSKVSSTKTLCNQLTETLQQLASQTERAEEDKKFFEEILGKNSKAFEEF 160
zep1null-1 NLLEAKLQQAVNENAKLKVKQTEDSKLWQGLDSKVSSTKTLCNQLTETLQQLASQTERAEEDKKFFEEILGKNSKAFEEF 160
zep1null-2 -------------------------------------------------------------------------------- 61

WT         NCLLHDSSIKLECAEQMIISGKQEMLRIKQEKEEMDQSYKEQLYASDTTIREKNSLIKQLEDSIEQNKARLLYVDSRLEC 240
zep1null-3 NCLLHDSSIKLECAEQMIISGKQEMLRIKQEKEEMDQSYKEQLYASDTTIREKNSLIKQLEDSIEQNKARLLYVDSRLEC 240
zep1null-1 NCLLHDSSIKLECAEQMIISGKQEMLRIKQEKEEMDQSYKEQLYASDTTIREKNSLIKQLEDSIEQNKARLLYVDSRLEC 240
zep1null-2 -------------------------------------------------------------------------------- 61

WT         MEQELKLKEDVCICLKENLASTESEKNDLKLRNEGYTLEVQKLSKDNKELNELLSGFTVKVTELDKEHTSISSHVTQLIS 320
zep1null-3 MEQELKLKEDVCICLKENLASTESEKNDLKLRNEGYTLEVQKLSKDNKELNELLSGFTVKVTELDKEHTSISSHVTQLIS 320
zep1null-1 MEQELKLKEDVCICLKENLASTESEKNDLKLRNEGYTLEVQKLSKDNKELNELLSGFTVKVTELDKEHTSISSHVTQLIS 320
zep1null-2 -------------------------------------------------------------------------------- 61

WT         SFERYDGKVHEEKMLMIKSAKDKFEHLQNQYVNLISENNALQTEIEELKSRIIELQKTQEIVMVQHVEECQVAEDKIRRL 400
zep1null-3 SFERYDGKVHEEKMLMIKSAKDKFEHLQNQYVNLISENNALQTEIEELKSRIIELQKTQEIVMVQHVEECQVAEDKIRRL 400
zep1null-1 SFERYDGKVHEEKMLMIKSAKDKFEHLQNQYVNLISENNALQTEIEELKSRIIELQKTQEIVMVQHVEECQVAEDKIRRL 400
zep1null-2 -------------------------------------------------------------------------------- 61

WT         ESEAEISASNISQLEKVASDLEGRVQKLLEDSRSAENHKQELLQKILKLESDNQELLGRVQSVLNEKSNDTESLQGEIAK 480
zep1null-3 ESEAEISASNISQLEKVASDLEGRVQKLLEDSRSAENHKQELLQKILKLESDNQELLGRVQSVLNEKSNDTESLQGEIAK 480
zep1null-1 ESEAEISASNISQLEKVASDLEGRVQKLLEDSRSAENHKQELLQKILKLESDNQELLGRVQSVLNEKSNDTESLQGEIAK 480
zep1null-2 -------------------------------------------------------------------------------- 61

WT         RDQQVETLENQVNQLCSIIDEKEQLHTCAVEREKNLEEQKLQVQASLAATESQLTEAKKQYDIMLEGKKIELSKHLKELS 560
zep1null-3 RDQQVETLENQVNQLCSIIDEKEQLHTCAVEREKNLEEQKLQVQASLAATESQLTEAKKQYDIMLEGKKIELSKHLKELS 560
zep1null-1 RDQQVETLENQVNQLCSIIDEKEQLHTCAVEREKNLEEQKLQVQASLAATESQLTEAKKQYDIMLEGKKIELSKHLKELS 560
zep1null-2 -------------------------------------------------------------------------------- 61

WT         LKNDQAINEIRRKYELEKVEIINIEKEKAEKLIKEMENKCNEKISENRQDSERYLMCLKEEHGSMVARIQQDNEHKESTL 640
zep1null-3 LKNDQAINEIRRKYELEKVEIINIEKEKAEKLIKEMENKCNEKISENRQDSERYLMCLKEEHGSMVARIQQDNEHKESTL 640
zep1null-1 LKNDQAINEIRRKYELEKVEIINIEKEKAEKLIKEMENKCNEKISENRQDSERYLMCLKEEHGSMVARISAG-------- 632
zep1null-2 -------------------------------------------------------------------------------- 61
```

C-globular region

```
WT         RAYHKEELQRIQSQAENELRERLSSLRKDHEIQMKSLTKKHEENCQKLQDELELQKSKEEKQRALLQLQWKVMGETQQVD 720
zep1null-3 RAYHKEELQRIQSQAENELRERLSSLRKDHEIQMKSLTKKHEENCQKLQDELELQKSKEEKQRALLQLQWKVMGETTG-- 718
zep1null-1 -------------------------------------------------------------------------------- 632
zep1null-2 -------------------------------------------------------------------------------- 61

WT         QEVNSKKEYSVSSIKRRDPYIRKEHQLQLVSPETKRKDVNLSGIIQSPITNMLRKVEKGTQDIPKHRKVTHHEYEVETAN 800
zep1null-3 -------------------------------------------------------------------------------- 718
zep1null-1 -------------------------------------------------------------------------------- 632
zep1null-2 -------------------------------------------------------------------------------- 61

WT         GRITKRRKTKSTVMFGEPNTQKSLHDTADKDPTKMKKVVAGSHPHPANIGELFSEGSLNPYAEDPYAFG 869
zep1null-3 --------------------------------------------------------------------- 718
zep1null-1 --------------------------------------------------------------------- 632
zep1null-2 --------------------------------------------------------------------- 61
```

Appendix Figure S4. Generation of *zep1^{null}* CRISPR lines (*zep1^{null}-2*, *zep1^{null}-3*).
(A) Three *zep1^{null}* mutants mutate in three different domains of ZEP1 protein. Arrows indicate mutation site in *zep1^{null}-1* and the other two CRISPR lines. Both *zep1^{null}-2* and *zep1^{null}-3* have short deletions (indicated by red lines). **(B)** Amino acid sequence alignment of ZEP1 protein of WT and mutants. The *ZEP1* gene encodes a protein containing 869 amino acids, while all three *zep1^{null}* mutants caused a frameshift and premature translation termination of ZEP1 protein in N-terminal globular domain (*zep1^{null}-2*, red box), C-terminal globular domain (*zep1^{null}-3*, red box) and central coiled-coil region (*zep1^{null}-1*).

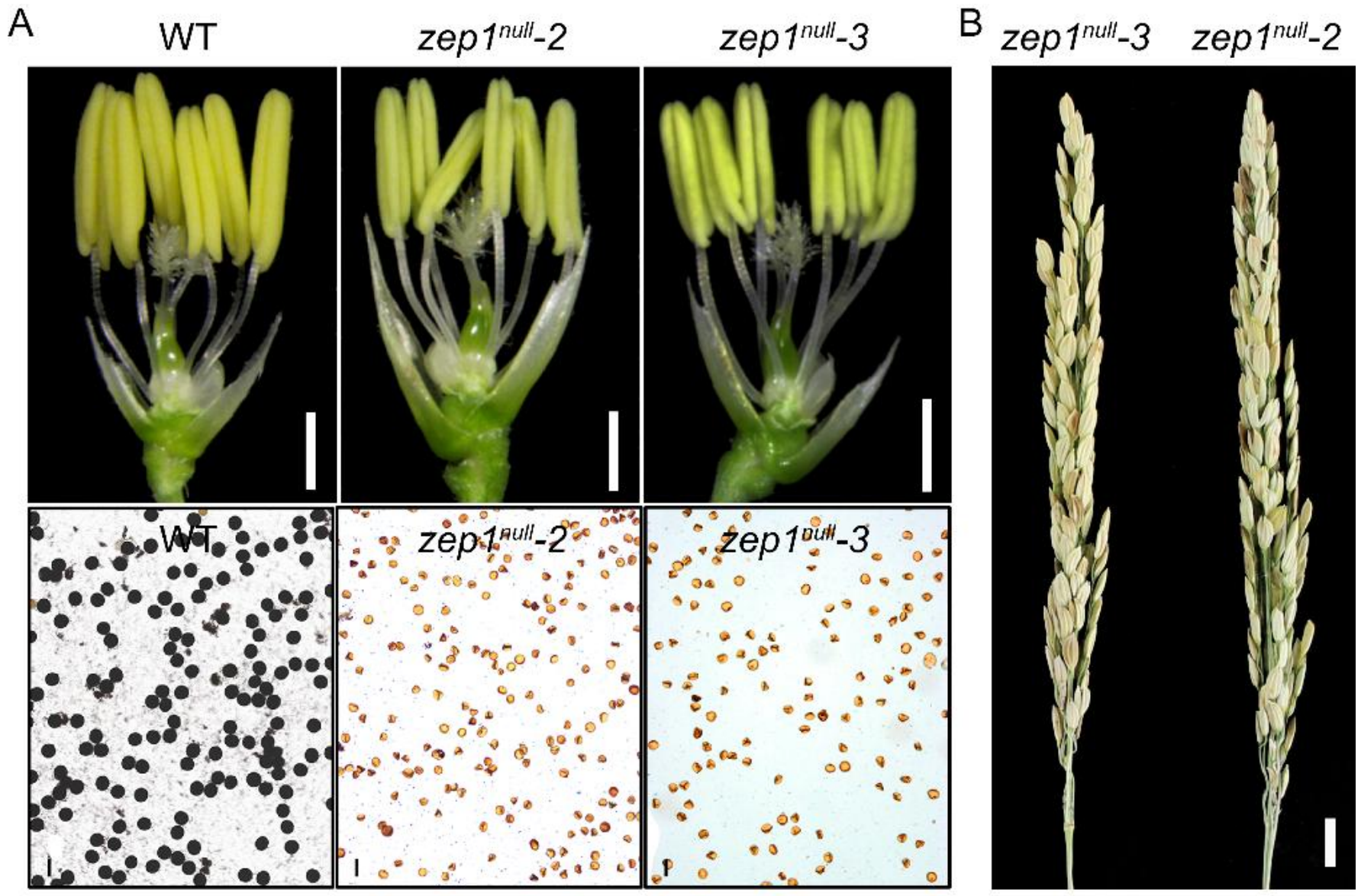

Appendix Figure S5. *zep1null* CRISPR lines exhibit defects in male fertility.
(A) *zep1null* CRISPR lines are both male sterile. Flower morphology and pollen I_2–KI staining of wild type and *zep1null* CRISPR lines. Scale bars, 1 mm (flower) and 100 µm (pollen). **(B)** Panicles of *zep1null-2* and *zep1null-3* mutants at maturity. Scale bar, 1 cm.

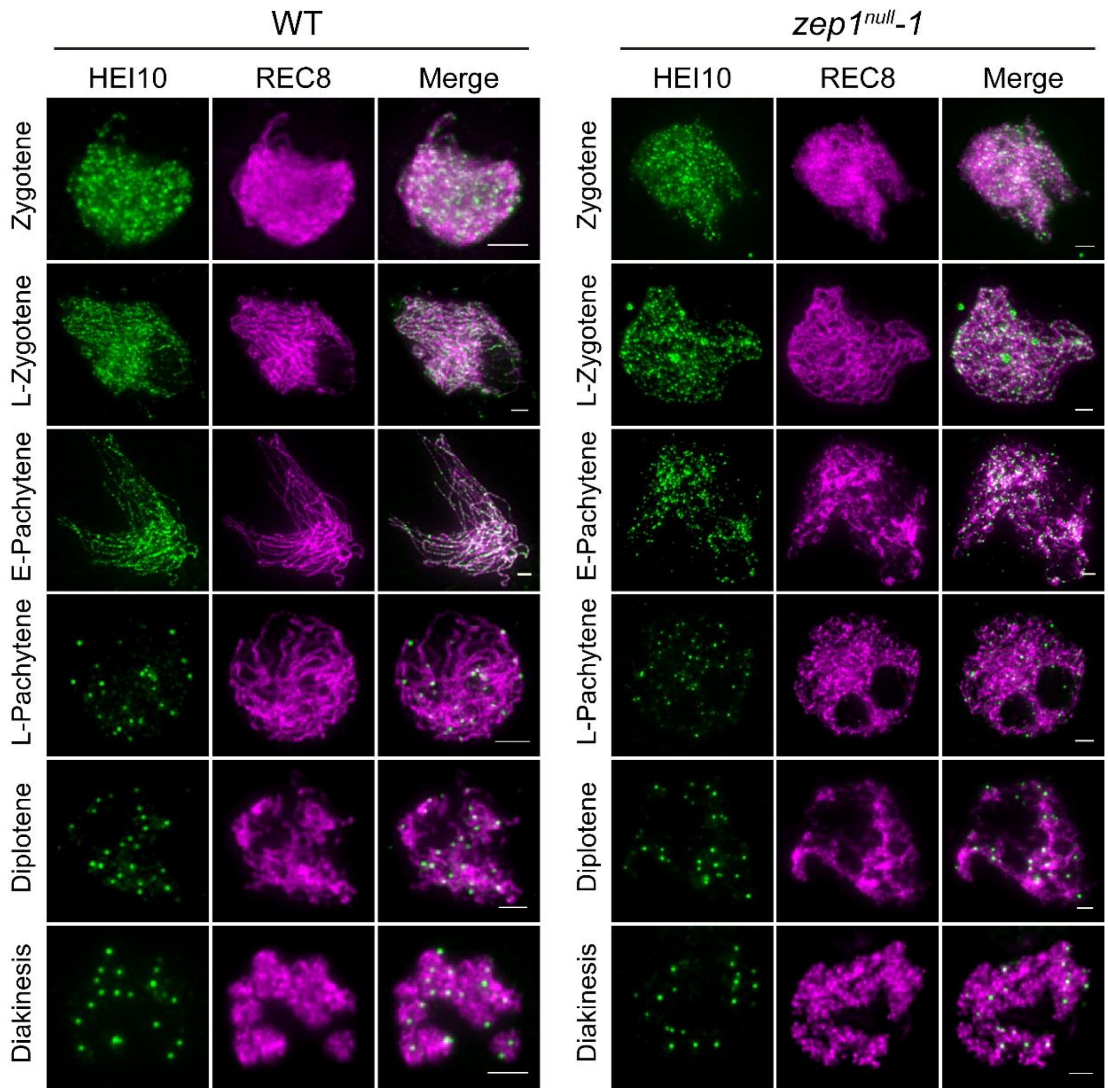

Appendix Figure S6. The mature HEI10 protein foci number does not change in *zep1^{null}-1* male meiocytes at later prophase I.

Localization dynamics of HEI10 protein foci (green) during meiosis prophase I in wild-type and *zep1^{null}-1*. Meiotic chromosomes are visualized by axis protein REC8 (magenta). Meiocytes were scored from chromosome spreads generated using pooled anthers dissected from meiotic florets collected from independent plants of the same genotype. Multiple independent slide preparations were examined to confirm the reproducibility of the observed phenotypes. Scale bar, 2 μm.

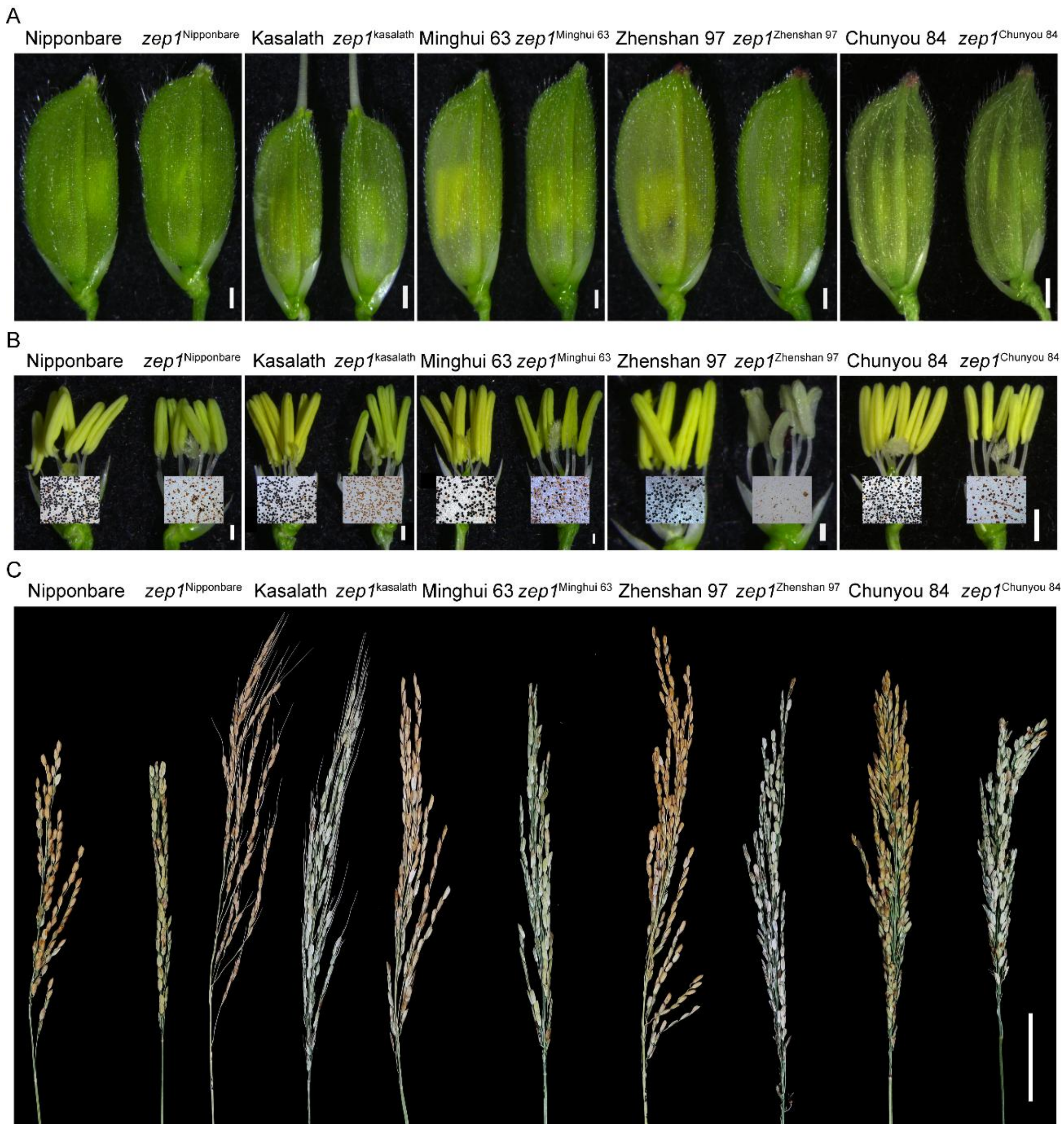

Appendix Figure S7. *zep1* null lines of different rice varieties are all male sterile.
(A) Spikelet morphology of wild type and *zep1* null lines. Scale bars, 1 mm. **(B)** The morphology of flower and pollen I_2–KI staining of wild type and *zep1* null lines. Scale bars, 1 mm (flower) and 50 μm (pollen). **(C)** Panicles of z*ep1* null lines at maturity. Scale bar, 10 cm.

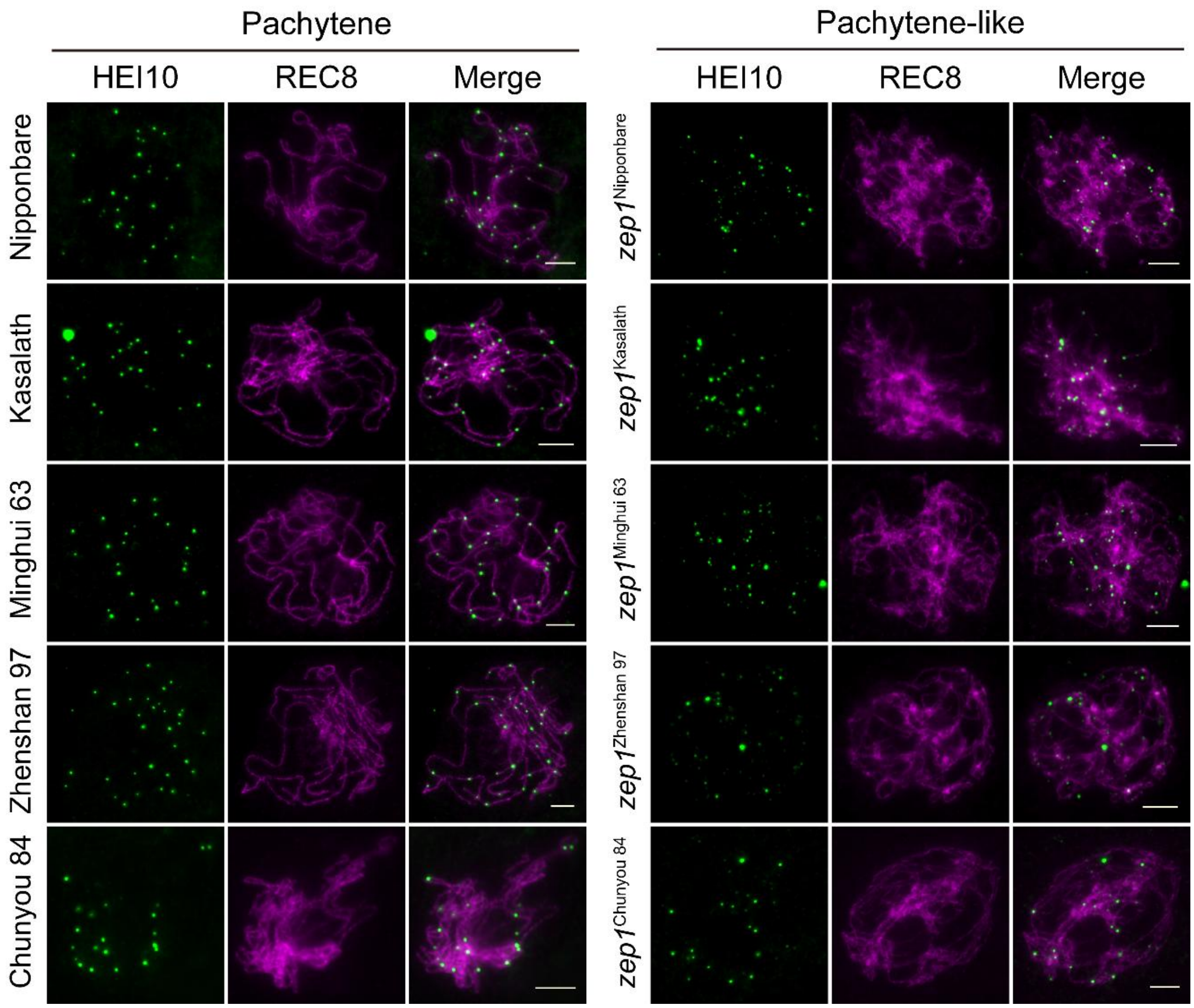

Appendix Figure S8. The HEI10 protein foci dynamics in wild-type and *zep1* null male meiocytes of different rice varieties.

Immunodetection of HEI10 protein foci (green) in wild type and another *zep1* null mutants (*O. sativa* ssp. *Japonica* cv. Nipponbare, *O. sativa ssp. Indica* cv. Kasalath/Minghui 63/Zhenshan 97 and inter-subspecific hybrid rice chunyou 84) at pachytene (or pachytene-like) stage. Meiotic chromosomes are visualized by axis protein REC8 (magenta). Meiocytes were scored from chromosome spreads of pooled anthers isolated from meiotic florets of independent isogenic plants. Multiple independent slide preparations were examined to confirm the reproducibility of the observed phenotypes. Scale bars, 5 µm.

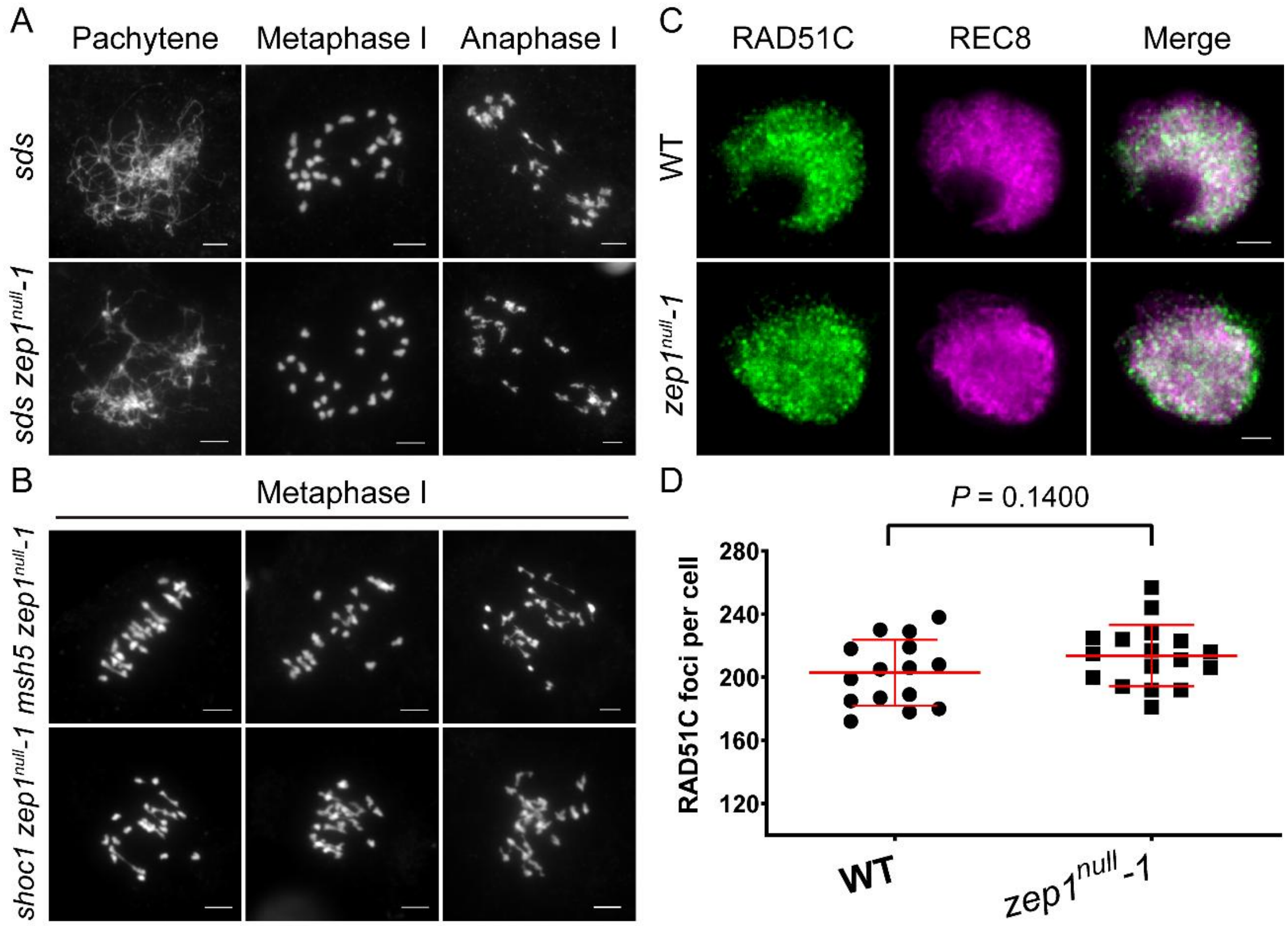

Appendix Figure S9. Chromosome behavior in meiosis-related mutants and RAD51C protein localization in *zep1* male meiosis.

(A) Chromosome behaviors during meiosis prophase I in the *sds zep1^{null}-1* double mutant is similar to that in the *sds* single mutant. **(B)** Diverse chromosome behaviors at metaphase I in *msh5 zep1^{null}-1* and *shoc1 zep1^{null}-1* male meiocytes. Meiocytes were scored from chromosome spreads generated via multiple independent slide preparations using pooled anthers isolated from meiotic florets collected from independent plants of the same genotype. Meiotic chromosomes are visualized by DAPI staining. Scale bar, 5 μm. **(C)** The chromosomal loading of RAD51C (green) protein in *zep1^{null}-1* male meiocytes is similar to that in wild type at zygotene. Meiotic chromosomes are visualized by axis protein REC8 (magenta). Scale bars, 2 μm. **(D)** Comparison of RAD51C protein foci numbers revealed no significant differences between wild-type and *zep1^{null}-1* zygotene meiocytes. A total of n = 15 WT and n = 17 *zep1^{null}-1* zygotene meiocytes were scored from chromosome spreads generated using pooled anthers dissected from meiotic florets collected from independent plants of the same genotype. Multiple independent slide preparations were examined to confirm the reproducibility of the observed phenotypes, with all quantified cells incorporated into the plotted dataset. Each dot corresponds to a single meiocyte. Bars denote mean ± SD of the quantified cell population. Statistical significance was determined via two-tailed Student's *t* test. Statistical *P*-values derived from the corresponding quantitative analyses are indicated within the figure panels.

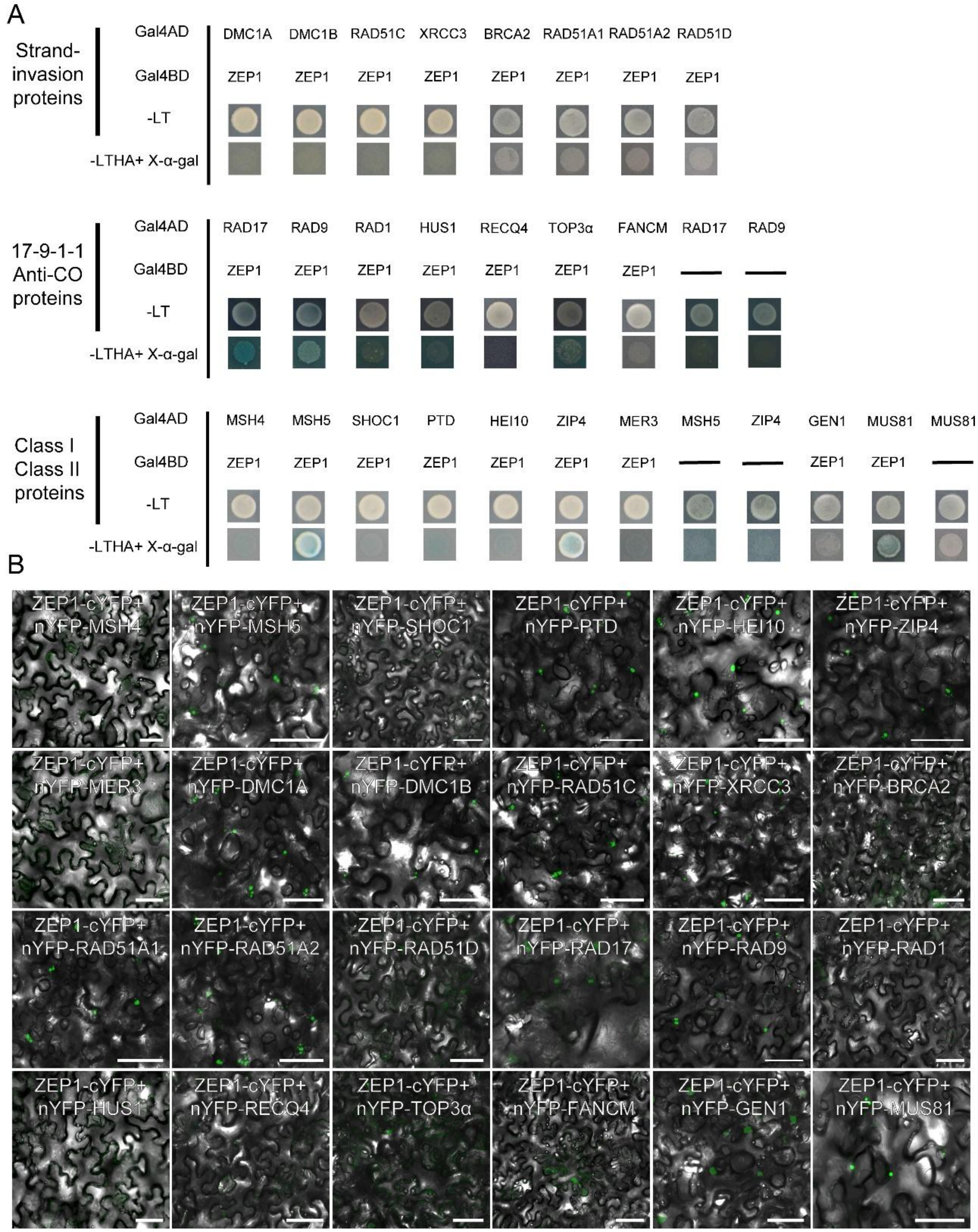

Appendix Figure S10. Interaction of ZEP1 protein with rice proteins known to be involved in meiosis process.

(A) Protein-protein interactions were tested using the yeast two-hybrid (Y2H) system. Transformants were plated on selective media SD–Leu/–Trp (−LT) to select for transformants and on SD–Leu/–Trp/–His/–Ade (−LTHA) supplemented with X-α-gal to assess positive interactions. The appearance of well-grown blue colonies on quadruple dropout selective plates confirm protein–protein interactions. **(B)** Bimolecular fluorescence complementation (BiFC) was employed to detect protein–protein interactions. ZEP1 was fused to the C-terminal YFP fragment (cYFP), while each candidate interacting protein was fused to the N-terminal YFP fragment (nYFP). Pairs exhibiting positive interaction all produce punctate nuclear signals in tobacco cells. Scale bars = 50 μm.

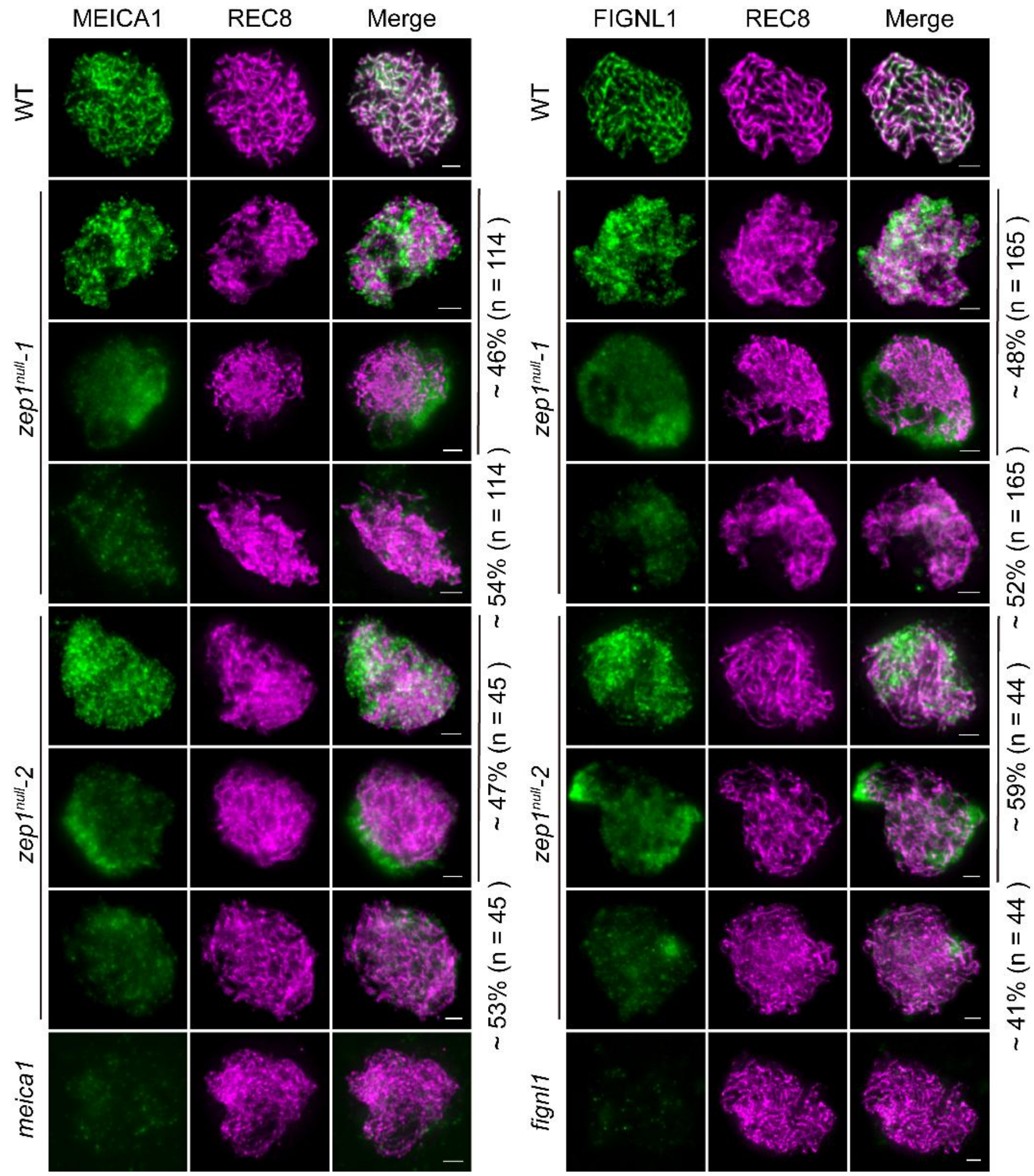

Appendix Figure S11. Immunodetection of MEICA1 and FIGNL1 proteins in wild-type and mutant male meiocytes.

Representative images showing failed chromosomal recruitment and extrachromosomal mislocalization of MEICA1 and FIGNL1 protein foci (green) in *zep1* null meiocytes. Meiotic chromosomes are visualized by axis protein REC8 (magenta). Representative images of MEICA1 and FIGNL1 localization in wild-type and *zep1*^{null}*-1* meiocytes were obtained from the same experiment as the images shown in Figure 5C. Additional images are provided here for a more comprehensive comparison. “n” in the panels represents the number of meiocytes displaying the indicated phenotype over the total number of analyzed meiocytes in each group. A total of n = 114 *zep1*^{null}*-1* and n = 45 *zep1*^{null}*-2* prophase I meiocytes were scored for MEICA1 foci. For FIGNL1 foci, n = 165 *zep1*^{null}*-1* and n = 44 *zep1*^{null}*-2* prophase I meiocytes were scored. Meiocytes were scored from chromosome spreads prepared with pooled anthers isolated from meiotic florets of independent plants of the same genotype. Multiple independent slide preparations were examined to confirm the reproducibility of the observed phenotypes. Scale bar, 2 μm.

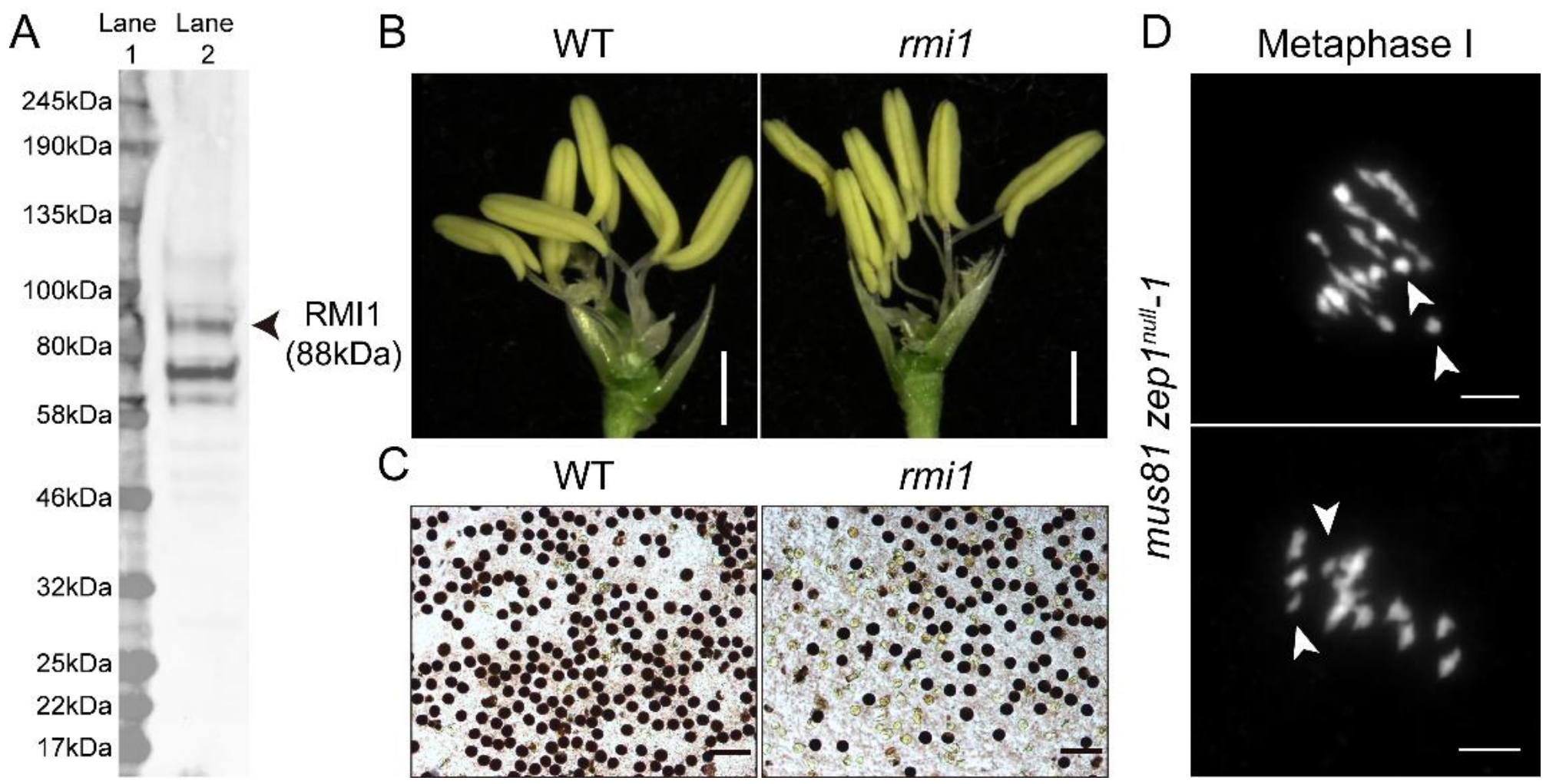

Appendix Figure S12. The phenotype of *rmi1* hypomorphic allele and meiotic chromosome behavior of *mus81 zep1*^{null}*-1* mutants.
(A) Immunoblot detection of RMI1 protein using an anti-RMI1 antibody in wild-type meiotic spikelets. Lane 1: Protein standard; Lane 2: Proteins isolated from wild-type meiotic spikelets. **(B)-(C)** A partial loss-of-function mutation in rice *RMI1* causes partial male sterility. Rice spikelets (at heading stage) had thinner, paler anthers (B) that contained partial viable mature pollen grains in *rmi1* (C). Scale bars, 1 mm (flower) and 100 μm (pollen). **(D)** Diverse chromosome behaviors at metaphase I in *mus81 zep1*^{null}*-1* male meiocytes. Meiotic chromosomes are visualized by DAPI staining. Meiocytes were scored from chromosome spreads generated via multiple independent slide preparations using pooled anthers isolated from meiotic florets collected from independent plants of the same genotype. Univalents are indicated by white arrowheads. Scale bar, 5 μm.

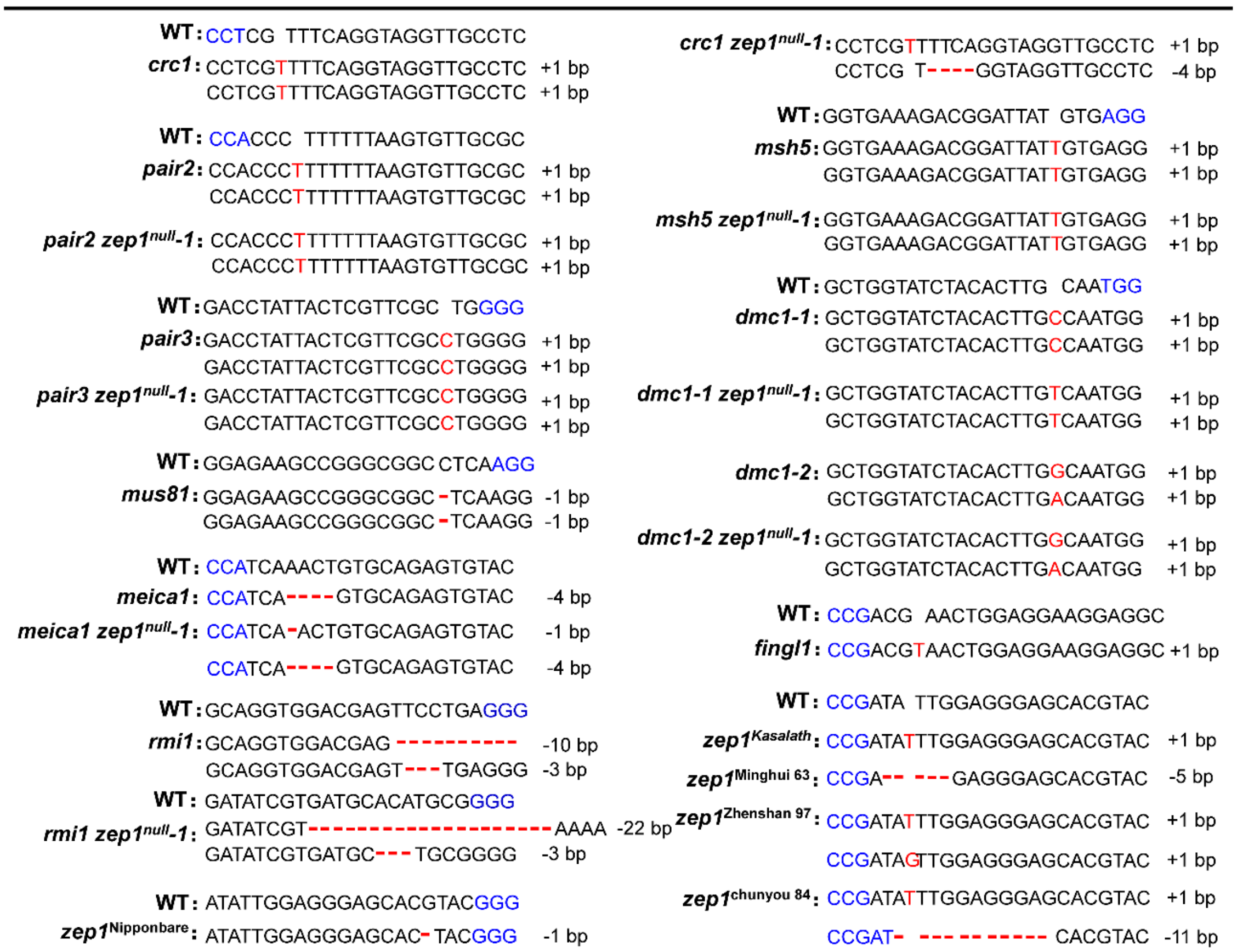

Appendix Figure S13. WT target sequences used for genome-editing in meiosis-related genes.
PAM sequences are shown in blue. Red indicated the generated mutation (nucleotide insertion or deletion [indicated by dash]).

Appendix Table S1. Primers used in this study.

Primer name	Forward sequence (5′→3′)	Reverse sequence (5′→3′)	Objective
SNP4	CTTAGGTTAGGTAAAAGGGTT	AGTCACATTCTGATACAACGA	Map-based cloning
HY002	GTGAATCCATCGTGAGTGTG	TATTCCCTCGATAAGCGAAA	Map-based cloning
pGBKT7-ZEP1	CATGGAGGCCGAATTCATGCAGAAGCTGGGTTTA	GCAGGTCGACGGATCCCTAGCCAAATGCATAAGG	Y2H
pGADT7-ZEP1	GGAGGCCAGTGAATTCATGCAGAAGCTGGGTTTA	CGAGCTCGATGGATCCCTAGCCAAATGCATAAGG	Y2H
pGADT7-DMC1A	AGGAGGACCTGCATATGGCGCCGTCCAAGCAGTACAG	TGCAGGTCGACGGATCCTCAGTCTTTCGCATCCATTAT	Y2H
pGADT7-DMC1B	AGGAGGACCTGCATATGGCGCCGTCCAAGCAGTACGA	TGCAGGTCGACGGATCCTCAGTCTTTCGCATCCATTAT	Y2H
pGADT7-RAD51A1	CAGATTACGCTCATATGTCGACGTCGGCGGCGGCGTCG	CGAGCTCGATGGATCCTCAATCCTTGACATCTGCAAC	Y2H
pGADT7-RAD51A2	CAGATTACGCTCATATGTCGTCGTCGGGTGCGGCTGCG	CGAGCTCGATGGATCCTCAGTCCTTAACATCTGTGAC	Y2H
pGADT7-RAD51C	CAGATTACGCTCATATGGAGATCGCCGACCTCCCCATC	CGAGCTCGATGGATCCCTACATTACCCGGACTCGCTT	Y2H
pGADT7-RAD51D	CAGATTACGCTCATATGGCGATGGCGGCGACGTCGGCG	CGAGCTCGATGGATCCTCAGCTTGGTACGGCAAATTTC	Y2H
pGADT7-XRCC3	CAGATTACGCTCATATGCGGCCGGCGGCGCCGGCAAGG	CGAGCTCGATGGATCCTTACCTTCCCGACGGCTCGAC	Y2H
pGADT7-BRCA2	GGAGGCCAGTGAATTCATGCAGAGGAGGTGGCAGGTGT	CGAGCTCGATGGATCCTCAGCCACCACTGTCACCAACTAT	Y2H
pGADT7-RAD17	GGAGGCCAGTGAATTCATGGGGAAGCGGCCGCC	CGAGCTCGATGGATCCTCACCAATCTTCTATCTCA	Y2H
pGADT7-RAD9	GGAGGCCAGTGAATTCATGGCTTCGGGAGAGAAAAACC	GAGCTCGATGGATCCCTAGTCCATGTAGTGCGGTG	Y2H
pGADT7-RAD1	GGAGGCCAGTGAATTCATGAGCTCGTCGACGTCCG	CGAGCTCGATGGATCCCTACGCATCATTTATCTC	Y2H
pGADT7-HUS1	GGAGGCCAGTGAATTCATGAAGTTCAAGGCCTTC	CGAGCTCGATGGATCCTTAACTGCCAGGGTCAAG	Y2H
pGADT7-GEN1	GGAGGCCAGTGAATTCATGGGGGTGGGGGGAAGCTT	CGAGCTCGATGGATCCTCAGTCGAAGAGGAGGCGT	Y2H
pGADT7-MUS81	GGAGGCCAGTGAATTCATGGCGCCGGAGGCGAGGCAA	CGAGCTCGATGGATCCTTATCCTTCAGCCCAGACAA	Y2H
pGADT7-PAIR2	GGAGGCCAGTGAATTCATGGTGATGGCTCAGAA	CGAGCTCGATGGATCCTCACTGAACTTGAACTTGA	Y2H
pGADT7-PAIR3	GGAGGCCAGTGAATTCATGGAAGTTGAGCTGACG	CGAGCTCGATGGATCCTTACTCTGCAGTTTCTGC	Y2H
pGADT7-CRC1	GGAGGCCAGTGAATTCATGAGCGCCCCCATGGAG	CGAGCTCGATGGATCCTCAACCACGCGACTCGGA	Y2H
pGADT7-MEICA1	GGAGGCCAGTGAATTCATGGAGGCTGATGGTGGCGCT	CGAGCTCGATGGATCCTTAAATTTTGTCAGAAAAAGAGG	Y2H
pGADT7-FIGNL1	GGAGGCCAGTGAATTCATGGCGGAGCAGTCTCAC	CGAGCTCGATGGATCCTTAATTTGCTAAGCTCCC	Y2H
pGADT7-RMI1	GGAGGCCAGTGAATTCATGAGGCGCCGAAACCTAAT	CGAGCTCGATGGATCCTCATGGAGTGGTGTCCATAA	Y2H
pGADT7-TOP3α	GGAGGCCAGTGAATTCATGCACCACGGCGGCGG	CGAGCTCGATGGATCCCTATCTCCCGAGGTTGG	Y2H
pGADT7-RECQ4	GGAGGCCAGTGAATTCATGATAAAGCCAAGGGTCAA	CGAGCTCGATGGATCCCTAGGCTATTCTGGCGGACT	Y2H
pGADT7-FANCM	GGAGGCCAGTGAATTCATGGCCGCGCCGCCTCCGCCG	CGAGCTCGATGGATCCCTAGTTCCCAAAGGATCTCTG	Y2H
pGADT7-MHS4	GGAGGCCAGTGAATTCATGGAGGGCGACGCCGCC	CGAGCTCGATGGATCCTCATGTCAAGCGGCCTGC	Y2H
pGADT7-MSH5	GGAGGCCAGTGAATTCATGGACGAGGAAGAGGAG	CGAGCTCGATGGATCCCTAGGACGGAAAAACTTC	Y2H
pGADT7-SHOC1	GGAGGCCAGTGAATTCATGAGGACCCGCTTCCTC	CGAGCTCGATGGATCCCTACGTACCTTCCTCTCG	Y2H
pGADT7-PTD	GGAGGCCAGTGAATTCATGGAGCGCTCCACCCAC	CGAGCTCGATGGATCCCTAGTTAATTTTAGGGCT	Y2H
pGADT7-HEI10	GGAGGCCAGTGAATTCATGAAGTGCAATGCTTGC	CGAGCTCGATGGATCCTTACGTGAACATATGTGG	Y2H
pGADT7-ZIP4	GGAGGCCAGTGAATTCATGAAGATCTCCGAGCTC	CGAGCTCGATGGATCCCTAAACTAGAACAGGTTG	Y2H
pGADT7-MER3	GGAGGCCAGTGAATTCATGGCGGCGATGGGACAT	CGAGCTCGATGGATCCTCAGAGAAATGAGAAGAC	Y2H
MEICA1-AnB	GGTTCCGCGTGGATCCGATGAGGATGGGTTTATGT	GATGCGGCCGCTCGAGGATTTTATCAGAGAAACTAGC	Antibody of MEICA1
FIGNL1-AnB	GGTTCCGCGTGGATCCAACTGGAGGAAGGAGGCGGA	GATGCGGCCGCTCGAGACGCAGTGATGTTAGTTTTCTT	Antibody of FIGNL1
RMI1-AnB	GGTTCCGCGTGGATCCCTGCGGCAGGTGGACGAGTT	GATGCGGCCGCTCGAGTGCTAGAGTGGCTCTCGAAG	Antibody of RMI1
ZEP1-CRISPR-2	TAGGTCTCCTCCCTCCAATATGTTTTAGAGCTAGAA	CGGGTCTCAGGGAGCACGTACTGCACCAGCCGGG	Knock-out of ZEP1
ZEP1-CRISPR-3	TAGGTCTCCGCGAAACCCAACGTTTTAGAGCTAGAA	CGGGTCTCATCGCCCATTACTTGCACCAGCCGGG	Knock-out of ZEP1
CRC1-CRISPR	TAGGTCTCCCTACCTGAAACGGTTTTAGAGCTAGAA	CGGGTCTCAGTAGGTTGCCTCTGCACCAGCCGGG	Knock-out of CRC1
DMC1A/B-CRISPR	TAGGTCTCCCTACACTTGCAAGTTTTAGAGCTAGAA	CGGGTCTCAGTAGATACCAGCTGCACCAGCCGGG	Knock-out of DMC1
PAIR2-CRISPR	TAGGTCTCCCTTAAAAAAGGGGTTTTAGAGCTAGAA	CGGGTCTCATAAGTGTTGCGCTGCACCAGCCGGG	Knock-out of PAIR2
PAIR3-CRISPR	TAGGTCTCCACTCGTTCGCTGGTTTTAGAGCTAGAA	CGGGTCTCAGAGTAATAGGTCTGCACCAGCCGGG	Knock-out of PAIR3
MSH5-CRISPR	TAGGTCTCCACGGATTATGTGGTTTTAGAGCTAGAA	CGGGTCTCACCGTCTTTCACCTGCACCAGCCGGG	Knock-out of MSH5
MUS81-CRISPR	TGGCGGAGAAGCCGGGCGGCCTCA	AAACTGAGGCCGCCCGGCTTCTCC	Knock-out of MUS81
MEICA1-CRISPR	TAGGTCTCCTGCACAGTTTGAGTTTTAGAGCTAGAA	CGGGTCTCATGCAGAGTGTACTGCACCAGCCGGG	Knock-out of MEICA1
FIGNL1-CRISPR	TAGGTCTCCCCTCCAGTTCGTGTTTTAGAGCTAGAA	CGGGTCTCAGAGGAAGGAGGCTGCACCAGCCGGG	Knock-out of FIGNL1
RMI1-CRISPR-1	TAGGTCTCCACGAGTTCCTGAGTTTTAGAGCTAGAA	CGGGTCTCATCGTCCACCTGCTGCACCAGCCGGG	Knock-out of RMI1
RMI1-CRISPR-2	TAGGTCTCCGATGCACATGCGGTTTTAGAGCTAGAA	CGGGTCTCACATCACGATATCTGCACCAGCCGGG	Knock-out of RMI1
ZEP1-cYFP	AGGTACCCGGGGATCCATGCAGAAGCTGGGTTTA	CGACTCTAGAGGATCCGCCAAATGCATAAGGATC	BIFC assay
MEICA1-nYFP	GGACGCCGGCGGATCCATGGAGGCTGATGGTGGC	CGACTCTAGAGGATCCTTAAATTTTGTCAGAAAAAGAGGCC	BIFC assay
FIGNL1-nYFP	GGACGCCGGCGGATCCATGGCGGAGCAGTCTCAC	CGACTCTAGAGGATCCTTAATTTGCTAAGCTCCCAA	BIFC assay
RMIL1-nYFP	GGACGCCGGCGGATCCATGAGGCGCCGAAACCTAATC	CGACTCTAGAGGATCCTCATGGAGTGGTGTCCATAA	BIFC assay
RAD17-nYFP	GGACGCCGGCGGATCCATGGGGAAGCGGCCGCCGGT	CGACTCTAGAGGATCCTCACCAATCTTCTATCTCAT	BIFC assay
RAD9-nYFP	GGACGCCGGCGGATCCATGGCTTCGGGAGAGAAAAACC	CGACTCTAGAGGATCCCTAGTCCATGTAGTGCGGTG	BIFC assay
RAD1-nYFP	GCCGGCGGATCCTCTAGAGTCGACATGAGCTCGTCGACGTCCG	CGAACGAAAGCTCTGCAGGTCGACCGCATCATTTATCTCATATTCCTC	BIFC assay
HUS1-nYFP	GCCGGCGGATCCTCTAGAGTCGACATGAAGTTCAAGGCCTTC	CGAACGAAAGCTCTGCAGGTCGACTTAACTGCCAGGGTCAAG	BIFC assay
RECQ4-nYFP	GCCGGCGGATCCTCTAGAGTCGACATGATAAAGCCAAGGGTCAAC	CGAACGAAAGCTCTGCAGGTCGACCTAGGCTATTCTGGCGGACTG	BIFC assay
TOP3α-nYFP	GCCGGCGGATCCTCTAGAGTCGACATGCACCACGGCGGCGG	CGAACGAAAGCTCTGCAGGTCGACCTATCTCCCGAGGTTGG	BIFC assay
FANCM-nYFP	GCCGGCGGATCCTCTAGAGTCGACATGGCCGCGCCGCCTCCG	CGAACGAAAGCTCTGCAGGTCGACGTTCCCAAAGGATCTCTGTGTG	BIFC assay
DMC1A-nYFP	GCCGGCGGATCCTCTAGAGTCGACATGGCGCCGTCCAAGCA	CGAACGAAAGCTCTGCAGGTCGACTCAGTCTTTCGCATCCATTATTCCAC	BIFC assay
DMC1B-nYFP	GCCGGCGGATCCTCTAGAGTCGACATGGCGCCGTCCAAGCAGT	CGAACGAAAGCTCTGCAGGTCGACTCAGTCTTTCGCATCCATTA	BIFC assay
RAD51C-nYFP	GCCGGCGGATCCTCTAGAGTCGACATGGAGATCGCCGACCTCCCCAT	CGAACGAAAGCTCTGCAGGTCGACCTACATTACCCGGACTCGCT	BIFC assay
XRCC3-nYFP	GCCGGCGGATCCTCTAGAGTCGACATGCGGCCGGCGGCGCCGGC	CGAACGAAAGCTCTGCAGGTCGACTTACCTTCCCGACGGCTCGA	BIFC assay
BRCA2-nYFP	GCCGGCGGATCCTCTAGAGTCGACATGCAGAGGAGGTGGCAG	CGAACGAAAGCTCTGCAGGTCGACTCAGCCACCACTGTCACC	BIFC assay
RAD51A1-nYFP	GCCGGCGGATCCTCTAGAGTCGACATGTCGACGTCGGCGGCGG	CGAACGAAAGCTCTGCAGGTCGACTCAATCCTTGACATCTGCAA	BIFC assay
RAD51A2-nYFP	GCCGGCGGATCCTCTAGAGTCGACATGTCGTCGTCGGGTGCGGC	CGAACGAAAGCTCTGCAGGTCGACTCAGTCCTTAACATCTGTGA	BIFC assay
RAD51D-nYFP	GCCGGCGGATCCTCTAGAGTCGACATGGCGATGGCGGCGAC	CGAACGAAAGCTCTGCAGGTCGACTCAGCTTGGTACGGCAAATTTCA	BIFC assay
MSH4-nYFP	GCCGGCGGATCCTCTAGAGTCGACATGGAGGGCGACGCCGCCGGA	CGAACGAAAGCTCTGCAGGTCGACTCATGTCAAGCGGCCTGCA	BIFC assay
MSH5-nYFP	GGACGCCGGCGGATCCATGGACGAGGAAGAGGAGGA	CGACTCTAGAGGATCCCTAGGACGGAAAAACTTCCT	BIFC assay
SHOC1-nYFP	GGACGCCGGCGGATCCATGAGGACCCGCTTCCTCGC	CGACTCTAGAGGATCCCTACGTACCTTCCTCTCGGA	BIFC assay
PTD-nYFP	GGACGCCGGCGGATCCATGGAGCGCTCCACCCACTC	CGACTCTAGAGGATCCCTAGTTAATTTTAGGGCTTA	BIFC assay
HEI10-nYFP	GCCGGCGGATCCTCTAGAGTCGACATGAAGTGCAATGCTTGC	CGAACGAAAGCTCTGCAGGTCGACTTACGTGAACATATGTGG	BIFC assay
ZIP4-nYFP	GGACGCCGGCGGATCCATGAAGATCTCCGAGCTCTC	CGACTCTAGAGGATCCCTAAACTAGAACAGGTTGAG	BIFC assay
MER3-nYFP	GCCGGCGGATCCTCTAGAGTCGACATGGCGGCGATGGGACAT	CGAACGAAAGCTCTGCAGGTCGACTCAGAGAAATGAGAAGAC	BIFC assay
GEN1-nYFP	GCCGGCGGATCCTCTAGAGTCGACATGGGGGTGGGGGGAAGCTT	CGAACGAAAGCTCTGCAGGTCGACTCAGTCGAAGAGGAGGCGT	BIFC assay
MUS81-nYFP	GCCGGCGGATCCTCTAGAGTCGACATGGCGCCGGAGGCGAGGCAA	CGAACGAAAGCTCTGCAGGTCGACTTATCCTTCAGCCCAGACAA	BIFC assay
ZEP1-CRISPR-2-JY	GCAAATCGGTTAGTGCTCTC	ACAAAGGCAGTGCCAGATAT	*zep1*^{null}*-2* identification
ZEP1-CRISPR-3-JY	GATTGTCATCGCTCAGAAAG	GTAGAAAATGTCAAACCTGGA	*zep1*^{null}*-3* identification
CRC1-CRISPR-JY	GAGGGACCTGTTGTATCTGG	ATGCTCAAACTCAATCGCTC	*crc1* identification
DMC1-CRISPR-JY-1	ACATCGCTTGCTATGAGAGG	TCCTGTTATGAAGCCCTGACT	*dmc1a* identification
DMC1-CRISPR-JY-2	GCTTGAGTCCCTCTTGTACTTTTA	AAAACAATGAAAAGAGAACCACA	*dmc1b* identification
PAIR2-JY	TGGACCAAATGCCAGAGGAG	GAAAGGTGGCATCACAGCAC	*pair2* identification
PAIR3-JY	AGGCTGTTGCCTCACCAAAT	CGTGTTCACCCTTCTCTCCC	*pair3* identification
MSH5-JY	GGAAATCTGGGAAGACATAACTG	TACATTGTGGAACGGAGGAAGT	*msh5* identification
ZEP1-JY	CCGTAGGACTTACACATCTATTCT	CAATGCTCTTTGCTTCTCCT	*zep1*^{null}*-1* identification
MUS81-JY	CAAACTCCTCCGAAACCT	GCATTGAGTCAAATTTTTACC	*mus81* identification
MEICA1-JY	ACAATGGTCGTATCAGGTGGT	TAGCTTCAGCGGTACCAAGG	*meica1* identification
FIGNL1-JY	CTTCCTTCCTCAGACGAGCG	GGAGACCCTCGTAAACACGG	*fignl1* identification
RMI1-JY	GTGGAGGAGATCGAGGACCC	CTGAGATGTGTTGACAAGTTATGAG	*rmi1* identification
pREUR-ZEP1-HA	AATCAACTAAGAAAACTAGTATGCAGAAGCTGGGTTTAT	TATGGGTAACTAGAACTAGTGCCAAATGCATAAGGATCTT	Co-IP assay
pREUR-MEICA1-YFP	AATCAACTAAGAAAACTAGTATGGAGGCTGATGGTGGCGCT	CCCTTGCTCACCATACTAGTAATTTTGTCAGAAAAAGAGGCC	Co-IP assay
pREUR-FIGNL1-YFP	AATCAACTAAGAAAACTAGTATGGCGGAGCAGTCTCACGC	CCCTTGCTCACCATACTAGTATTTGCTAAGCTCCCAAATTGC	Co-IP assay
pREUR-RMI1-YFP	AATCAACTAAGAAAACTAGTATGAGGCGCCGAAACCTAATC	CCCTTGCTCACCATACTAGTTGGAGTGGTGTCCATAAAATCC	Co-IP assay
5s rDNA-1	GATCCCATCAGAACTCCGAAGTTAAGCGTGCTTGGGCGAGAG		FISH probe
5s rDNA-2	TAGTACTAGGATGGGTGACCTCCTGGGAAGTCCTCGTGTTGCATCCCTC		FISH probe
CEN-1	CTCACTYCGTGATTCGCGCGGCGAACTTTTGTCAWTTAAT		FISH probe
CEN-2	ATATTGGBACACGAGGGTGYGATSTTTTCTACTGGAATCA		FISH probe
CEN-3	ACATGMKTTTTGGACAWATWGGASTGTRTTGGGYG		FISH probe
