# Supplementary material for: ZEP1 orchestrates template choice and crossover pathways to ensure meiotic genome integrity in rice
Source: EMBO J. 2026 Sep 17;45(20):7163–90. doi: 10.1038/s44318-026-00910-0 (PMC13672304; doi:10.1038/s44318-026-00910-0)
Supplement: Supplementary file 2 [file 44318_2026_910_MOESM2_ESM.pdf]

THE EMBO JOURNAL

ZEP1 orchestrates template choice and crossover pathways to ensure meiotic genome integrity in rice

Jie zhang, Chong Wang, Yingzhu Xiao, Xiaofei Chen, James Higgins, and Wanqi Liang

Corresponding author(s): Wanqi Liang (wqliang@sjtu.edu.cn)

Review Timeline:

Submission Date:	22nd Mar 26
Editorial Decision:	4th May 26
Revision Received:	29th Jul 26
Editorial Decision:	26th Aug 26
Revision Received:	30th Aug 26
Accepted:	1st Sep 26

Editor: Hartmut Vodermaier

Transaction Report:

(Note: With the exception of the correction of typographical or spelling errors that could be a source of ambiguity, letters and reports are not edited. Depending on transfer agreements, referee reports obtained elsewhere may or may not be included in this compilation. Referee reports are anonymous unless the Referee chooses to sign their reports.)

Dr. Wanqi Liang
Shanghai Jiao Tong University
School of Life Science and Biotechnology
Shanghai
China

4th May 2026

Re: EMBOJ-2026-124191
ZEP1 orchestrates template choice and crossover pathways to ensure meiotic genome integrity in rice

Dear Dr Liang,

Thank you for submitting your study on meiotic functions of rice ZEP1 to The EMBO Journal. It has now been reviewed by three experts, whose comments are copied below for your information. All referees appreciate the potential interest of your work, but also raise several important points that would need to be adequately addressed before publication. In particular, all reviewers ask for more decisive analyses of how ZEP1 may function to counter recombination, and to coordinate class I and II crossover pathways.

Should you be able to satisfactorily respond to these various concerns, we would be happy to pursue a revised manuscript further for publication in our journal. Please remember, however, that it is our policy to consider only a single round of major revision, making it important to adequately answer all comments at the time of resubmission; therefore, please do not hesitate to get back to me in case you would like to clarify/discuss any of the referees' points or plans for addressing them ahead of time. We would also be open to extending the revision deadline if that should be helpful. As always, our scooping protection will remain valid throughout the full revision period.

Detailed information on preparing, formatting and uploading a revised manuscript can be found below and in our Guide to Authors. Thank you again for the opportunity to consider this work for The EMBO Journal, and I look forward to hearing from you in due time.

Yours sincerely,

Hartmut Vodermaier

Hartmut Vodermaier, PhD
Senior Editor, The EMBO Journal
h.vodermaier@embojournal.org

*** PLEASE NOTE: All revised manuscript are subject to initial checks for completeness and adherence to our formatting guidelines. Revisions may be returned to the authors and delayed in their editorial re-evaluation if they fail to comply to the following requirements. As a first step please read our guidelines for revised submissions:
https://link.springer.com/journal/44318/submission-guidelines#cms-Revised-submissions

1) Every manuscript requires a Data Availability section (even if only stating that no deposited datasets are included). Primary datasets or computer code produced in the current study have to be deposited in appropriate public repositories prior to resubmission, and reviewer access details provided in case that public access is not yet allowed.

2) Each figure legend must specify
- size of the scale bars that are mandatory for all micrograph panels
- the statistical test used to generate error bars and P-values
- the type error bars (e.g., S.E.M., S.D.)
- the number (n) and nature (biological or technical replicate) of independent experiments underlying each data point
- Figures may not include error bars for experiments with n<3; scatter plots showing individual data points should be used instead.

3) Revised manuscript text (including main tables, and figure legends for main and EV figures) has to be submitted as editable text file (e.g., .docx format). We encourage highlighting of changes (e.g., via text color) for the referees' reference.

4) Each main and each Expanded View (EV) figure should be uploaded as individual production-quality files (preferably in .eps,

.tif, .jpg formats). For suggestions on figure preparation/layout, please refer to our Figure Preparation Guidelines: https://media.springernature.com/original/springer-cms/rest/v1/content/27825798/data/v1

5) Point-by-point response letters should include the original referee comments in full together with your detailed responses to them (and to specific editor requests if applicable), and also be uploaded as editable (e.g., .docx) text files.

6) Please complete our Author Checklist, and make sure that information entered into the checklist is also reflected in the manuscript; the checklist will be available to readers as part of the Review Process File.

7) All authors listed as (co-)corresponding need to deposit, in their respective author profiles in our submission system, a unique ORCiD identifier linked to their name. Please see our Guide to Authors for detailed instructions.

8) Please note that supplementary information at EMBO Press has been superseded by the 'Expanded View' for inclusion of additional figures, tables, movies or datasets; with up to five EV Figures being typeset and directly accessible in the HTML version of the article.

9) To facilitate reproducibilty and cross-laboratory adoption of methodologies, please structure the Materials & Methods section as outlined in our guide to authors, including a completed Reagents and Tools Table.

10) Digital image enhancement is acceptable practice, as long as it accurately represents the original data and conforms to community standards. If a figure has been subjected to significant electronic manipulation, this must be clearly noted in the figure legend and/or the 'Materials and Methods' section. The editors reserve the right to request original versions of figures and the original images that were used to assemble the figure. Finally, we generally encourage uploading of numerical as well as gel/blot image source data.

At EMBO Press we ask authors to provide source data for the main manuscript figures. You will receive a separate email with instructions for providing source data with your revised manuscript, including how to upload and organize the files.

In the interest of ensuring the conceptual advance provided by the work, we recommend submitting a revision within 3 months (2nd Aug 2026). Please discuss the revision progress ahead of this time with the editor if you require more time to complete the revisions. Use the link below to submit your revision:

Link Not Available

Referee #1:

In their study, Zhang et al. investigated the effects of removing the transverse filament protein ZEP1 on meiosis in rice, with a particular focus on meiotic recombination. The role of the synaptonemal complex (SC) in crossover recombination has been widely studied in recent years, following the discovery of a key role of the SC in controlling crossover assurance and interference in Arabidopsis, as well as the identification of new SC components in Arabidopsis. Moreover, previous work in rice relied on a hypomorphic zep1 allele, creating a clear need to compare that phenotype with the null alleles that are now available.
Overall, the manuscript is well thought out, supported by an extensive dataset, and contains several important findings that significantly advance our understanding of SC function in plant meiotic recombination, while also highlighting differences between Arabidopsis and crop species. The authors show that loss of the SC due to ZEP1 disruption increases multivalents in male meiosis, suggesting that this protein suppresses interactions between non-homologous chromosomes. In contrast, they do not observe the phenotype typical of the hypomorphic zep1 mutant, in which class I crossovers increase substantially.
The study includes a large number of genetic combinations with mutants affecting different steps of meiotic recombination, although some of these combinations appear somewhat excessive (without sufficient justification; see below). The genetic analyses are complemented by evidence for interactions between ZEP1 and selected recombination proteins, which helps to better support a mechanistic model for how ZEP1 influences DSB repair pathway choice.

Major comments
1. One of the main conclusions of the paper is that ZEP1 coordinates crossover formation by affecting the activity of anti-recombination pathways. In this context, the authors examined genetic and biochemical interactions with MEICA1, FIGNL1, and RMI1. However, they did not test interactions with the most powerful anti-recombination factors, namely the DNA helicases FANCM and RECQ4. Inactivation of either enzyme leads to a strong increase in CO frequency in rice (Mieulet et al., Nat Plants 2018), and recently published analyses in Arabidopsis show even stronger effects of RECQ4 loss when combined with zyp1 (Jing et al., Nat Commun 2025). The manuscript would benefit substantially-particularly by strengthening the conclusion that the SC regulates anti-recombination pathways-if interactions with helicase mutants, especially recq4, were included. The effect of RMI1 alone is typically much smaller than that of the full RECQ4-RMI1-TOP3α complex; therefore, the lack of a recq4

combination is a limitation. I do recognize, however, that the authors may wish to reserve these interactions for a future study.
2. It would be very interesting to determine how the CO distribution along chromosomes changes in the zep1 null background relative to WT or zep1 hypomorph, ideally by generating genome-wide CO maps. This would allow the authors to more broadly assess CO interference and to quantify more precisely to what extent class II COs increase.
3. In light of current knowledge about meiosis, the rationale for testing genetic interactions between ZEP1 and SDS, CRC1, or Ku70 is not obvious. The authors should better justify why these particular analyses were performed.
4. Some conclusions are stated too strongly relative to the data and should be mitigated. For example, line 309 ("These results indicate that ZEP1 acts after strand invasion during HR-mediated DSB repair...") is based solely on comparing chromosome configurations in prophase I, which is certainly not the only relevant phenotypic readout. I suggest using more cautious wording (e.g., "suggest").

Minor comments
1. Line 244: Figures 3 and 4 should be cited, not only Figure 3. The authors could also consider merging these two figures.
2. I could not find a reference to Appendix Fig. S7.
3. Lines 297-303: it is not clear what "n" refers to here. If this is simply sample size, it is not very informative and may even be confusing-especially since the values do not match the numbers indicated in Fig. 3. The numbers of univalents/bivalents (or other relevant configurations) should be indicated instead.
4. Lines 387-389: "complete loss of MEICA1 in the zep1 background further compromises CO assurance..." - crossover assurance is a qualitative property (it is either present or absent). Since CO assurance is already lost in zep1, the authors should rather refer to a further reduction in CO number.
5. Fig. 7 caption and line 534: the authors use the abbreviation STR, which refers to the yeast complex (with different protein names than in plants). Since the Introduction defines yeast STR as homologous to the plant RTR complex, the manuscript should consistently use RTR in this context.

Referee #2:

This manuscript presents an extensive genetic and cytological analysis of rice zep1 null mutant series, across multiple genetic backgrounds. The authors provide a detailed cytological characterization of these mutants, including DAPI staining, bivalent analysis, and quantification of HEI10 foci from pachytene to diplotene, alongside substantial genetic interaction data. They also present protein interaction data that could offer interesting insights into the function of ZEP1 in rice.
Overall, the dataset is rich, carefully quantified, and generally clearly presented. A key strength of the study lies in the comprehensive characterization of zep1 null phenotypes. The authors convincingly demonstrate that the primary defect is related to DSB repair, while also ruling out a general increase in CO formation. The breadth of genetic analyses provides robust and consistent results that support the main conclusions. In addition, the manuscript contributes to a deeper understanding of the role of ZEP1 in meiotic recombination, particularly in relation to anti-recombination pathways. This aspect represents the main source of novelty in the study, even if this part of the study could benefit from some improvements (see below).
Points to address:
• Interpretation of HEI10 foci as crossovers.
The authors equate HEI10 foci with class I COs, which is not sufficiently supported without direct genetic recombination measurements. Given the presence of substantial chromosome fragmentation, it is likely that some (most) HEI10 foci do not correspond to mature COs.
• The paragraph title "ZEP1 suppresses interactions between non-homologous chromosomes" is misleading (L177). It does not fully reflect the content of the paragraph
• The description of brca2 mutants (asynapsis, entanglements, fragmentation) closely resembles the zep1 phenotype. The manuscript should more clearly define the distinguishing features between these mutants.
• In the genetic interactions section. While the data are solid, the language used is often informal or imprecise. Phrases such as: "closely resembled", "shifted toward the dmc1 phenotype", "chromosome associations" , "toxic chromosome associations" are vague and should be replaced with more precise, quantitative descriptions.
• Protein interaction data
The large number of reported interactions with ZEP1 raises concerns about specificity and potential nonspecific binding. It would be important to clarify:
- Which interactions were tested but not confirmed by BiFC or co-IP
- Whether controls have been performed to validate the negative interactions (are the fusion proteins expressed correctly).
- For the BiFC experiments, the subcellular localization should be described more precisely (nuclear? speckled? aggregates?).
• ZEP1 and anti-recombinase interactions (MEICA1/FIGNL1/RMI1)
This is one of the most promising aspects of the manuscript but requires further strengthening:
- DMC1 quantification. The criteria used to stage meiocytes should be clearly explained. Additionally, does RAD51 exhibit a similar delay in signal disappearance as DMC1?
- Antibody validation for MEICA1, FIGLN1 and RMI1 is insufficient. Only one control is presented (for RMI1, Fig S16), and detection of a band on a western blot at the expected size is not sufficient; specificity must be demonstrated using mutant controls.
- The localization patterns of MEICA1, FIGNL1, and RMI1 in wild type are not described in sufficient detail. Immunofluorescence analyses with precise staging (e.g., using ZEP1 or HEI10 as markers) should be provided. It should also be clarified whether

these proteins associate with chromosome axes or the transverse filaments, depending on the stage.
- The relationship is only analyzed in the zep1 background, not reciprocally.
- Temporal aspects of recombination regulation. A key conceptual issue remains unresolved: MEICA1, FIGNL1, and RMI1 are reported to act early in recombination, prior to synapsis, and therefore before ZEP1 is loaded. The manuscript does not adequately discuss how ZEP1 could regulate strand invasion at that stage or be involved in the loading of these factors onto the chromosome axis while not yet being present itself. Possible explanations (e.g., early non-polymerized ZEP1 or non-canonical roles) should be discussed.

Referee #3:

In this manuscript, Zhang et al. investigate the function of the rice synaptonemal complex transverse filament protein ZEP1 during meiosis. The authors propose that ZEP1 functions beyond SC assembly and class I crossover (CO) promotion, acting instead as a regulatory coordinator of recombination template choice and crossover pathway balance. They further suggest that ZEP1 acts after strand invasion, promotes the proper chromosomal association of anti-crossover factors including MEICA1, FIGNL1, and RMI1, and thereby channels recombination intermediates toward productive outcomes while limiting aberrant chromosome interactions and genome instability.
This is an important topic, and the manuscript will be of broad interest to the meiosis and chromosome biology communities. The conceptual advance proposed here-that a transverse filament protein functions not only as a structural component of the synaptonemal complex but also as an active regulator of recombination intermediate fate-is both interesting and potentially significant. The study combines cytology, genetics, protein interaction analyses, and comparisons across multiple rice backgrounds, which represents a notable strength. Overall, the manuscript is well organized and clearly written, and the data appear to be of high quality.
My main concerns relate to the evidence supporting the proposed coordination of class I versus class II CO pathways, and to the interpretation of the background-dependent effects observed in different rice genetic backgrounds.
Major points
1. Evidence for coordination of class I and class II crossover pathways
A major conclusion of this study is that ZEP1 plays an important role in coordinating class I and class II CO pathways. The authors provide evidence that ZEP1 directly interacts with anti-CO factors and is required for their proper chromosomal association, supporting a model in which ZEP1 helps prevent excessive strand invasion and limits inappropriate recombination outcomes that may otherwise favor class II CO formation. This is an interesting and well-supported aspect of the study. However, if the authors wish to argue that ZEP1 coordinates the balance between both class I and class II pathways, it would be important to further clarify whether ZEP1 also has direct or functional connections with class I CO factors. At present, the evidence appears stronger for a role in restraining aberrant or excess recombination through anti-CO machinery than for a direct role in coordinating both pathways symmetrically. If additional interaction, localization, or genetic evidence regarding class I CO factors is available, inclusion of such data would substantially strengthen this conclusion. Otherwise, the authors may wish to frame this point somewhat more cautiously in the text.
2. Interpretation of the background-dependent effects
The comparison across japonica, indica, and hybrid backgrounds is potentially valuable and may reveal that SC-dependent recombination outcomes are highly sensitive to genome context. This is an interesting aspect of the manuscript. However, the mechanistic interpretation currently appears to go somewhat beyond the data presented. In particular, statements suggesting that ZEP1 is embedded in an "evolving recombination network" are plausible, but at present remain interpretive unless supported by more direct comparative genetic or molecular evidence.
This section would benefit from a tighter linkage between the observed phenotypic differences and the conclusions drawn from them. In particular, the Discussion should more clearly acknowledge the limitation that the basis of these background-dependent effects remains unresolved. A more measured discussion of this point would improve the balance of the manuscript.
Minor points
1. Lines 321-322: "In rice, zmm mutants complete synapsis but form many univalents at metaphase I." The data shown in Figure 4 appear to be consistent with this statement. However, statistical evaluation of chiasma number would be helpful to further support this conclusion and to make the comparison more rigorous.
2. Lines 375-376 and 403-404: Figure S13 and Figure S14 show defective loading patterns of MEICA1/FIGNL1 and RMI1 in zep1 null-2, similar to those observed in zep1 null-1. It would be helpful if the authors could provide quantitative estimates of the proportion of cells showing reduced signals, as well as the proportion showing clear signal mislocalization. Such quantification would strengthen the conclusion that the phenotype is reproducible across independent null alleles.

Referee #1:

In their study, Zhang et al. investigated the effects of removing the transverse filament protein ZEP1 on meiosis in rice, with a particular focus on meiotic recombination. The role of the synaptonemal complex (SC) in crossover recombination has been widely studied in recent years, following the discovery of a key role of the SC in controlling crossover assurance and interference in Arabidopsis, as well as the identification of new SC components in Arabidopsis. Moreover, previous work in rice relied on a hypomorphic zep1 allele, creating a clear need to compare that phenotype with the null alleles that are now available.
Overall, the manuscript is well thought out, supported by an extensive dataset, and contains several important findings that significantly advance our understanding of SC function in plant meiotic recombination, while also highlighting differences between Arabidopsis and crop species. The authors show that loss of the SC due to ZEP1 disruption increases multivalents in male meiosis, suggesting that this protein suppresses interactions between non-homologous chromosomes. In contrast, they do not observe the phenotype typical of the hypomorphic zep1 mutant, in which class I crossovers increase substantially.
The study includes a large number of genetic combinations with mutants affecting different steps of meiotic recombination, although some of these combinations appear somewhat excessive (without sufficient justification; see below). The genetic analyses are complemented by evidence for interactions between ZEP1 and selected recombination proteins, which helps to better support a mechanistic model for how ZEP1 influences DSB repair pathway choice.
Response: We thank the Referee for the positive assessment of our study, and the insightful comments and suggestions, which have helped us improve the focus and overall quality of our manuscript.

Major comments
1. One of the main conclusions of the paper is that ZEP1 coordinates crossover formation by affecting the activity of anti-recombination pathways. In this context, the authors examined genetic and biochemical interactions with MEICA1, FIGNL1, and RMI1. However, they did not test interactions with the most powerful anti-recombination factors, namely the DNA helicases FANCM and RECQ4. Inactivation of either enzyme leads to a strong increase in CO frequency in rice (Mieulet et al., Nat Plants 2018), and recently published analyses in Arabidopsis show even stronger effects of RECQ4 loss when combined with zyp1 (Jing et al., Nat Commun 2025). The manuscript would benefit substantially-particularly by strengthening the conclusion that the SC regulates anti-recombination pathways-if interactions with helicase mutants, especially recq4, were included. The effect of RMI1 alone is typically much smaller than that of the full RECQ4-RMI1-TOP3α complex; therefore, the lack of a recq4 combination is a limitation. I do recognize, however, that the authors may wish to reserve these interactions for a future study.
Response: We thank the Referee for this important and constructive suggestion.

We agree that FANCM and RECQ4 are major anti-crossover helicases in plants and that genetic interaction analyses between *zep1* and *recq4/fancm* would be highly informative, particularly for further dissecting the relationship between the SC and anti-recombination pathways.

In our original submission, we tested whether ZEP1 physically interacts with FANCM or RECQ4 using Y2H assay, but did not detect an interaction between ZEP1 and either helicase (original Appendix Figure S12). In the revised manuscript, we further examined these interactions using BiFC assays and again detected no apparent interaction between ZEP1 and either protein (Appendix Figure S10B in the revised manuscript). Taken together, these results suggest that ZEP1 is unlikely to regulate faithful HR repair through direct interactions with FANCM or RECQ4.

Regarding RECQ4, we agree that RMI1 alone may not fully represent the activity of the complete RTR complex. Nevertheless, several observations led us to prioritize RMI1 in the present study. The RTR complex forms a stable ternary complex in both *Arabidopsis* (PMID: 27965412) and rice (PMID: 40517397), and recent genetic evidence suggests that RMI1 has a particularly prominent role in faithful meiotic DSB repair in rice. Even hypomorphic *rmi1* mutants exhibit obvious meiotic abnormalities (PMID: 40517397; this study), whereas *recq4* null mutant complete meiosis without obvious cytological defects (PMID: 30478361; PMID: 40517397), despite their elevated CO frequency. Therefore, our finding that ZEP1 physically interacts with RMI1 and is required for its normal chromosomal association supports the idea that ZEP1 may influence RTR-associated repair functions by recruiting or stabilizing RMI1 within the SC environment. We have therefore clarified this point in the revised manuscript and now emphasize the potential regulatory impact of ZEP1 on the RMI1-dependent functional subset of the RTR complex. Please see Lines 508–516 and Figure 7 legend (Line 1179)

We fully agree that generating and analyzing *zep1 recq4* and *zep1 fancm* double mutants would provide a valuable extension of this work, especially for assessing crossover frequency and repair outcomes in the absence of these major helicases. However, producing these genetic materials and completing the required cytological and recombination analyses would require substantial additional time, likely at least one year. We therefore are unable to include these data in the current revision. To address this limitation, we have added a statement in the revised Discussion acknowledging that the genetic relationships between ZEP1 and FANCM/RECQ4 remain to be tested in future studies. Please see Lines 516-518.

2. It would be very interesting to determine how the CO distribution along chromosomes changes in the zep1 null background relative to WT or zep1 hypomorph, ideally by generating genome-wide CO maps. This would allow the authors to more broadly assess CO interference and to quantify more precisely to what extent class II COs increase.

Response: We fully agree with the Referee that comparing genome-wide CO

landscapes among *zep1* null mutants, WT, and the previously reported *zep1* hypomorphic allele would be highly informative. Such analyses would provide a powerful way to assess changes in CO distribution, CO interference, and the relative contribution of class II COs.

As the Referee notes, genome-wide CO mapping has been successfully applied in *Arabidopsis zyp1* mutants and related genetic backgrounds using gamete transmission approaches, because these mutants still produce sufficient viable gametes for recovery and sequencing (e.g., PMID: 33782125; PMID: 33723072; PMID: 40506457). Similarly, in rice, a previous study reported increased recombination frequency on a specific chromosome in a highly fertile *zep1* hypomorphic allele (PMID: 25936677). In contrast, the *zep1* null alleles analyzed in our study display severe male meiotic defects, including extensive multivalent formation, chromosome fragmentation, aneuploid gamete production, and near-complete male sterility. These phenotypes make it technically difficult to generate reliable genome-wide CO maps based on male gamete transmission.

Given these biological and technical constraints, we instead assessed CO changes at the cytological level by monitoring CO marker protein foci (Figure 2A and Appendix Figure S6 in revised manuscript) and by analyzing *zep1* in combination with mutants affecting CO-regulatory pathways (Figure 3B). We hope that this explanation adequately addresses the Referee's concerns.

3. In light of current knowledge about meiosis, the rationale for testing genetic interactions between ZEP1 and SDS, CRC1, or Ku70 is not obvious. The authors should better justify why these particular analyses were performed.

Response: Thanks for your comment. In rice, SDS and CRC1 are required for DSB formation (PMID: 25691887; PMID: 23943860). Because *zep1* null mutants exhibit severe and atypical meiotic defects, including extensive multivalent formation and chromosome fragmentation, we used *sds* and *crc1* backgrounds to determine whether these abnormalities depend on programmed meiotic DSB formation. These analyses allowed us to distinguish whether the chromosome defects in *zep1* arise as a consequence of defective meiotic DSB repair, rather than simply from loss of synapsis or SC structure. We have added this rationale to the revised manuscript to make the logic of these genetic analyses clearer. Please see Lines 239-249 in the revised manuscript.

We initially analyzed KU70 because KU70 is a core component of the canonical non-homologous end-joining pathway and has been shown to antagonize homologous recombination repair in plants (PMID: 19923234; PMID: 23050791). In rice meiosis, *ku70*-based double-mutant analyses have been widely used to investigate the contribution of non-homologous interactions to meiotic chromosome abnormalities (PMID: 27512017; PMID: 32275774; PMID: 28696221; PMID: 35898226). Therefore, the *zep1 ku70* analysis was designed to test whether the multivalents and chromosome fragmentation observed in *zep1* mutants are mediated by KU70-dependent end-joining/non-homologous repair, or instead arise primarily from aberrant HR-mediated recombination. Quite recently,

however, KU70-mediated non-homologous end joining has been challenged by new evidence suggesting that it does not serve as a major compensatory pathway when meiotic homologous recombination is compromised (PMID: 42076849). Given that the *zep1* phenotype does not depend on the KU70-mediated pathway and that the contribution of this pathway to meiotic recombination repair remains under debate, we have removed the related description and discussion from the revised manuscript. This change helps focus the manuscript on the regulatory role of ZEP1 in DSB repair during meiotic homologous recombination pathway and does not affect the interpretation of our findings.

4. Some conclusions are stated too strongly relative to the data and should be mitigated. For example, line 309 ("These results indicate that ZEP1 acts after strand invasion during HR-mediated DSB repair...") is based solely on comparing chromosome configurations in prophase I, which is certainly not the only relevant phenotypic readout. I suggest using more cautious wording (e.g., "suggest").

Response: Thanks for your correction. We have revised the text accordingly and adopted more cautious wording. Please see Line 287 in the revised manuscript. In addition, we have carefully revised other statements involving interpretation of our data (e.g., Lines 514–516, 574-577) to better reflect the evidence provided. The corresponding changes are highlighted in red in the revised manuscript.

Minor comments

1. Line 244: Figures 3 and 4 should be cited, not only Figure 3. The authors could also consider merging these two figures.

Response: As suggested, we have combined Figures 3 and 4 into a single figure and have carefully checked all corresponding Figure citations throughout the manuscript. In the revised manuscript, the combined figure is presented as Figure 3, and several supporting results have been relocated to the Extended View Figure (Figure EV2A) due to main figure space constraints.

2. I could not find a reference to Appendix Fig. S7.

Response: Appendix Fig. S7 was cited in the originally submitted manuscript at Line 261. In the revised version, the appendix Figure has been renumbered as Appendix Figure S8, and we have retained its citation, which now appears on Lines 228 and 578-579 of the revised manuscript.

3. Lines 297-303: it is not clear what "n" refers to here. If this is simply sample size, it is not very informative and may even be confusing-especially since the values do not match the numbers indicated in Fig. 3. The numbers of univalents/bivalents (or other relevant configurations) should be indicated instead.

Response: Thanks for your correction. "n" denotes the number of meiotic cells at the indicated stage included in each statistical analysis (e.g., total metaphase I cell count for statistics in Lines 297–303 of the previously submitted manuscript, whereas the "n" in Fig. 3 denotes total anaphase I cells used for statistical analysis).

More explicit information regarding sample numbers and statistical procedures has been included in the corresponding Figure legend (e.g., Lines 1092-1107 in revised manuscript). As suggested by the Referee, we have also provided the corresponding numbers of chromosome configurations in the revised manuscript please see Lines 254-260 and 268-276.

4. Lines 387-389: "complete loss of MEICA1 in the zep1 background further compromises CO assurance..." - crossover assurance is a qualitative property (it is either present or absent). Since CO assurance is already lost in zep1, the authors should rather refer to a further reduction in CO number.

Response: We thank the Referee for this helpful clarification. We have revised the relevant sentence accordingly. Please see Lines 385-386 in the revised manuscript.

5. Fig. 7 caption and line 534: the authors use the abbreviation STR, which refers to the yeast complex (with different protein names than in plants). Since the Introduction defines yeast STR as homologous to the plant RTR complex, the manuscript should consistently use RTR in this context.

Response: We thank the Referee for the careful review. We have corrected the terminology and now consistently use RTR when referring to the plant complex throughout the manuscript. Please see Line 514 and Figure 7 legend (Line 1175) in the revised manuscript.

Referee #2:

This manuscript presents an extensive genetic and cytological analysis of rice zep1 null mutant series, across multiple genetic backgrounds. The authors provide a detailed cytological characterization of these mutants, including DAPI staining, bivalent analysis, and quantification of HEI10 foci from pachytene to diplotene, alongside substantial genetic interaction data. They also present protein interaction data that could offer interesting insights into the function of ZEP1 in rice.

Overall, the dataset is rich, carefully quantified, and generally clearly presented. A key strength of the study lies in the comprehensive characterization of zep1 null phenotypes. The authors convincingly demonstrate that the primary defect is related to DSB repair, while also ruling out a general increase in CO formation. The breadth of genetic analyses provides robust and consistent results that support the main conclusions. In addition, the manuscript contributes to a deeper understanding of the role of ZEP1 in meiotic recombination, particularly in relation to anti-recombination pathways. This aspect represents the main source of novelty in the study, even if this part of the study could benefit from some improvements (see below).

Response: We thank the Referee for the positive assessment of our work and for recognizing the significance of the dataset and findings. We appreciate the constructive comments and suggestions, which have been very helpful in improving the clarity, interpretation, and presentation of the manuscript.

Points to address:

• Interpretation of HEI10 foci as crossovers.

The authors equate HEI10 foci with class I COs, which is not sufficiently supported without direct genetic recombination measurements. Given the presence of substantial chromosome fragmentation, it is likely that some (most) HEI10 foci do not correspond to mature COs.

Response: We thank the Referee for this important point. We agree that HEI10 foci should not be interpreted unconditionally as mature COs, particularly in mutant backgrounds with substantial chromosome fragmentation, where some HEI10 foci may fail to mature into functional COs.

At the same time, HEI10 is widely used as a cytological marker for class I CO designation in plants and, to our knowledge, remains the most informative available marker for this process in rice. To strengthen our interpretation, we examined the dynamics of HEI10 foci throughout prophase I using different meiocyte fixation methods (Figure 2 and Appendix Figure S6 in revised manuscript). These analyses showed that, in late prophase I, both the number and fluorescence intensity of HEI10 foci gradually reach a relatively stable state. We therefore interpret these late-stage HEI10 foci as being enriched for sites that have progressed toward class I CO maturation.

Consistent with this interpretation, the abundance of late-stage HEI10 foci shows a negative correlation with univalent frequency across *zep1* mutants in diverse rice genetic backgrounds (Figure 2B and Figure 3B). Therefore, our

cytological data support the conclusion that loss of ZEP1 affects the progression and/or maturation of HEI10-marked class I recombination intermediates.

• The paragraph title "ZEP1 suppresses interactions between non-homologous chromosomes" is misleading (L177). It does not fully reflect the content of the paragraph

Response: Thanks for your correction. We have revised the sentence accordingly. Please see Line 161-162 in the revised manuscript.

• The description of brca2 mutants (asynapsis, entanglements, fragmentation) closely resembles the zep1 phenotype. The manuscript should more clearly define the distinguishing features between these mutants.

Response: Thanks for your suggestion. We have added a more detailed description accordingly. Please see Lines 254-260, 265-276 and 284-285 in the revised manuscript.

• In the genetic interactions section. While the data are solid, the language used is often informal or imprecise. Phrases such as: "closely resembled", "shifted toward the dmc1 phenotype", "chromosome associations", "toxic chromosome associations" are vague and should be replaced with more precise, quantitative descriptions.

Response: We thank the Referee for pointing this out. We have revised the description accordingly. Please see Lines 112, 254-260, 265-276, 284-285, 296, 299, 304, 346, 377, 426, 527, 564, 573 and Figure legends (Lines 1034, 1143, 1157) in the revised manuscript.

• Protein interaction data

The large number of reported interactions with ZEP1 raises concerns about specificity and potential nonspecific binding. It would be important to clarify:

- Which interactions were tested but not confirmed by BiFC or co-IP

Response: Thank you for your comment. We tested all protein combinations that were positive in the Y2H assays using BiFC. Overall, the interactions obtained detected by the Y2H assays were consistently confirmed by the BiFC. In addition, BiFC detected interactions between ZEP1 and several strand-invasion proteins, including DMC1, RAD51A, RAD51C, and XRCC3, as well as CO-regulating proteins PTD, HEI10, and GEN1. These results are shown in Appendix Figure S10B in the revised manuscript.

We also recognize that BiFC is highly sensitive and that fluorescence complementation is essentially irreversible, which can allow detection of transient or weak interactions but may also increase the need for cautious interpretation (PMID: 25772494). Therefore, in the revised manuscript, we place greater emphasis on interactions supported by more than one assay, especially those consistently detected by both Y2H and BiFC. We have revised the text to clarify this point and to describe the validation strategy more explicitly. Please see Lines 329-331 and 333-344 in the revised manuscript.

- Whether controls have been performed to validate the negative interactions (are the fusion proteins expressed correctly).

Response: Thank you for raising this important point. In our assays, negative results in the Y2H system were not used as definitive evidence that two proteins cannot interact. Indeed, as noted above, several interactions detected by BiFC were not identified in the Y2H assays, indicating that Y2H may fail to detect some interactions, possibly due to protein folding, localization, fusion orientation, or interaction strength.

Regarding construct functionality, several proteins that did not show interaction with ZEP1 in Y2H have been shown to interact with other meiotic proteins in our previous studies using the same or comparable interaction systems, supporting that these fusion constructs are functional and suitable for protein-interaction assays (PMID: 31266799; PMID: 33304374).

- For the BiFC experiments, the subcellular localization should be described more precisely (nuclear? speckled? aggregates?).

Response: Thank you for the suggestion. When transiently expressed outside the meiotic context, ZEP1 showed a predominantly nuclear, punctate localization pattern in tobacco leaves and rice protoplasts (see figure below). The BiFC fluorescence signals for ZEP1-associated interactions were similarly observed mainly as punctate nuclear signals, rather than diffuse fluorescence signals. A similar localization pattern has also been reported for the *Caenorhabditis elegans* TF protein upon heterologous expression in non-meiotic cells (PMID: 32211900). We have clarified the subcellular localization in the revised manuscript, including in the legends of Figure 4D (Lines 1126-1127) and Appendix Figure S10B.

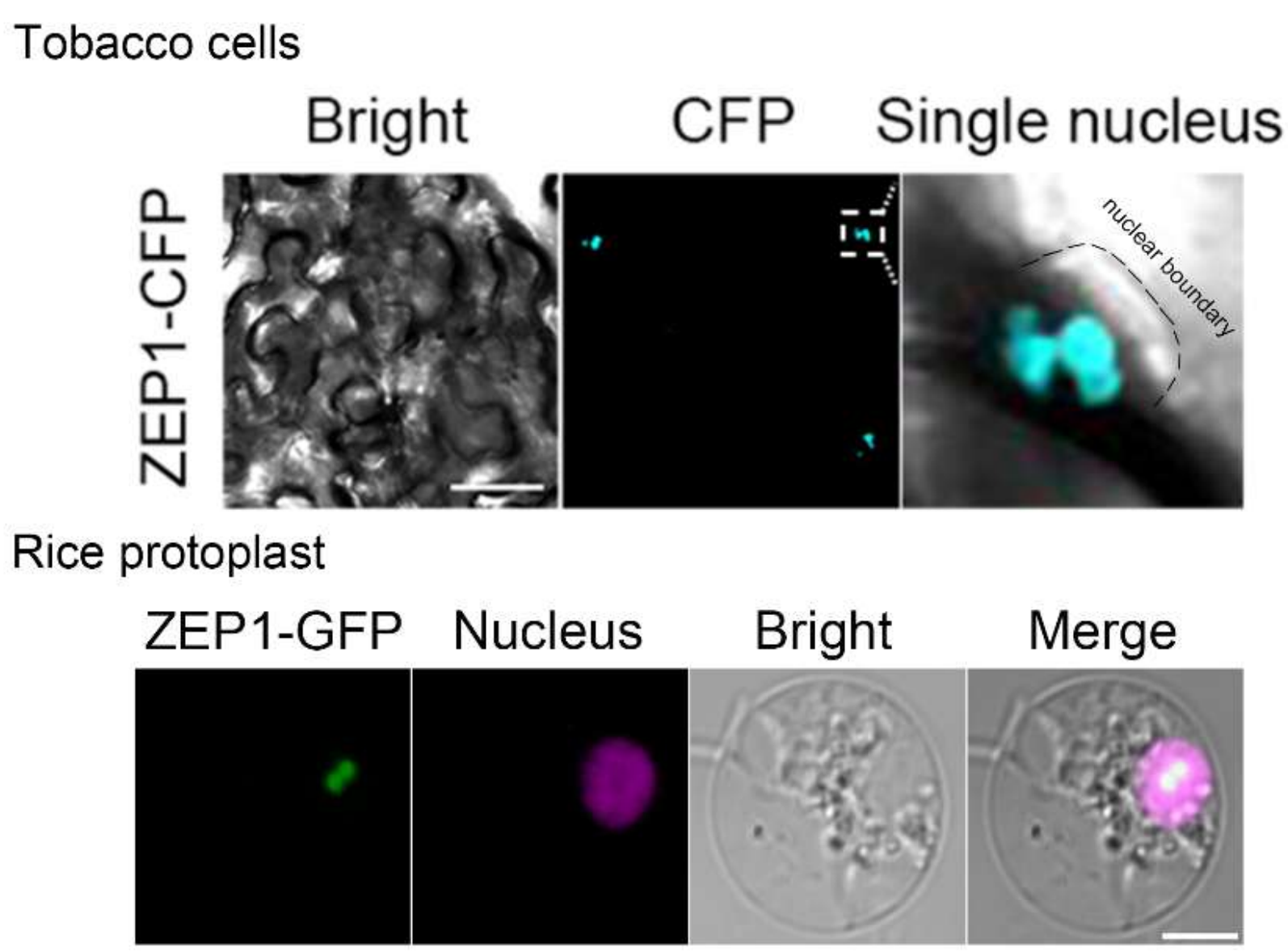

• ZEP1 and anti-recombinase interactions (MEICA1/FIGNL1/RMI1)

This is one of the most promising aspects of the manuscript but requires further

strengthening:

- DMC1 quantification. The criteria used to stage meiocytes should be clearly explained. Additionally, does RAD51 exhibit a similar delay in signal disappearance as DMC1?

Response: Thank you for your helpful suggestion. Although *zep1* mutants exhibit synapsis defects, our data demonstrate that homologous chromosome pairing remains unaffected, allowing reliable identification of the leptotene and zygotene stages. At the pachytene-equivalent stage, despite the absence of synapsis, homologous chromosomes undergo characteristic condensation and display distinct morphological characteristics that clearly distinguish this stage from the other stages of meiosis. We now present representative images of consecutive meiotic stages in the revised manuscript to facilitate stage identification.

Consistent with the delayed removal of DMC1, RAD51 also exhibits delayed dissociation from meiotic chromosomes in *zep1* mutant. We have now included representative RAD51 images and quantification across consecutive meiotic stages in the revised manuscript. These data support the conclusion that loss of ZEP1 leads to prolonged chromosome association of strand-invasion recombinases. Please see Figure 5D, Figure EV4B-D and Lines 23, 367-376 in the revised manuscript.

- Antibody validation for MEICA1, FIGLN1 and RMI1 is insufficient. Only one control is presented (for RMI1, Fig S16), and detection of a band on a western blot at the expected size is not sufficient; specificity must be demonstrated using mutant controls.

Response: Thank you for your helpful suggestion. For MEICA1 and FIGNL1, antibody specificity was validated using the *meica1* and *fignl1* null mutants in the originally submitted manuscript. These validation data were shown in Appendix Figure S13, which has now been renumbered as Appendix Figure S11 in the revised manuscript.

For RMI1, however, a comparable null-mutant validation is not feasible because complete loss of RMI1 results in embryonic lethality, as shown in this study and also reported recently (PMID: 39569466). We therefore used alternative evidence to assess the specificity of the RMI1 antibody. The RMI1 antibody produces chromosomal signals that are specific to prophase I and are recombination-dependent, which is consistent with the expected biological behavior of RMI1 during meiosis (Figure 6A of the revised manuscript). These cytological features provide additional support for the specificity of the RMI1 antibody, although we acknowledge that they are not equivalent to validation using a null mutant.

- The localization patterns of MEICA1, FIGNL1, and RMI1 in wild type are not described in sufficient detail. Immunofluorescence analyses with precise staging (e.g., using ZEP1 or HEI10 as markers) should be provided. It should also be clarified whether these proteins associate with chromosome axes or the transverse filaments, depending on the stage.

Response: Thanks for your helpful suggestion. We performed super-resolution

imaging to examine the spatial localization pattern of MEICA1, FIGNL1, and RMI1 relative to the chromosome axis marker REC8 and transverse filament ZEP1 in zygotene and pachytene meiocytes. Our observations suggest that these three proteins are progressively redistributed toward the synaptonemal complex, where ZEP1 is localized. A detailed description has been added to the revised manuscript. Please see Figure 5A-B, Figure 6B-C, Figure EV3 and Lines 351-358 and 398-401.

- The relationship is only analyzed in the zep1 background, not reciprocally.

Response: Thanks for your suggestion. We have added the corresponding results and description to the revised manuscript. Please see Figure EV4A and Lines 363-366 and 404-406.

- Temporal aspects of recombination regulation. A key conceptual issue remains unresolved: The manuscript does not adequately discuss how ZEP1 could regulate strand invasion at that stage or be involved in the loading of these factors onto the chromosome axis while not yet being present itself. Possible explanations (e.g., early non-polymerized ZEP1 or non-canonical roles) should be discussed.

Response: Thank you for this helpful suggestion. We agree that the temporal relationship between ZEP1 assembly and the regulation of strand-invasion-associated factors needed to be addressed more explicitly.

To clarify this issue, we performed STED imaging to examine the spatiotemporal relationship between MEICA1/FIGNL1 and ZEP1 during meiotic prophase I. These analyses showed that, concomitant with the progressive polymerization of ZEP1 from zygotene to pachytene, MEICA1 and FIGNL1 signals gradually became enriched within the interaxial space between homologous chromosomes, corresponding to the canonical localization domain of polymerized ZEP1. In contrast, we did not observe an obvious spatial association between MEICA1/FIGNL1 and non-polymerized or punctate ZEP1 signals (Figure 5A-B). These observations suggest that ZEP1-dependent regulation of MEICA1/FIGNL1 occurs primarily concomitantly with SC assembly, rather than during the earliest strand-invasion events that precede detectable ZEP1 polymerization.

We now acknowledge more clearly that pre-SC strand invasion is widely recognized across species as a critical step for homolog recognition, homolog pairing, and synapsis initiation (PMID: 41399232; PMID: 25986558). However, based on our genetic analyses, protein-interaction assays, co-localization studies, and localization-dependency experiments, we propose that rice ZEP1/SC has an additional role beyond simply establishing an interhomolog axial scaffold. Specifically, ZEP1 may act as a regulatory platform during the transition from early strand-invasion intermediates to more mature recombination structures. By recruiting or stabilizing MEICA1–FIGNL1 and related anti-CO factors within the interhomolog region after SC assembly initiation, ZEP1 may help ensure the proper processing of strand-invasion-derived recombination intermediates,

promote productive template choice, and prevent the persistence of inappropriate or unstable chromosome interactions.

Accordingly, in the absence of ZEP1, early strand invasion may still occur, but the subsequent regulation and resolution of strand-invasion-derived intermediates may be impaired. This could allow improperly processed recombination intermediates to persist or accumulate, contributing to abnormal chromosome associations and chromosome fragmentation. We have therefore revised the manuscript to present ZEP1/SC as a potential post-strand-invasion "quality-control platform" that regulates the fate of recombination intermediates after initial homologous engagement, rather than as a factor required for the earliest strand-invasion step.

We have incorporated this more detailed discussion of the spatiotemporal regulation of anti-CO proteins by ZEP1 in the revised manuscript. Please see Lines 22-23, 26–28, 115–117, 386-389, 480-482, 485-504, 534-546 and Figure 7 (work model: Lines 1169-1174).

Referee #3:

In this manuscript, Zhang et al. investigate the function of the rice synaptonemal complex transverse filament protein ZEP1 during meiosis. The authors propose that ZEP1 functions beyond SC assembly and class I crossover (CO) promotion, acting instead as a regulatory coordinator of recombination template choice and crossover pathway balance. They further suggest that ZEP1 acts after strand invasion, promotes the proper chromosomal association of anti-crossover factors including MEICA1, FIGNL1, and RMI1, and thereby channels recombination intermediates toward productive outcomes while limiting aberrant chromosome interactions and genome instability.

This is an important topic, and the manuscript will be of broad interest to the meiosis and chromosome biology communities. The conceptual advance proposed here-that a transverse filament protein functions not only as a structural component of the synaptonemal complex but also as an active regulator of recombination intermediate fate-is both interesting and potentially significant. The study combines cytology, genetics, protein interaction analyses, and comparisons across multiple rice backgrounds, which represents a notable strength. Overall, the manuscript is well organized and clearly written, and the data appear to be of high quality.

My main concerns relate to the evidence supporting the proposed coordination of class I versus class II CO pathways, and to the interpretation of the background-dependent effects observed in different rice genetic backgrounds.

Response: We thank the Referee for the positive evaluation of our work and for recognizing the potential significance of our study. We have carefully considered Referee's concerns and revised the manuscript accordingly to clarify our interpretations, strengthen the relevant analyses, and more explicitly discuss the limitations and possible explanations for the observed genetic-background-dependent phenotypes.

Major points

1. Evidence for coordination of class I and class II crossover pathways

A major conclusion of this study is that ZEP1 plays an important role in coordinating class I and class II CO pathways. The authors provide evidence that ZEP1 directly interacts with anti-CO factors and is required for their proper chromosomal association, supporting a model in which ZEP1 helps prevent excessive strand invasion and limits inappropriate recombination outcomes that may otherwise favor class II CO formation. This is an interesting and well-supported aspect of the study.

However, if the authors wish to argue that ZEP1 coordinates the balance between both class I and class II pathways, it would be important to further clarify whether ZEP1 also has direct or functional connections with class I CO factors. At present, the evidence appears stronger for a role in restraining aberrant or excess recombination through anti-CO machinery than for a direct role in coordinating both pathways symmetrically. If additional interaction, localization, or genetic evidence regarding class I CO factors is available, inclusion of such data would substantially strengthen this conclusion.

Otherwise, the authors may wish to frame this point somewhat more cautiously in the text.

Response: Thanks for your helpful suggestion. In the newly generated data, we examined the interaction between ZEP1 and ZMM proteins using Y2H and BiFC assays, and detected positive interactions (please see Appendix Figure S10A-B) in both assays. Furthermore, loss of ZEP1 primarily prolongs the chromosomal association of ZMM proteins rather than affecting their initial recruitment (see Figure EV2B-C in revised manuscript), suggesting prolonged persistence of ZMM-associated recombination intermediates in the mutant background. This increases the probability that recombination intermediates enter the ZIP4-marked pro-CO pathway under conditions of elevated strand invasion activity. Combined with the finding that loss of ZMM proteins suppresses the chromosome association and fragmentation phenotypes of the *zep1* mutant, as well as the antagonistic genetic relationship between anti-CO proteins and ZMM proteins at the CO precursor stage, we therefore propose that ZEP1 coordinates the balance between class I and class II crossover pathways. Corresponding description and discussion have been included in Lines 307-315 and 519-526 of the revised manuscript.

2. Interpretation of the background-dependent effects

The comparison across japonica, indica, and hybrid backgrounds is potentially valuable and may reveal that SC-dependent recombination outcomes are highly sensitive to genome context. This is an interesting aspect of the manuscript. However, the mechanistic interpretation currently appears to go somewhat beyond the data presented. In particular, statements suggesting that ZEP1 is embedded in an "evolving recombination network" are plausible, but at present remain interpretive unless supported by more direct comparative genetic or molecular evidence.

This section would benefit from a tighter linkage between the observed phenotypic differences and the conclusions drawn from them. In particular, the Discussion should more clearly acknowledge the limitation that the basis of these background-dependent effects remains unresolved. A more measured discussion of this point would improve the balance of the manuscript.

Response: Thanks for your helpful correction. We agree that, although the comparison among japonica, indica, and hybrid backgrounds is informative and suggests that ZEP1-dependent recombination outcomes are sensitive to genomic context, the mechanistic basis of these background-dependent effects is not resolved by the current data.

We have therefore revised the Discussion to present this point more cautiously. In particular, we have tempered statements implying that ZEP1 is definitively embedded in an "evolving recombination network" and now more directly link our conclusions to the observed phenotypic differences. We also explicitly acknowledge that the molecular basis of background-dependent recombination variation remains to be determined. Please see Lines 574-577 in the revised manuscript.

Minor points

1. Lines 321-322: "In rice, zmm mutants complete synapsis but form many univalents at metaphase I." The data shown in Figure 4 appear to be consistent with this statement. However, statistical evaluation of chiasma number would be helpful to further support this conclusion and to make the comparison more rigorous.

Response: Thanks for your helpful suggestion. We have performed a statistical analysis of chiasma numbers in both the *msh5* and *shoc1* single mutants. Our results show that chiasma frequencies are significantly reduced in both mutants, consistent with previous reports. Please see Lines 292-294 in the revised manuscript.

2. Lines 375-376 and 403-404: Figure S13 and Figure S14 show defective loading patterns of MEICA1/FIGNL1 and RMI1 in zep1 null-2, similar to those observed in zep1 null-1. It would be helpful if the authors could provide quantitative estimates of the proportion of cells showing reduced signals, as well as the proportion showing clear signal mislocalization. Such quantification would strengthen the conclusion that the phenotype is reproducible across independent null alleles.

Response: Thanks for your helpful suggestion. As suggested, we have included a statistical analysis of the numbers of cells exhibiting different defect categories of MEICA1/FIGNL1/RMI1 foci in the *zep1^{null}-2* mutant. The new quantifications are included in the internal legends of Figure EV5A and Appendix Figure S11 in the revised manuscript.

Dr. Wanqi Liang
Shanghai Jiao Tong University
School of Life Science and Biotechnology
Shanghai
China

26th Aug 2026

Re: EMBOJ-2026-124191R
ZEP1 orchestrates template choice and crossover pathways to ensure meiotic genome integrity in rice

Dear Dt. Liang,

Thank you for submitting your revised manuscript on ZEP1 safeguarding meiotic genome integrity to The EMBO Journal. All three original referees have now reviewed it once more, and overall found the study significantly strengthened and most major concerns adequately addressed. Referee 2 retains a minor presentational reservation, which I would ask you to incorporate in a final round of minor revision. In addition, there are still a few editorial modifications to be made at this stage:

- Importantly, our routine pre-acceptance image checks indicated a potentially inadvertent image duplication within Figure 4D. Furthermore, we noted a re-display of micrographs between Figure 5C and Appendix Figure S11, and between Figure 6AD and Figure EV5AB, respectively, neither of which is however explicitly mentioned and rationalized in the respective figure legends. Therefore, please carefully double-check and clarify/correct - in cases where data are from the same experiment and the EV or Appendix data simply provide a more comprehensive comparison related to the main figure, this would nevertheless have to be clear indicated in each of the corresponding figure legends.

- Since we mandate addition of ORCID identifiers for all corresponding authors, please obtain and add an ORCID to your author profile in our submission system. This needs to be added by you personally and can unfortunately not be done by our office on your behalf.

- Please adjust the order as well as the headers of the different manuscript sections: Title page with complete author information, Abstract, Introduction, Results, Discussion, Methods, Data Availability, Acknowledgements, Disclosure and Competing Interests Statement, References, Main Figure Legends, Main Tables, Expanded View Figure Legends

- Please double-check all in-text figure references, making sure that they appear in correct numerical and alphabetical order.

- As we have switched from a free-text author contribution statement towards a more formal statement based on Contributor Role Taxonomy (CRediT) terms, please remove the present Author Contribution section and instead specify each author's contribution(s) directly in the Author Information page of our submission system during upload of the final manuscript. See https://casrai.org/credit/ for more information.

- Please provide the exact p values in the legends of figures 1D, 2C, EV2 C, EV4 D

- Finally, please provide suggestions for a short 'blurb' text prefacing and summing up the conceptual aspect of the study in two sentences (max. 250 characters), followed by 3-5 one-sentence 'bullet points' with brief factual statements of key results of the paper; they will form the basis of an editor-written 'Synopsis' accompanying the online version of the article. Please also upload a synopsis image, which can be used as a "visual title" for the synopsis section of your paper. The image should be in JPG format and exactly 550 pixels wide and 300 pixels high, and please make sure that it remains clear enough even in these modest dimensions.

I am returning the manuscript to you for minor revision, hoping you will be able to return the final modified files within one week. Once we will have received them, we should hopefully be able to swiftly proceed with formal acceptance and production of the manuscript.

Yours sincerely,

Hartmut Vodermaier

Hartmut Vodermaier, PhD
Senior Editor, The EMBO Journal
h.vodermaier@embojournal.org

*** PLEASE NOTE: All revised manuscript are subject to initial checks for completeness and adherence to our formatting guidelines. Revisions may be returned to the authors and delayed in their editorial re-evaluation if they fail to comply to the following requirements. As a first step please read our guidelines for revised submissions:
https://link.springer.com/journal/44318/submission-guidelines#cms-Revised-submissions

1) Every manuscript requires a Data Availability section (even if only stating that no deposited datasets are included). Primary datasets or computer code produced in the current study have to be deposited in appropriate public repositories prior to resubmission, and reviewer access details provided in case that public access is not yet allowed.

2) Each figure legend must specify
- size of the scale bars that are mandatory for all micrograph panels
- the statistical test used to generate error bars and P-values
- the type error bars (e.g., S.E.M., S.D.)
- the number (n) and nature (biological or technical replicate) of independent experiments underlying each data point
- Figures may not include error bars for experiments with n<3; scatter plots showing individual data points should be used instead.

3) Revised manuscript text (including main tables, and figure legends for main and EV figures) has to be submitted as editable text file (e.g., .docx format). We encourage highlighting of changes (e.g., via text color) for the referees' reference.

4) Each main and each Expanded View (EV) figure should be uploaded as individual production-quality files (preferably in .eps, .tif, .jpg formats). For suggestions on figure preparation/layout, please refer to our Figure Preparation Guidelines:
https://media.springernature.com/original/springer-cms/rest/v1/content/27825798/data/v1

5) Point-by-point response letters should include the original referee comments in full together with your detailed responses to them (and to specific editor requests if applicable), and also be uploaded as editable (e.g., .docx) text files.

6) Please complete our Author Checklist, and make sure that information entered into the checklist is also reflected in the manuscript; the checklist will be available to readers as part of the Review Process File.

7) All authors listed as (co-)corresponding need to deposit, in their respective author profiles in our submission system, a unique ORCiD identifier linked to their name. Please see our Guide to Authors for detailed instructions.

8) Please note that supplementary information at EMBO Press has been superseded by the 'Expanded View' for inclusion of additional figures, tables, movies or datasets; with up to five EV Figures being typeset and directly accessible in the HTML version of the article.

9) To facilitate reproducibilty and cross-laboratory adoption of methodologies, please structure the Materials & Methods section as outlined in our guide to authors, including a completed Reagents and Tools Table.

10) Digital image enhancement is acceptable practice, as long as it accurately represents the original data and conforms to community standards. If a figure has been subjected to significant electronic manipulation, this must be clearly noted in the figure legend and/or the 'Materials and Methods' section. The editors reserve the right to request original versions of figures and the original images that were used to assemble the figure. Finally, we generally encourage uploading of numerical as well as gel/blot image source data.

At EMBO Press we ask authors to provide source data for the main manuscript figures. You will receive a separate email with instructions for providing source data with your revised manuscript, including how to upload and organize the files.

In the interest of ensuring the conceptual advance provided by the work, we recommend submitting a revision within 3 months (24th Nov 2026). Please discuss the revision progress ahead of this time with the editor if you require more time to complete the revisions. Use the link below to submit your revision:

Link Not Available

Referee #1:

The revised manuscript is significantly improved over the original version. Although the authors did not perform some of the

additional experiments I suggested, I understand and accept their explanation as to why these experiments were not feasible. The authors have toned down conclusions that were previously too far-reaching and have corrected the inaccuracies I pointed out. I am therefore satisfied with the revised version of the manuscript and recommend it for acceptance.

Referee #2:

I thank the authors for this revised version. It largely addresses most of my comments. The only remaining concern is the interpretation of the HEI10 data, which still goes beyond what can be concluded from the results. While HEI10 is a well-established marker of class I CO designation, the stabilization of HEI10 foci and their correlation with univalent frequency do not demonstrate that these sites mature into functional COs, particularly in mutants showing substantial chromosome fragmentation.

I therefore ask the authors to revise the manuscript to consistently distinguish HEI10-marked class I CO sites/intermediates from mature COs. In the absence of direct genetic recombination measurements, conclusions based on HEI10 foci should be framed in terms of CO designation or progression toward CO maturation, rather than mature or functional CO formation.

Referee #3:

The authors have done a great job to address my concerns and I am satisfied with the revised manuscript.

Referee #2:

I thank the authors for this revised version. It largely addresses most of my comments. The only remaining concern is the interpretation of the HEI10 data, which still goes beyond what can be concluded from the results. While HEI10 is a well-established marker of class I CO designation, the stabilization of HEI10 foci and their correlation with univalent frequency do not demonstrate that these sites mature into functional COs, particularly in mutants showing substantial chromosome fragmentation.

I therefore ask the authors to revise the manuscript to consistently distinguish HEI10-marked class I CO sites/intermediates from mature COs. In the absence of direct genetic recombination measurements, conclusions based on HEI10 foci should be framed in terms of CO designation or progression toward CO maturation, rather than mature or functional CO formation.

Response: We appreciate the referee for this helpful comment. We agree that the interpretation of the HEI10 data should be stated more precisely. Although HEI10 is a well-established marker of class I CO designation, the stabilization or persistence of HEI10 foci at designated sites does not, by itself, demonstrate that these sites mature into functional COs, particularly in *zep1* mutant background.

We have therefore carefully re-examined all sections of the manuscript in which HEI10 data are discussed and revised the wording to consistently distinguish HEI10-marked class I CO-designated sites/intermediates from mature or functional COs.

In particular, we have modified statements that could be interpreted as directly equating HEI10 foci with mature or functional CO formation. Conclusions based on HEI10 foci are now framed in terms of class I CO designation and progression of HEI10-marked intermediates toward class I CO maturation, rather than definitive mature CO formation.

These revisions have been incorporated throughout the revised manuscript, including the relevant Results and Discussion sections (please see revised Lines 18-19, 110-112, 186-187, 203-205, 236, 524-525, 567 and 586). We believe that the revised wording more accurately reflects the evidential basis of the HEI10 analyses and appropriately acknowledges the absence of direct genetic recombination measurements.

EMBO Press Author Checklist

Corresponding Author Name: Wanqi Liang	
Journal Submitted to: The EMBO Journal	
Manuscript Number: EMBOJ-2026-124191	

USEFUL LINKS FOR COMPLETING THIS FORM
The EMBO Journal - Author Guidelines
EMBO Reports - Author Guidelines
Molecular Systems Biology - Author Guidelines
EMBO Molecular Medicine - Author Guidelines

Reporting Checklist for Life Science Articles (updated January
This checklist is adapted from Materials Design Analysis Reporting (MDAR) Checklist for Authors. MDAR establishes a minimum set of requirements in transparent reporting in the life sciences (see Statement of Task: 10.31222/osf.io/9sm4x). Please follow the journal's guidelines in preparing your
Please note that a copy of this checklist will be published alongside your article.

Abridged guidelines for figures
1. Data
The data shown in figures should satisfy the following conditions:
- the data were obtained and processed according to the field's best practice and are presented to reflect the results of the experiments in an accurate and unbiased manner.
- ideally, figure panels should include only measurements that are directly comparable to each other and obtained with the same assay.
- plots include clearly labeled error bars for independent experiments and sample sizes. Unless justified, error bars should not be shown for technical
- if n<5, the individual data points from each experiment should be plotted. Any statistical test employed should be justified.
- Source Data should be included to report the data underlying figures according to the guidelines set out in the authorship guidelines on Data

2. Captions
Each figure caption should contain the following information, for each panel where they are relevant:
- a specification of the experimental system investigated (eg cell line, species name).
- the assay(s) and method(s) used to carry out the reported observations and measurements.
- an explicit mention of the biological and chemical entity(ies) that are being measured.
- an explicit mention of the biological and chemical entity(ies) that are altered/varied/perturbed in a controlled manner.
- the exact sample size (n) for each experimental group/condition, given as a number, not a range;
- a description of the sample collection allowing the reader to understand whether the samples represent technical or biological replicates (including how many animals, litters, cultures, etc.).
- a statement of how many times the experiment shown was independently replicated in the laboratory.
- definitions of statistical methods and measures:
  - common tests, such as t-test (please specify whether paired vs. unpaired), simple χ2 tests, Wilcoxon and Mann-Whitney tests, can be unambiguously identified by name only, but more complex techniques should be described in the methods section;
  - are tests one-sided or two-sided?
  - are there adjustments for multiple comparisons?
  - exact statistical test results, e.g., P values = x but not P values < x;
  - definition of 'center values' as median or average;
  - definition of error bars as s.d. or s.e.m.

Please complete ALL of the questions below.
Select "Not Applicable" only when the requested information is not relevant for your study.

Materials

Newly Created Materials	Information included in the manuscript?	In which section is the information available? (Reagents and Tools Table, Materials and Methods, Figures, Data Availability Section)
New materials and reagents need to be available; do any restrictions apply?	Yes	Reagents and Tools Table

Antibodies	Information included in the manuscript?	In which section is the information available? (Reagents and Tools Table, Materials and Methods, Figures, Data Availability Section)
For **antibodies** provide the following information:<br>- Commercial antibodies: RRID (if possible) or supplier name, catalogue number and or/clone number<br>- Non-commercial: RRID or citation	Yes	Reagents and Tools Table

DNA and RNA sequences	Information included in the manuscript?	In which section is the information available? (Reagents and Tools Table, Materials and Methods, Figures, Data Availability Section)
**Short novel DNA or RNA including primers, probes**: provide the sequences.	Yes	Reagents and Tools Table

Cell materials	Information included in the manuscript?	In which section is the information available? (Reagents and Tools Table, Materials and Methods, Figures, Data Availability Section)
**Cell lines:** Provide species information, strain. Provide accession number in repository **OR** supplier name, catalog number, clone number, and/**OR** RRID.	Not Applicable	
**Primary cultures:** Provide species, strain, sex of origin, genetic modification status.	Not Applicable	
Report if the cell lines were recently **authenticated** (e.g., by STR profiling) and tested for mycoplasma contamination.	Not Applicable	

Experimental animals	Information included in the manuscript?	In which section is the information available? (Reagents and Tools Table, Materials and Methods, Figures, Data Availability Section)
**Laboratory animals or Model organisms:** Provide species, strain, sex, age, genetic modification status. Provide accession number in repository **OR** supplier name, catalog number, clone number, **OR** RRID.	Not Applicable	
**Animal observed in or captured from the field:** Provide species, sex, and age where possible.	Not Applicable	
Please detail **housing and husbandry conditions**.	Not Applicable	

Plants and microbes	Information included in the manuscript?	In which section is the information available? (Reagents and Tools Table, Materials and Methods, Figures, Data Availability Section)
**Plants:** provide species and strain, ecotype and cultivar where relevant, unique accession number if available, and source (including location for collected wild specimens).	Yes	Reagents and Tools Table
**Microbes:** provide species and strain, unique accession number if available, and source.	Yes	Reagents and Tools Table

Human research participants	Information included in the manuscript?	In which section is the information available? (Reagents and Tools Table, Materials and Methods, Figures, Data Availability Section)
If collected and within the bounds of privacy constraints report on age, sex and gender or ethnicity for all study participants.	Not Applicable	

Core facilities	Information included in the manuscript?	In which section is the information available? (Reagents and Tools Table, Materials and Methods, Figures, Data Availability Section)
If your work benefited from core facilities, was their service mentioned in the acknowledgments section?	Yes	Acknowledgements

Design

Study protocol	Information included in the manuscript?	In which section is the information available? (Reagents and Tools Table, Materials and Methods, Figures, Data Availability Section)
If study protocol has been **pre-registered, provide DOI in the manuscript**. For clinical trials, provide the trial registration number **OR** cite DOI.	Not Applicable	
Report the **clinical trial registration number** (at ClinicalTrials.gov or equivalent), where applicable.	Not Applicable	

Laboratory protocol	Information included in the manuscript?	In which section is the information available? (Reagents and Tools Table, Materials and Methods, Figures, Data Availability Section)
Provide DOI OR other citation details if **external detailed step-by-step protocols** are available.	Not Applicable	

Experimental study design and statistics	Information included in the manuscript?	In which section is the information available? (Reagents and Tools Table, Materials and Methods, Figures, Data Availability Section)
Include a statement about **sample size** estimate even if no statistical methods were used.	Yes	Figures
Were any steps taken to minimize the effects of subjective bias when allocating animals/samples to treatment (e.g. **randomization procedure**)? If yes, have they been described?	Yes	Figures
Include a statement about **blinding** even if no blinding was done.	Not Applicable	
Describe **inclusion/exclusion criteria** if samples or animals were excluded from the analysis. Were the criteria pre-established?<br><br>If sample or data points were omitted from analysis, report if this was due to attrition or intentional exclusion and provide justification.	Not Applicable	
For every figure, are **statistical tests** justified as appropriate? Do the data meet the assumptions of the tests (e.g., normal distribution)? Describe any methods used to assess it. Is there an estimate of variation within each group of data? **I**s the variance similar between the groups that are being statistically compared?	Yes	Figures

Sample definition and in-laboratory replication	Information included in the manuscript?	In which section is the information available? (Reagents and Tools Table, Materials and Methods, Figures, Data Availability Section)
In the figure legends: state number of times the experiment was **replicated** in laboratory.	Not Applicable	Figures
In the figure legends: define whether data describe **technical or biological replicates**.	Yes	Figures

Ethics

Ethics	Information included in the manuscript?	In which section is the information available? (Reagents and Tools Table, Materials and Methods, Figures, Data Availability Section)
Studies involving **human participants**: State details of **authority granting ethics approval** (IRB or equivalent committee(s), provide reference number for approval.	Not Applicable	
Studies involving **human participants**: Include a statement confirming that **informed consent** was obtained from all subjects and that the experiments conformed to the principles set out in the WMA Declaration of Helsinki and the Department of Health and Human Services Belmont Report.	Not Applicable	
Studies involving **human participants:** For publication of **patient photos**, include a statement confirming that consent to publish was obtained.	Not Applicable	
Studies involving experimental **animals**: State details of **authority granting ethics approval** (IRB or equivalent committee(s), provide reference number for approval. Include a statement of compliance with ethical regulations.	Not Applicable	
Studies involving **specimen and field samples:** State if relevant **permits** obtained, provide details of authority approving study; if none were required, explain why.	Not Applicable	

Dual Use Research of Concern (DURC)	Information included in the manuscript?	In which section is the information available? (Reagents and Tools Table, Materials and Methods, Figures, Data Availability Section)
Could your study fall under dual use research restrictions? Please check biosecurity documents and list of **select agents and toxins** (CDC): https://www.selectagents.gov/sat/list.htm	Not Applicable	
If you used a select agent, is the security level of the lab appropriate and reported in the manuscript?	Not Applicable	
If a study is subject to dual use research of concern regulations, is the name of the **authority granting approval and reference number** for the regulatory approval provided in the manuscript?	Not Applicable	

Reporting

The MDAR framework recommends adoption of discipline-specific guidelines, established and endorsed through community initiatives. Journals have their own policy about requiring specific guidelines and recommendations to complement MDAR.

Adherence to community standards	Information included in the manuscript?	In which section is the information available? (Reagents and Tools Table, Materials and Methods, Figures, Data Availability Section)
State if relevant guidelines or checklists (e.g., **ICMJE, MIBBI, ARRIVE, PRISMA**) have been followed or provided.	Not Applicable	
For **tumor marker prognostic studies,** we recommend that you follow the **REMARK** reporting guidelines (see link list at top right). See author guidelines, under 'Reporting Guidelines'. Please confirm you have followed these guidelines.	Not Applicable	
For **phase II and III randomized controlled trials**, please refer to the **CONSORT** flow diagram (see link list at top right) and submit the CONSORT checklist (see link list at top right) with your submission. See author guidelines, under 'Reporting Guidelines'. Please confirm you have submitted this list.	Not Applicable	

Data Availability

Data availability	Information included in the manuscript?	In which section is the information available? (Reagents and Tools Table, Materials and Methods, Figures, Data Availability Section)
Have **primary datasets** been deposited according to the journal's guidelines (see 'Data Deposition' section) and the respective accession numbers provided in the Data Availability Section?	Yes	Data Availability Section
Were **human clinical and genomic datasets** deposited in a public access-controlled repository in accordance to ethical obligations to the patients and to the applicable consent agreement?	Not Applicable	
Are **computational models** that are central and integral to a study available without restrictions in a machine-readable form? Were the relevant accession numbers or links provided?	Not Applicable	
If publicly available data were reused, provide the respective **data citations in the reference list**.	Not Applicable	
