# Supplementary material for: ZEP1 orchestrates template choice and crossover pathways to ensure meiotic genome integrity in rice
Source: EMBO J. 2026 Sep 17;45(20):7163–90. doi: 10.1038/s44318-026-00910-0 (PMC13672304; doi:10.1038/s44318-026-00910-0)
Supplement: Supplementary file 3 [file 44318_2026_910_MOESM3_ESM.pdf]

Expanded View Figures

Figure EV1. ***zep1* null mutation leads to abnormal chromosome behaviors in all tested rice varieties.**

(**A**) Chromosome behavior during different stages of male meiosis in *zep1^{null}* CRISPR lines in *japonica* 9522 background (*zep1^{null}-2* and *zep1^{null}-3*). Abnormal chromosome connections are indicated by yellow arrows. Scale bar, 5 µm. Arabic numerals in the panels represent the number of representative images containing chromosome fragments relative to the total number of observations. A total of $n = 29$ *zep1^{null}-2*, $n = 28$ *zep1^{null}-3* anaphase I and $n = 5$ *zep1^{null}-2*, $n = 8$ *zep1^{null}-3* anaphase II meiocytes were scored from chromosome spreads of pooled anthers isolated from meiotic florets of independent isogenic plants. Multiple independent slide preparations were examined to confirm the reproducibility of the observed phenotypes. Meiotic chromosomes are visualized by DAPI staining. (**B**) No obvious ZEP1 protein foci (green) were detected on *zep1* male meiotic chromosomes. Meiotic chromosomes are visualized by axis protein REC8 (magenta). Scale bar, 2 µm. (**C**) Chromosome behavior during different stages of male meiosis in *zep1* mutants from different genetic backgrounds. Meiotic defects are indicated by white arrows (univalents) and white arrowheads (chromosome fragments), respectively. Meiotic chromosomes are visualized by DAPI staining. Arabic numerals in the panels represent the number of representative images containing chromosome fragments relative to the total number of observations. A total of $n = 76$ *zep1^{Nipponbare}*, $n = 12$ *zep1^{Kasalath}*, $n = 14$ *zep1$^{Minghui\ 63}$*, $n = 22$ *zep1$^{Zhenshan\ 97}$*, $n = 32$ *zep1$^{Chunyou\ 84}$* anaphase I meiocytes were scored from chromosome spreads of pooled anthers isolated from meiotic florets of independent isogenic plants. Multiple independent slide preparations were analyzed. Scale bar, 5 µm. Source data underlying this figure are provided online.

© The Author(s)

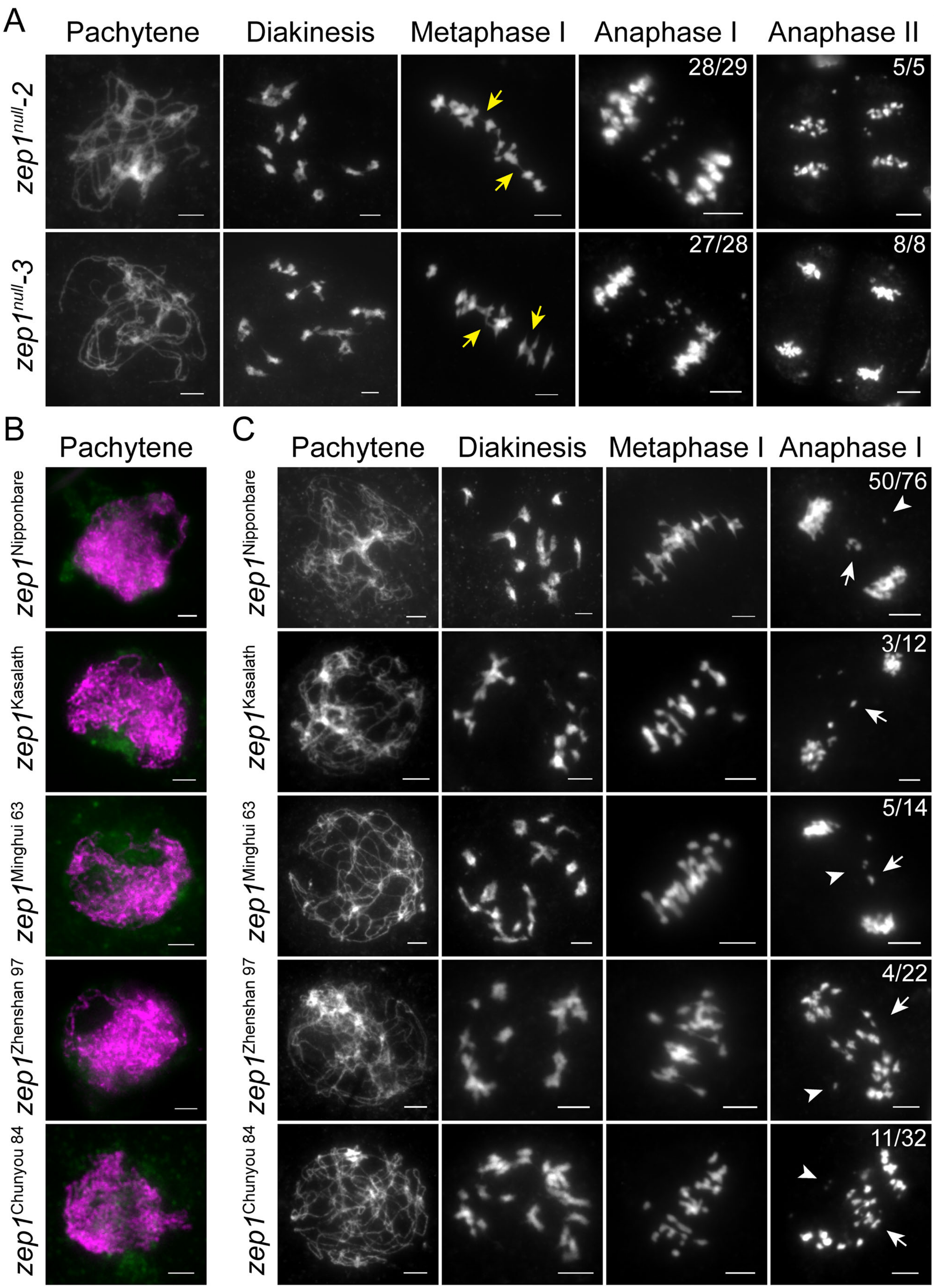
A
Pachytene
Diakinesis
Metaphase I
Anaphase I
Anaphase II
zep1null-2
zep1null-3
28/29
5/5
27/28
8/8
B
Pachytene
C
Pachytene
Diakinesis
Metaphase I
Anaphase I
zep1Nipponbare
zep1Kasalath
zep1Minghui 63
zep1Zhenshan 97
zep1Chunyou 84
50/76
3/12
5/14
4/22
11/32

 © The Author(s)

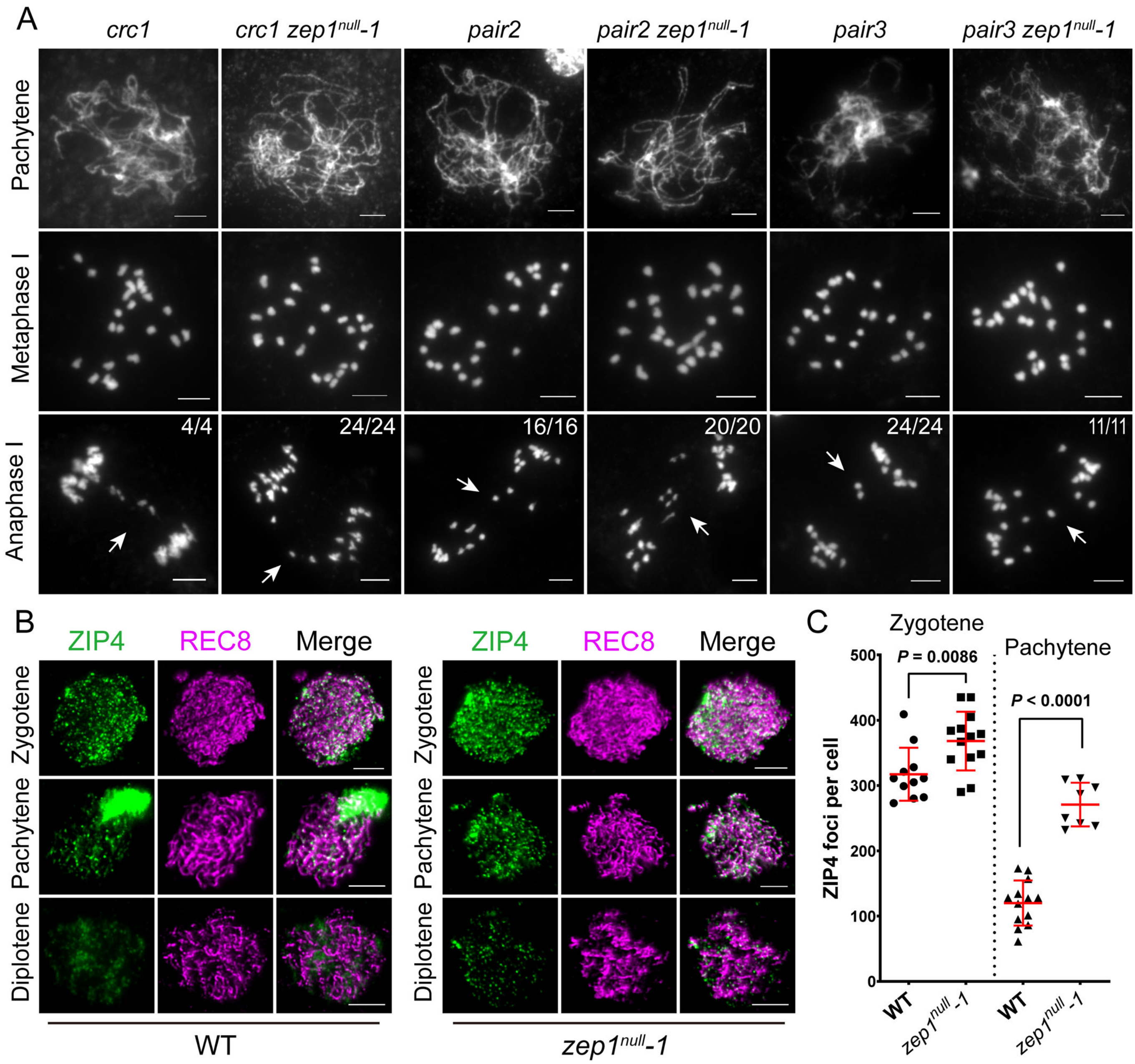

Figure EV2. ZEP1 acts downstream of homologous pairing and modulates the chromosomal localization of ZIP4 protein during male meiosis.

(**A**) Representative chromosome behavior from pachytene (or pachytene-like) to anaphase I in key meiosis-related single mutants and ZEP1-associated double mutants. Meiotic defects are indicated by white arrows (univalents) and white arrowheads (chromosome fragments), respectively. Arabic numerals in the panels represent the number of representative images containing univalents/chromosome fragments relative to the total number of observations. A total of $n = 4$ *crc1*, $n = 24$ *crc1 zep1^{null}-1*, $n = 16$ *pair2*, $n = 20$ *pair2 zep1^{null}-1*, $n = 24$ *pai3* and $n = 11$ *pair3 zep1^{null}-1* anaphase I meiocytes were scored from chromosome spreads generated via multiple independent slide preparations using pooled anthers isolated from meiotic florets collected from independent plants of the same genotype. Meiotic chromosomes are visualized by DAPI staining. Scale bars, 5 μm. (**B**) Immunofluorescent signals of the ZMM protein ZIP4 (green) were detected in wild-type and *zep1^{null}-1* male meiocytes at different substages of meiotic prophase I. Meiotic chromosomes are visualized by axis protein REC8 (magenta). Scale bars, 2 μm. (**C**) ZIP4 foci numbers in wild-type and *zep1^{null}* meiocytes at zygotene and pachytene (or pachytene-like) stages. A total of $n = 11$ WT zygotene meiocytes, $n = 13$ *zep1^{null}-1* zygotene meiocytes, $n = 13$ WT pachytene meiocytes, $n = 8$ *zep1^{null}-1* pachytene-like meiocytes were scored from chromosome spreads generated using pooled anthers dissected from meiotic florets collected from independent plants of the same genotype. Multiple independent slide preparations were examined to confirm the reproducibility of the observed phenotypes, with all quantified cells incorporated into the plotted dataset. Each dot corresponds to a single meiocyte. Bars denote mean ± SD of the quantified cell population. Statistical significance was determined via a two-tailed Student's *t*-test. Statistical *P* values derived from the corresponding quantitative analyses are indicated within the figure panels. Exact *P* values are as follows: WT versus *zep1^{null}-1* at zygotene, $P = 0.0086$; and WT versus *zep1^{null}-1* at pachytene, $P < 0.0001$. For comparisons in which GraphPad Prism returned *P* values of <0.0001, values are reported as $P < 0.0001$. Source data underlying this figure are provided online.

© The Author(s)

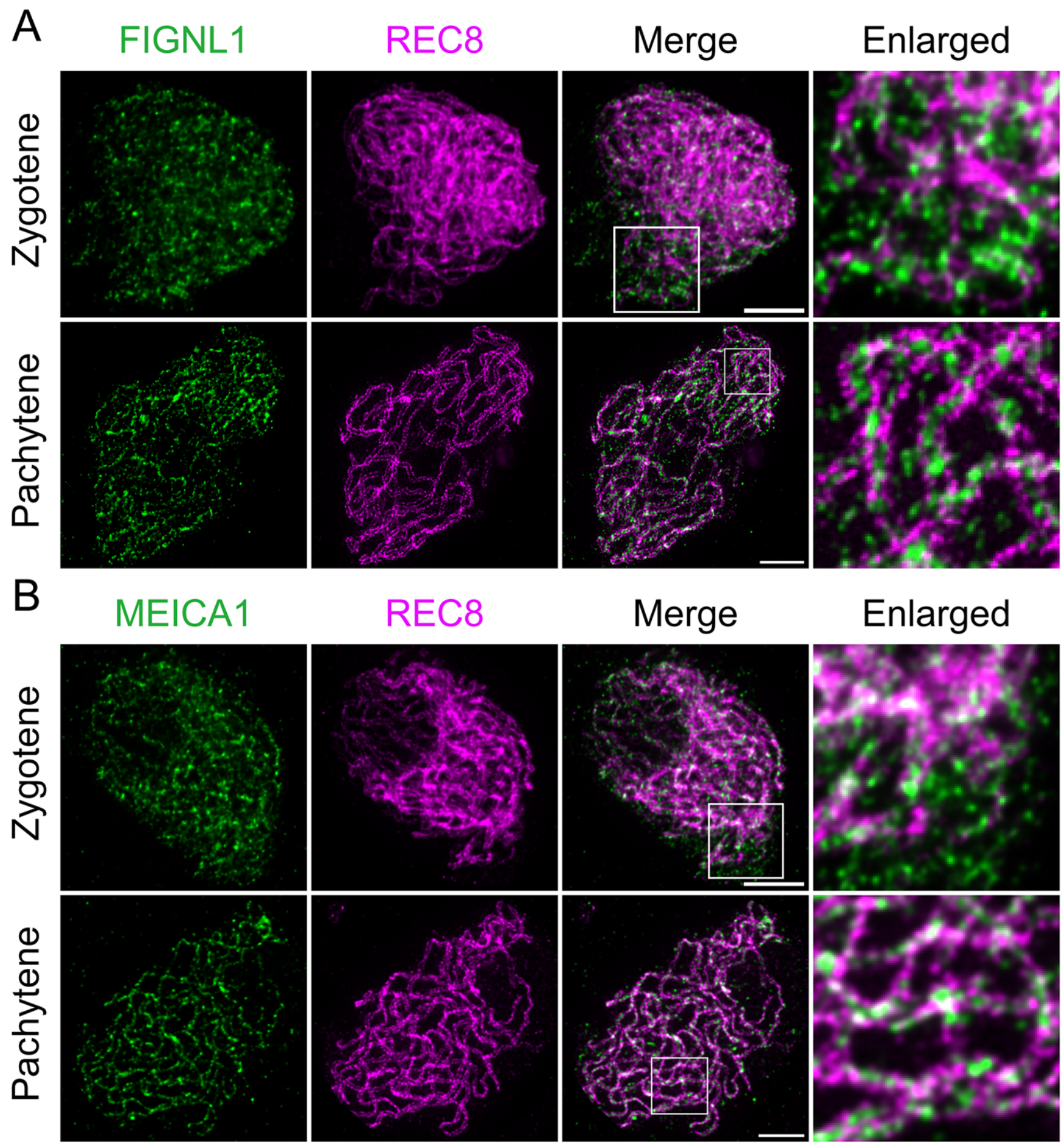

Figure EV3. Dynamic co-localization of MEICA1 and FIGNL1 with the meiotic chromosome axis marker REC8 during prophase I.

Stimulated emission depletion (STED) super-resolution microscopy of wild-type male prophase I meiocytes co-stained for the axis marker REC8 (magenta) together with FIGNL1 (**A**) or MEICA1 (**B**) (green) at zygotene and pachytene stages. Magnified insets of bracketed regions are shown on the right. Scale bars, 2 μm. Source data underlying this figure are provided online.

 © The Author(s)

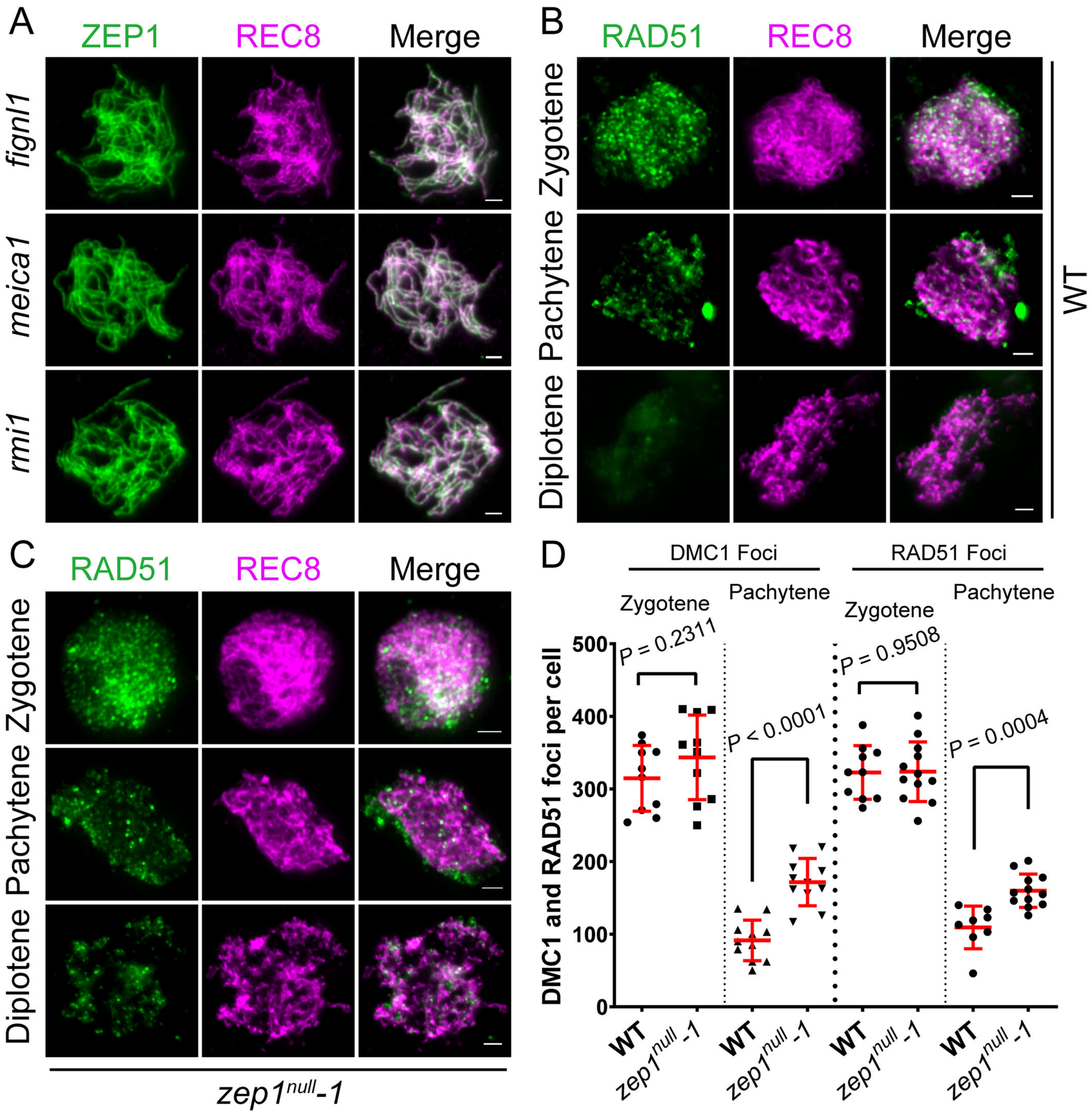

Figure EV4. ZEP1 depletion delays dissociation of strand-invasion proteins from male meiotic chromosomes.

(**A**) Mutations in MEICA1, FIGNL1 or RMI1 do not alter chromosomal loading of ZEP1 (green). Meiotic chromosomes are visualized by axis protein REC8 (magenta). Scale bars, 2 μm. (**B**, **C**) Immunofluorescent detection of the strand-invasion protein RAD51 (green) and meiotic chromosome axes (anti-OsREC8, magenta) in wild-type (**B**) and mutant (**C**) at different substages of meiotic prophase I. Scale bars, 2 μm. (**D**) DMC1 and RAD51 foci numbers in wild-type and *zep1^{null}-1* meiocytes at zygotene and pachytene (or pachytene-like) stages. A total of $n = 10$ WT zygotene meiocytes, $n = 10$ *zep1^{null}-1* zygotene meiocytes, $n = 11$ WT pachytene meiocytes, and $n = 11$ *zep1^{null}-1* pachytene-like meiocytes were scored for DMC1 foci. For RAD51 foci, $n = 10$ WT zygotene meiocytes, $n = 12$ *zep1^{null}-1* zygotene meiocytes, $n = 8$ WT pachytene meiocytes, and $n = 12$ *zep1^{null}-1* pachytene-like meiocytes were scored. Meiocytes were scored from chromosome spreads generated using pooled anthers dissected from meiotic florets collected from independent plants of the same genotype. Multiple independent slide preparations were examined to confirm the reproducibility of the observed phenotypes, with all quantified cells incorporated into the plotted dataset. Each dot corresponds to a single meiocyte. Bars denote mean ± SD of the quantified cell population. Statistical significance was determined via a two-tailed Student's *t*-test. Statistical *P* values derived from the corresponding quantitative analyses are indicated within the figure panels. Exact *P* values are as follows: for DMC1 foci, WT versus *zep1^{null}-1* at zygotene, $P = 0.2311$, and at pachytene, $P < 0.0001$; for RAD51 foci, WT versus *zep1^{null}-1* at zygotene, $P = 0.9508$, and at pachytene, $P = 0.0004$. For comparisons in which GraphPad Prism returned *P* values of <0.0001, values are reported as $P < 0.0001$. Source data underlying this figure are provided online.

© The Author(s)

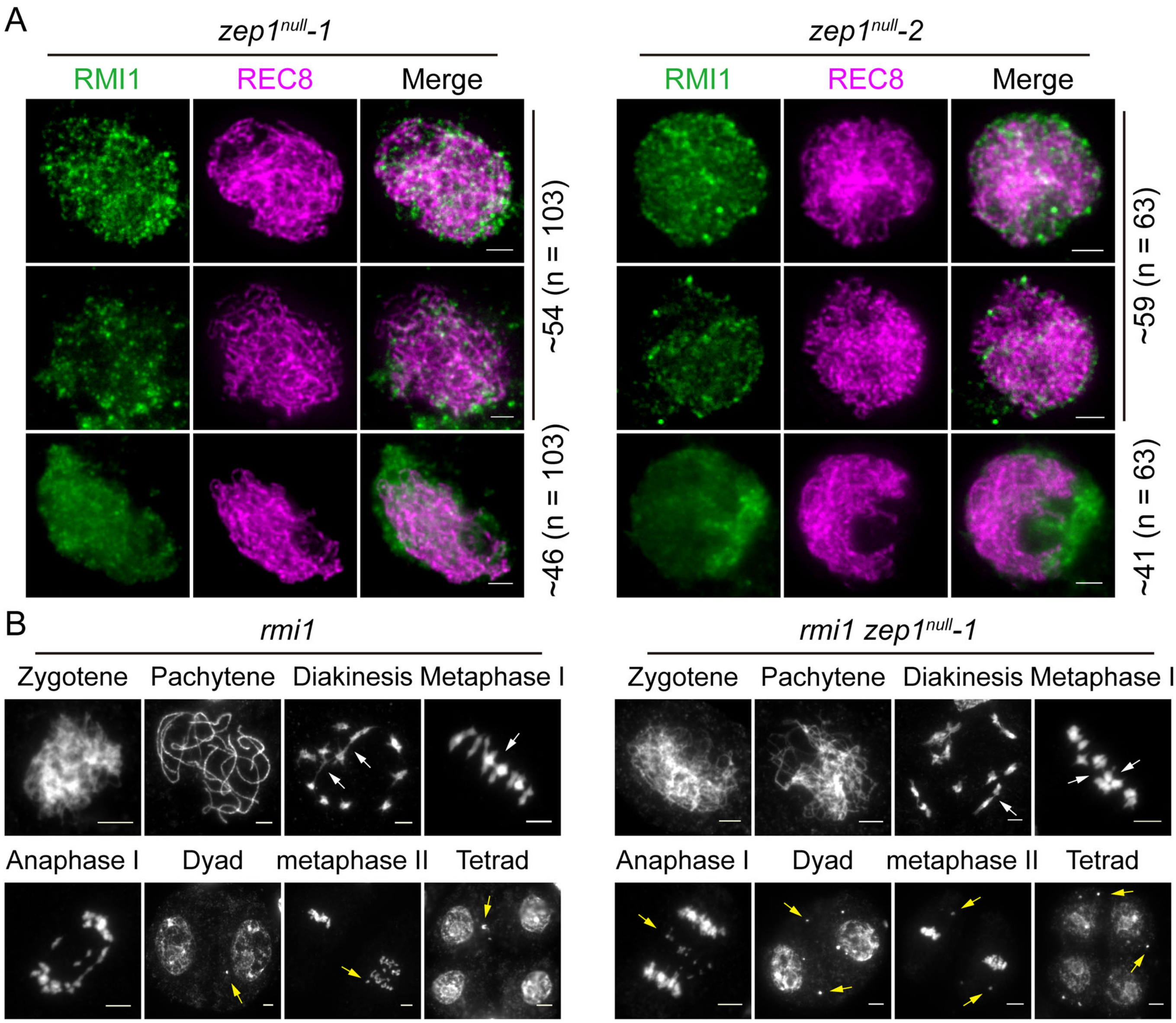

Figure EV5. ZEP1 is required for proper chromosomal enrichment of RMI1 during male meiosis.

(**A**) Representative images illustrating reduced chromosomal association and ectopic localization of RMI1 protein foci (green) in *zep1* null meiocytes. Meiotic chromosomes are visualized by axis protein REC8 (magenta). Representative images of RMI1 localization in *zep1^{null}-1* meiocytes were obtained from the same experiment as the images shown in Fig. 6A. Additional images are provided here for a more comprehensive comparison. "n" in the panels represents the number of meiocytes displaying the indicated phenotype over the total number of analyzed meiocytes in each group. A total of $n = 103$ *zep1^{null}-1* and $n = 63$ *zep1^{null}-2* prophase I meiocytes were generated via multiple independent slide preparations using pooled anthers isolated from meiotic florets collected from independent plants of the same genotype. Scale bar, 2 μm. (**B**) Chromosome behavior during different stages of *rmi1* and *rmi1 zep1^{null}-1* mutants. Meiocytes were scored from chromosome spreads generated via multiple independent slide preparations using pooled anthers isolated from meiotic florets collected from independent plants of the same genotype. Meiotic chromosomes are visualized by DAPI staining. Abnormal chromosome connections and chromosome bridge, lagging or missing chromosomes are indicated by white and yellow arrows, respectively. Scale bar, 5 μm. Representative images of chromosome behavior in *rmi1* and *rmi1 zep1^{null}-1* meiocytes were obtained from the same experiment as the images shown in Fig. 6D. Additional representative examples from different meiotic stages are provided here for a more comprehensive comparison. Source data underlying this figure are provided online.

© The Author(s)
